# Rectify the impact of shorter red blood cell lifespan upon HbA1c detection values in T2DM patients: modeling and internal-external verification

**DOI:** 10.3389/fendo.2025.1500660

**Published:** 2025-04-15

**Authors:** Li Zhang, Ximan Gao, Xuying Meng, Guangyang Ma, Jing Li, Weilin Wang, Sisi Chen, Yongjian Ma, Pei Yu, Saijun Zhou

**Affiliations:** ^1^ NHC Key Laboratory of Hormones and Development, Chu Hsien-I Memorial Hospital and Tianjin Institute of Endocrinology, Tianjin Medical University, Tianjin, China; ^2^ Tianjin Key Laboratory of Metabolic Diseases, Tianjin Medical University, Tianjin, China; ^3^ Department of Endocrinology, The Second Hospital of Tianjin Medical University, Tianjin, China; ^4^ Guangdong Breath Test Engineering and Technology Research Center, Shenzhen University, Shenzhen, China

**Keywords:** glycosylated hemoglobin (HbA1c), hemoglobin glycation index (HGI), red blood cell lifespan, type 2 diabetes mellitus, correction model

## Abstract

**Aims:**

To determine the effect of red blood cell (RBC) lifespan variability on glycosylated hemoglobin (HbA1c) measurements in type 2 diabetes mellitus (T2DM) individuals and develop a mathematical model for adjusting HbA1c values.

**Methods:**

We tracked glucose levels in 516 T2DM patients from Chu Hsien-I Memorial Hospital, categorized into Construction (n = 416) and Internal (n = 100) cohorts. Additionally, 165 participants from Tianjin diabetic retinopathy screening cohort, serving as the Independent cohort. RBC lifespan was determined using the CO breath test, and Hemoglobin glycation variation index (HGI) was calculated from the difference between measured and estimated HbA1c (eHbA1c). Model efficacy was evaluated using AUC, accuracy, sensitivity, and specificity.

**Results:**

An inflection in the HGI-RBC lifespan model occurred at 66 days, with HbA1c underestimation when RBC lifespan was below 90 days, notably in the ≤ 66 days group. This underestimation increased the risk of cardiovascular and peripheral neuropathy complications. To rectify the impact of the shorter RBC lifespan in T2DM patients, the correction formula was established as HbA1c(c) = -0.05629×RBC lifespan + 1.127×HbA1c + 3.178 (R = 0.7360) in the ≤ 66 day lifespan group and HbA1c(c) = -0.004772 × RBC lifespan + 0.7569 × HbA1c + 2.394 (R = 0.7344) in the 67 to 89 day group. The corrected HbA1c models exhibited satisfactory predictive performance in all cohorts.

**Conclusions:**

Accurate adjustment for the effects of RBC lifespan on HbA1c values in T2DM patients is expected to enhance blood glucose management and the efficacious prevention and treatment of diabetes-associated complications.

**Clinical Trial Registration:**

Chu Hsien-I Memorial Hospital of Tianjin Medical University, identifier ChiCTR2100046557.

## Introduction

Type 2 diabetes mellitus (T2DM) poses a significant threat to global human health. The prevalence of diabetes among adults is increasing, standing at 8.8% in 2017 and projected to escalate to 9.9% by 2045 (1). Glycosylated hemoglobin (HbA1c) is acknowledged as the golden criterion for appraising glycemic control during the past two to three months and is widely employed in clinical contexts (2). Clinical evidence reveals that HbA1c markedly influences the risk of chronic complications and the long-term prognosis for individuals with T2DM (3–5). Therefore, accurate assessment of HbA1c is an important basis for guiding clinical adjustment of anti-hyperglycemia treatment. Growing evidence showed that HbA1c levels could be affected by various conditions, especially by the modification of red blood cell (RBC) lifespan and glycation (1, 2). Glucose monitoring methods include Self-Measured Blood Glucose (SMBG), involving periodic fingerstick blood glucose testing, and Continuous Glucose Monitoring (CGM) systems, which employ subcutaneous sensors to provide real-time glucose data (6). Patients’ SMBG records were guided by a team of diabetes specialists, education nurses, and dietitians following the “Trinity Care” management model. Meanwhile, we employed the RBC lifespan tester (ELS Tester, Seekya Biotec Co., Ltd., Shenzhen, China), an innovation by Ma et al. (7–9), to ascertain the RBC lifespan in T2DM patients and subsequently scrutinized the interplay among HbA1c measurement values, average glucose (AG), and RBC lifespan. The CO breath test serves as a dependable and simplified approach for the rapid determination of human RBC lifespan, suitable for clinical use (9), which provides a powerful tool for rapid, accurate, noninvasive, safe, and economical detection of patients’ RBC lifespan (9, 10).

Hemoglobin glycation variation index (HGI) serves as a pivotal metric for gauging the divergence of HbA1c measurement values from the estimated equivalents, which was calculated as HGI = observed HbA1c - estimated HbA1c (eHbA1c) (11). Studies have confirmed that elevated HGI levels are robust predictors of cardiovascular events, Diabetic Kidney Disease, and overall mortality in T2DM individuals (12–14). Our preliminary investigation, albeit with a limited sample size of T2DM patients exhibiting HbA1c detection values below 7%, demonstrated that a diminished RBC lifespan leads to a significant underestimation of blood glucose levels as indicated by the measured HbA1c values (15).

In this study, we conducted a muticenter cohort study and enlarged the sample size, delineated the extent to which RBC lifespan influenced HbA1c measurements, and further established calculation formulas for correcting the impact of RBC lifespan on the HbA1c detection value, HbA1c(c) = -0.05629×RBC lifespan + 1.127×HbA1c + 3.178 (R = 0.7360) in the ≤ 66 day lifespan group and HbA1c(c) = -0.004772 × RBC lifespan + 0.7569 × HbA1c + 2.394 (R = 0.7344) in the 67 to 89 day group. The corrected HbA1c models achieved encouraging predictive performance in all cohorts, promising to deliver enhanced precision in glycemic assessments for clinical applications.

## Methods

### Participants and study design

**Figure 1:**
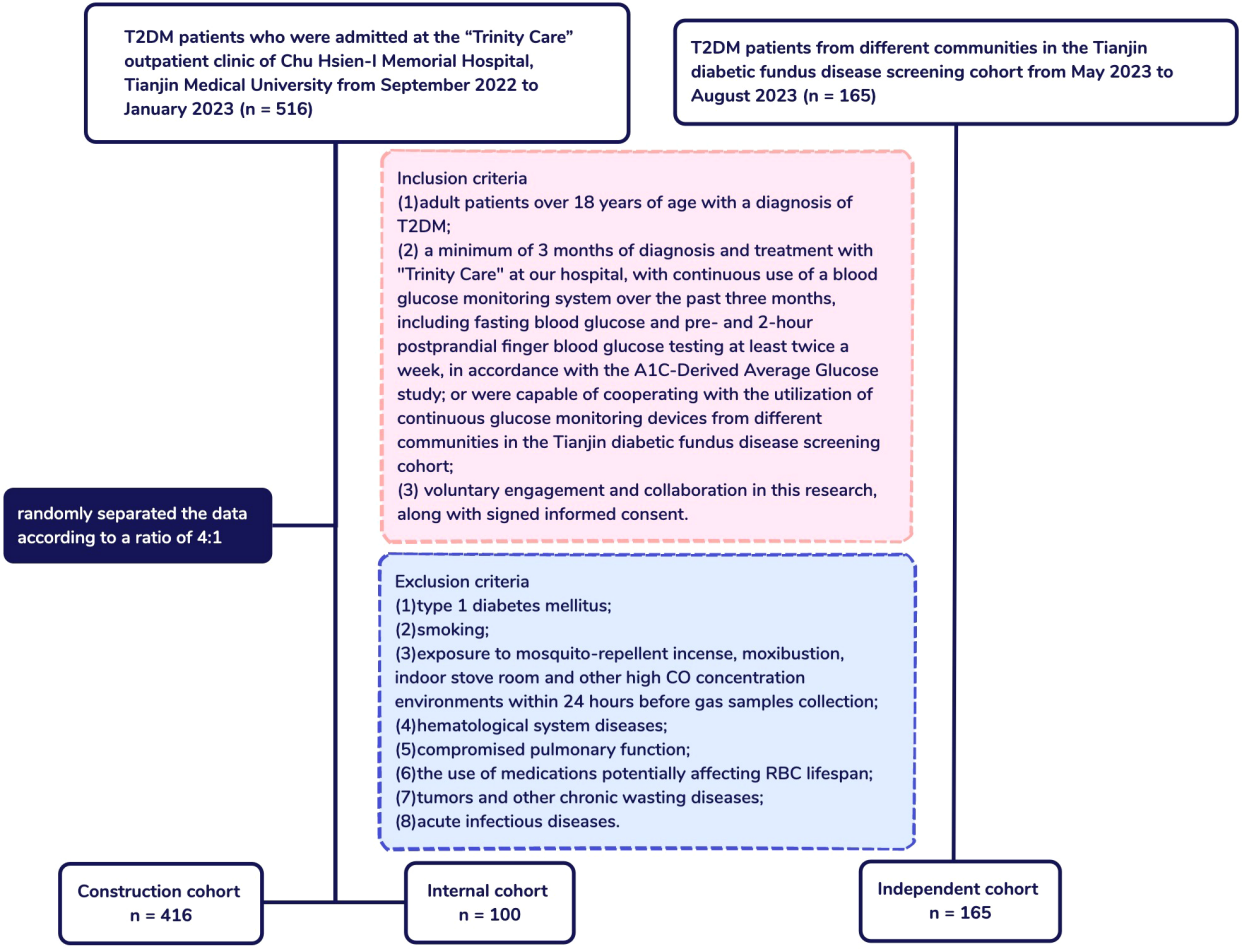
Flowchart of patients and study design. T2DM, Type 2 diabetes mellitus; ADAG, A1C-Derived Average Glucose; CO, Carbon monoxide.

This multicenter cohort study enrolled 516 T2DM patients from the “Trinity Care” outpatient clinic at Chu Hsien-I Memorial Hospital, Tianjin Medical University, from September 2022 to January 2023, and 165 T2DM patients from various communities within the Tianjin diabetic fundus disease screening cohort, observed from May 2023 to August 2023. Patients were divided into three cohorts: a construction cohort (n = 416 from Chu Hsien-I Memorial Hospital, Tianjin Medical University), an internal cohort (n = 100 from Chu Hsien-I Memorial Hospital, Tianjin Medical University), and an independent cohort (n = 165 from Tianjin diabetic fundus disease screening cohort). The flowchart of the study was presented in **Figure 1**.

The inclusion criteria encompassed the following: (1) patients aged 18 years or older with a diagnosis of T2DM; (2) a minimum duration of 3 months of diagnosis and treatment with “Trinity Care” at our hospital, with continuous use of a blood glucose monitoring system for the preceding three months, including fasting blood glucose and pre- and 2-hour postprandial finger blood glucose testing at least twice weekly, in accordance with the A1C-Derived Average Glucose study (16); or were capable of cooperating with the utilization of continuous glucose monitoring devices from different communities in the Tianjin diabetic fundus disease screening cohort; (3) voluntary engagement and collaboration in this research, along with provision of signed informed consent. The exclusion criteria included: (1) type 1 diabetes mellitus; (2) smoking; (3) exposure to mosquito-repellent incense, moxibustion, indoor stove room and other high CO concentration environments within 24 hours before gas samples collection; (4) hematological system diseases; (5) compromised pulmonary function; (6) the use of medications potentially affecting RBC lifespan; (7) tumors and other chronic wasting diseases; (8)acute infectious diseases.

This study was registered with the online Chinese Clinical Trial Registry under the identifier ChiCTR2100046557 and was approved by the ethics committee of Chu Hsien-I Memorial Hospital affiliated with Tianjin Medical University (Tianjin, China). Written informed consents were obtained from all patients prior to their participation in the study.

### Data acquisition and laboratory inspection

We garnered clinical data, encompassing age, gender, diabetes duration, medications, and incidence of complications (fundus lesions, cardiovascular diseases, peripheral neuropathy). A Beckman Coulter AU5800 automated biochemical analyzer (Beckman Coulter, Brea, CA, USA) was employed to assay liver and kidney functions, blood lipid concentrations, and other biochemical indicators. The HbA1c levels of patients were determined by means of TOSOH G8 (Tosoh Bioscience, Tokyo, Japan). Chu Hsien-I Memorial Hospital patients’ SMBG were recorded by iHealth Smart blood glucose meter under the guidance of a team of diabetes specialists, education nurses, and dietitians following the “Trinity Care” management model. Different communities Patients’ CGM were conducted by “Sibright” CGM system. The AG was translated to the eHbA1c in accordance with the formula: eHbA1c = (AG + 2.5944)/1.5944 (16). The HGI was computed as the discrepancy between the measured and the eHbA1c (HGI = measured HbA1c - eHbA1c) (11). Alveolar gas samples were also collected to measure RBC lifespan by trained personnel.

### RBC lifespan measurement

For the measurement of RBC lifespan using Levitt’s CO breath test, the procedure followed a protocol from a recently published study (9). In conclusion, gas samples from the alveoli of each participant were obtained between 8:00 and 10:00 AM after a period of overnight fasting and subsequent 20-minute rest. Participants were directed to inhale deeply, retain their breath for 10 seconds, and then exhale into the collection apparatus via a mouthpiece. The ELS Tester, an automated device manufactured by Seekya Biotec Co., Ltd., Shenzhen, China, was utilized to assess CO levels. This data was subsequently applied to compute the RBC lifespan employing Levitt’s methodology, as follows:


$$\text{RBCspan }=\;\frac{4\;\times \;\left[\text{Hb}\right]\;\times \;22400}{0.7\;\times \text{ endoPco }\times \;64400\;\times \;1440}\;\times \;\frac{\text{Vb}}{\text{Vt}}$$

(9).

### Statistical analysis

Multiple imputation was used to impute missing data for age, liver and kidney function, and blood lipid concentrations. Construction cohort and Internal cohort allocation were in a 4:1 ratio, achieved via simple randomization within the SPSS. The normalcy of data distribution was evaluated using the Kruskal-Wallis test, while group differences were compared utilizing the Chi-square test. The relationship between continuous variables was modeled using Spearman correlation analysis and restrictive spline regression. For estimating the correction formulas, multivariate linear regression models were engaged. The performance of the correction formulas were verified by ROC curve and calibration curve.

The statistical analyses were performed using IBM SPSS version 25.0, GraphPad Prism version 9.1.1, and R version 4.0.3. A p-value of less than 0.05 from two-tailed tests was deemed to indicate statistical significance.

## Results

### Cohort characteristics

Based on the inclusion and exclusion criteria, our study included 516 T2DM patients who were attendees at the “Trinity Care” outpatient clinic of Chu Hsien-I Memorial Hospital and 165 T2DM patients from various communities in the Tianjin diabetic fundus disease screening cohort, respectively. In the 516‐patient cohort, 416 and 100 patients were randomly assigned to the construction and internal cohorts, respectively (**Figure 1**). **Table 1** exhibited the traits of the study participants.

**Table 1:** General clinical characteristics of the construction, internal, and independent cohorts.

	Construction cohort (n = 416)	Internal cohort (n = 100)	Independent cohort (n = 165)
**Age(year)**	58 (48, 65)	60 (51, 66)	67 (64, 70)
**Gender (male,%)**	218 (52.4)	44 (44.0)	74 (44.8)
**Duration (year)**	7 (2, 12)	7 (4, 12)	10 (5, 15)
**Hb (g/l)**	145 (133, 158)	145 (134, 156)	139 (129, 149)
**TG (mmol/l)**	1.49 (1.12, 2.20)	1.53 (1.11, 2.21)	1.58 (1.06, 2.31)
**TC (mmol/l)**	4.87 (4.16, 5.50)	4.90 (4.10, 5.75)	5.07 (4.19, 5.86)
**LDL (mmol/l)**	3.20 (2.70, 3.76)	3.18 (2.60, 3.76)	3.01 (2.28, 3.68)
**HDL (mmol/l)**	1.20 (1.03, 1.40)	1.19 (1.03, 1.33)	1.29 (1.09, 1.53)
ALT (u/l)	20.20 (14.83, 28.08)	21.45 (15.25, 29.20)	19.70 (15.70, 26.64)
**AST (u/l)**	20.40 (17.00, 24.78)	20.00 (17.63, 25.45)	18.00 (15.30, 23.35)
**SCr (μmol/l)**	64.90 (54.25, 75.73)	62.20 (52.73, 73.50)	58.50 (53.00, 74.34)
**BUN (μmol/l)**	5.40 (4.50, 6.43)	5.25 (4.34, 6.29)	5.60 (4.69, 6.85)

Values presented as n (%), median (interquartile range).

Hb, hemoglobin; TG, triglyceride; TC, total cholesterol; LDL, low-density lipoprotein cholesterol; HDL, high-density lipoprotein cholesterol; ALT, alanine aminotransferase; AST, aspartate transaminase; SCr, serum creatinine; BUN, blood urea nitrogen.

### Effect of RBC lifespan on AG, HbA1c and HGI in T2DM patients

**Figure 2:**
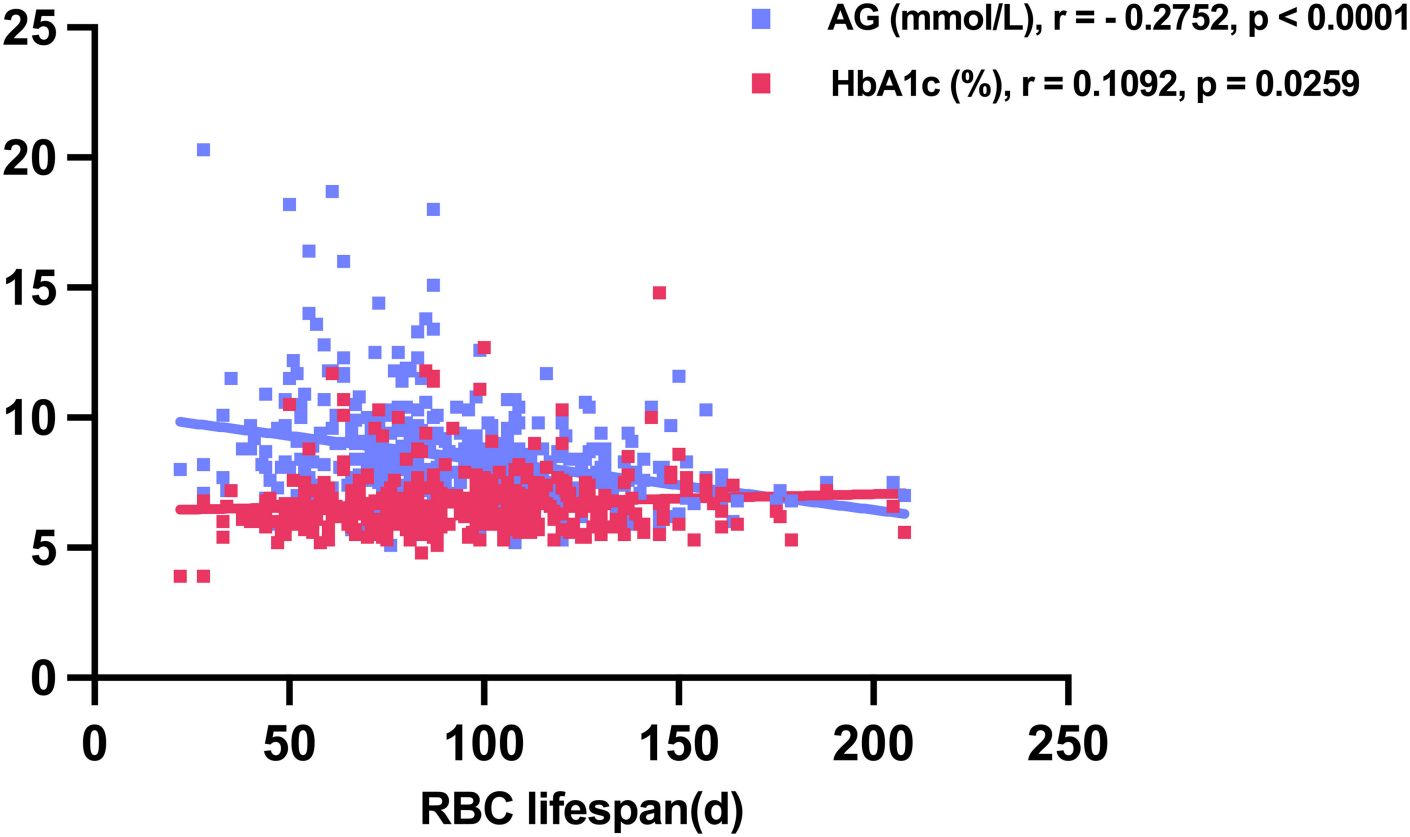
Average glucose (AG) and glycosylated hemoglobin (HbA1c) levels in relation to red blood cell (RBC) lifespan in construction cohort.

In the 416 T2DM patients with accessible AG and HbA1c data, a positive correlation was observed between HbA1c levels and RBC lifespan (r = 0.1092, p = 0.0259), while AG exhibited an inverse relationship with RBC lifespan (r = -0.2752, p < 0.0001) (**Figure 2**). The tested HbA1c may not be a precise indicator of the glycemic status of T2DM individuals and hyperglycemia may lead to a reduced RBC lifespan in this patient population.

**Figure 3:**
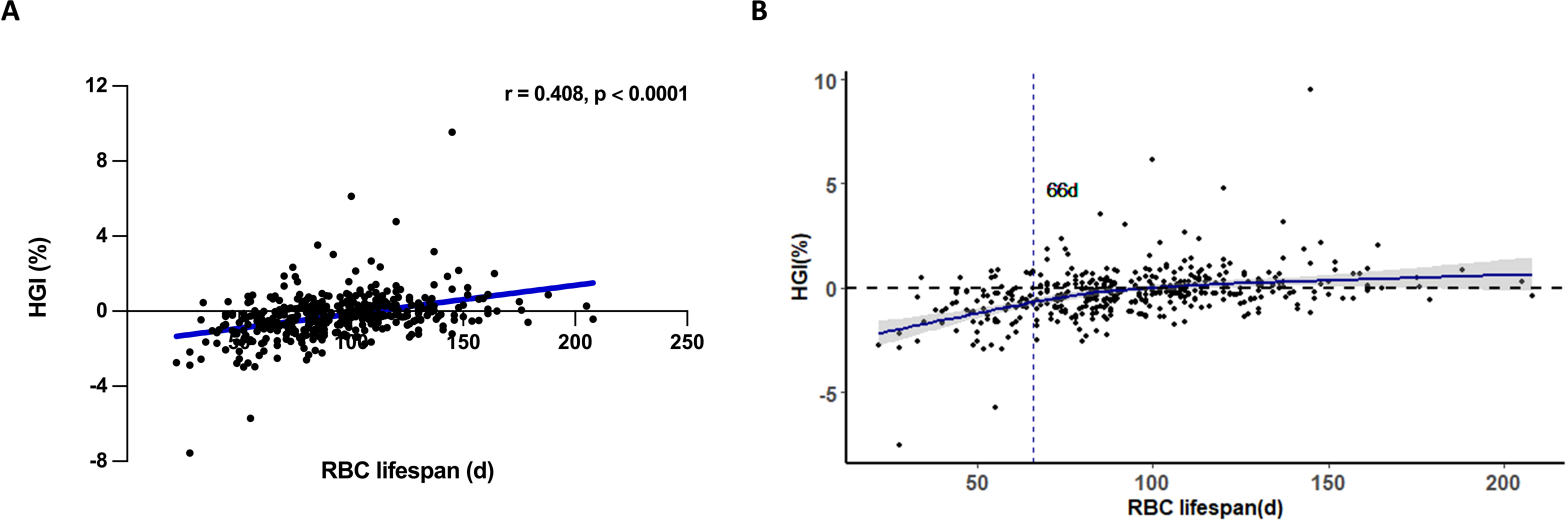
The impact of RBC lifespan upon hemoglobin variation glycation index (HGI) in type 2 diabetes mellitus (T2DM) patients. **(A)** Spearman correlation analysis of red blood cells (RBC) lifespan and HGI in T2DM patients; **(B)** Relationship between RBC lifespan and HGI restrictive spline regression fitting curve in T2DM patients. Etimated HbA1c (eHbA1c) = (average glucose (AG) + 2.5944)/1.5944. HGI = HbA1c - eHbA1c.

The HGI is a critical parameter for determining the deviation of HbA1c measurement values from estimated HbA1c values (17). The translation of AG to eHbA1c based on the formula: eHbA1c = (AG + 2.5944)/1.5944 (16). We examined the correlation between RBC lifespan and HGI within our construction cohort (**Figures 3A, B**). Consistent with results published before, a diminished RBC lifespan could cause an underestimation of HbA1c values among T2DM patients (12). At the same time, we observed a curve inflection point at RBC lifespan of 66 days (**Figure 3B**). Previous research has conventionally considered the RBC lifespan range from 90 to 120 days (18). Our study categorized construction group patients into three groups based on RBC lifespan: ≤ 66 days, between 67 and 89 days, and ≥ 90 days. No significant disparities in age, diabetes duration, liver function, and blood lipids were observed across the whole construction cohort and the three subgroups (**Table 2**). A shorter RBC lifespan could substantially elevate serum creatinine levels (p = 0.0128) and blood urea nitrogen (p = 0.0363) in patients, even when such levels were within the normal range. However, the reduction in RBC lifespan may pose a potential threat to renal function in patients with T2DM, which should not be overlooked.

**Table 2:** General clinical data of type 2 diabetes patients of different red blood cell (RBC) lifespan.

	Total	RBC lifespan ≤ 66d	67 ≤ RBC lifespan ≤ 89d	RBC lifespan ≥ 90d	P
**N (male/female)**	416 (218/198)	80 (51/29)	138 (73/65)	198 (94/104)	0.1085
**Age(year)**	58 (48, 65)	57 (44, 65)	57 (48, 64)	58 (49, 65)	0.5946
**Duration** **(year)**	7 (2, 12)	7 (2, 12)	7 (3, 12)	6 (3, 13)	0.9703
**TG** **(mmol/l)**	1.49 (1.12, 2.20)	1.40 (1.06, 2.00)	1.43 (1.10, 2.32)	1.65 (1.20, 2.20)	0.3600
**TC** **(mmol/l)**	4.87 (4.16, 5.50)	4.53 (3.80, 5.70)	4.84 (4.32, 5.38)	5.00 (4.39, 5.67)	0.0898
**LDL** **(mmol/l)**	3.20 (2.70, 3.76)	3.06 (2.35, 3.80)	3.21 (2.78, 3.73)	3.30 (2.82, 3.89)	0.1310
**HDL** **(mmol/l)**	1.20 (1.03, 1.40)	1.12 (0.99, 1.35)	1.26 (1.02, 1.41)	1.20 (1.05, 1.41)	0.2135
**ALT** **(U/L)**	20.20 (14.83, 28.08)	19.95 (13.73, 23.80)	19.40 (15.00, 28.25)	21.45 (14.90, 30.93)	0.2078
**AST** **(U/L)**	20.40 (17.00, 24.78)	19.40 (16.25, 23.15)	20.15 (17.00, 24.30)	21.40 (17.58, 26.53)	0.0869
**SCr** **(μmol/l)**	64.90 (54.25, 75.73)	68.95 (54.30, 78.85)	67.90 (58.43, 77.15)	60.10 (52.28, 73.30)	0.0128
**BUN** **(μmol/l)**	5.40 (4.50, 6.43)	5.61 (4.70, 6.68)	5.50 (4.57, 6.79)	5.14 (4.20, 6.15)	0.0363

Values presented as n (%), median (interquartile range). Patients were divided into three groups according to RBC lifespan: RBC lifespan ≤ 66 days group, 67 ≤ RBC lifespan ≤ 89 days group and RBC lifespan ≥ 90 days group.

Hb, hemoglobin; TG, triglyceride; TC, total cholesterol; LDL, low-density lipoprotein cholesterol; HDL, high-density lipoprotein cholesterol; ALT, alanine aminotransferase; AST, aspartate transaminase; SCr, serum creatinine; BUN, blood urea nitrogen.

### Underestimated HbA1c levels in diverse RBC lifespan groups

**Figure 4:**
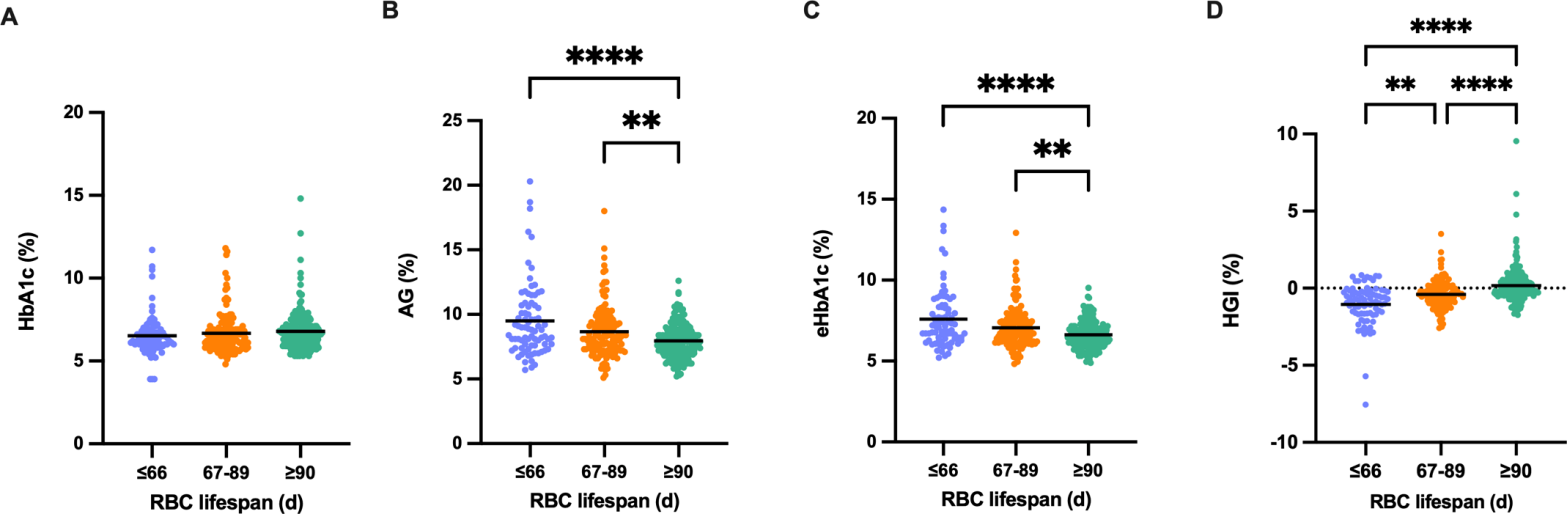
Underestimated glycosylated hemoglobin (HbA1c) levels in diverse red blood cells (RBC) lifespan groups. **(A-D)**. Comparison of HbA1c, average glucose (AG), estimated HbA1c (eHbA1c), and hemoglobin variation glycation index (HGI) in the different RBC lifespan groups. eHbA1c = (AG + 2.5944)/1.5944. HGI = HbA1c - eHbA1c. (**p < 0.01 ****p < 0.0001).

To confirm that a shorter RBC lifespan results in an underestimation of the HbA1c measurement value, we assessed the extent of underestimation in HbA1c measurements relative to different RBC lifespans. No significant variation was detected in HbA1c detection values among the three groups (**Figure 4A**); however, the AG and eHbA1c levels in groups with an RBC lifespan of less than 90 days were notably higher compared to those with a normal RBC lifespan (**Figures 4B, C**). The median (IQR) HGI of the three groups (**Figure 4D**) were -0.855 (-1.640, -0.238), -0.415 (-0.963, 0.133), and -0.020 (-0.423, 0.523), respectively. Consequently, the reduction in RBC lifespan could lead to a significant underestimation of blood glucose levels in T2DM patients, particularly in those with an RBC lifespan of ≤ 66 days.

### Underestimation of HbA1c values increased the risk of chronic complications in T2DM patients

**Figure 5:**
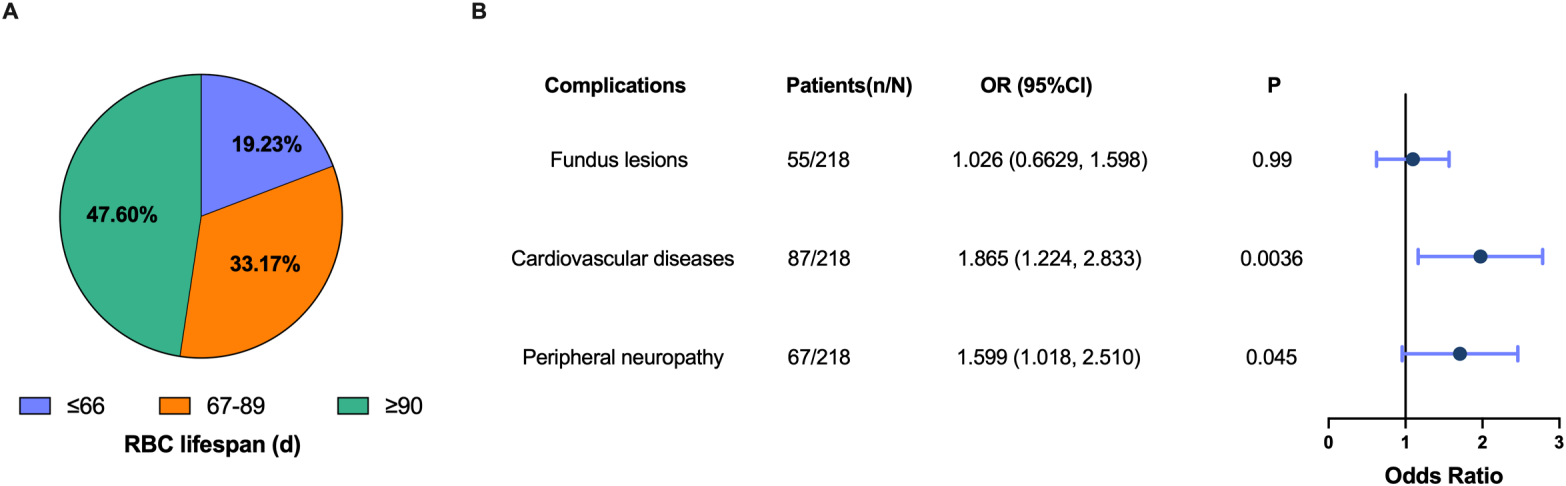
Underestimation of glycosylated hemoglobin (HbA1c) values increased the risk of chronic complications in type 2 diabetes mellitus (T2DM) patients. **(A)** Proportion of patients in the different red blood cells (RBC) lifespan groups; **(B)** Forest plot of the association between a shorter RBC lifespan (RBC lifespan < 90 d) and the risk of fundus lesions, cardiovascular disease and peripheral neurology. Dots depicts the point estimate. Horizontal bars depicts 95% confidence interval (CI). OR, odds ratio.

We established that HbA1c measurement values were subject to varying degrees of underestimation in patients with a RBC lifespan of less than 90 days. The distribution of patients across the groups with RBC lifespan ≤ 66 days, 67 to 89 days, and ≥ 90 days was 19.23%, 33.17%, and 47.60%, respectively (**Figure 5A**). A diminished RBC lifespan can result in inadequate glycemic control among individuals with T2DM, potentially elevating the risk for developing chronic complications. When the RBC lifespan was less than 90 days, there was a notably elevated risk for cardiovascular disease (OR 1.865, 95% CI 1.224 - 2.833) and peripheral neuropathy (OR 1.599, 95% CI 1.018 - 2.510) (**Figure 5B**). These results suggested that half (52.40%) of T2DM patients with their HbA1c test values underestimated in varying degrees, and it is imperative to rectify the influence of a shorter RBC lifespan on HbA1c test values to mitigate the risk of chronic complications.

### Modulation of the impact of shorter RBC lifespan upon HbA1c test values

**Figure 6:**
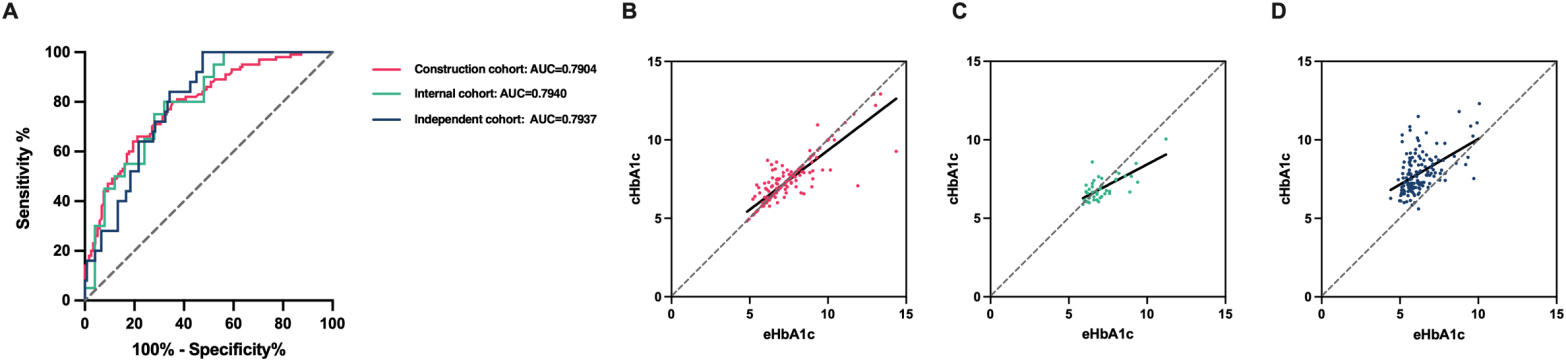
The corrected HbA1c models’ predictive performance and calibration in the different cohorts. **(A)** Diagnostic performance of the corrected glycosylated hemoglobin (cHbA1c) to detect poor management of blood glucose (estimated HbA1c (eHbA1c) > 7%) of type 2 diabetes mellitus (T2DM) patients in construction cohort, internal cohort and independent cohort. **(B-D)** Assessing calibration in construction cohort, internal cohort and independent cohort.

Based on the linear relationship between HbA1c and eHbA1c, and the different influence laws in RBC lifespan ≤ 66 day group and 67 ≤ RBC lifespan ≤ 89 days, we employed a multivariate linear regression model to formulate mathematical expressions that adjust for the impact of shorter RBC lifespan on HbA1c measurement values. The model incorporated RBC lifespan and measured HbA1c values as independent variables and the estimated HbA1c values as the dependent variable. P < 0.05 in the F tests, which meant that these regression formulas were meaningful. In the group with RBC lifespan ≤ 66 days, HbA1c(c) = -0.05629×RBC lifespan + 1.127×HbA1c + 3.178 (R = 0.7360). In the 67 ≤ RBC lifespan ≤ 89 days group, HbA1c(c) = -0.004772 × RBC lifespan + 0.7569 × HbA1c + 2.394 (R = 0.7344) may be used for correction. The corrected HbA1c models exhibited satisfactory predictive performance and calibration in the construction cohort (AUC 0.7904, 95% CI, 0.7313 - 0.8494), internal cohort (AUC 0.7940, 95% CI, 0.6646 - 0.9234) and independent cohort (AUC 0.7937, 95% CI, 0.7160 - 0.8714) (**Figures 6A–D**).

## Discussion

HbA1c serves as a critical metric for guiding clinicians in adjusting glucose-lowering therapies (2), and the accuracy with which it reflects the actual blood glucose levels in T2DM ndividuals is of profound clinical importance for glycemic management and the prevention of diabetes-related complications (3–5). Our study assessed the effects of variability in RBC lifespan on HbA1c measurement values, clarifying the necessity and significance of correcting this impact in glycemic control. Additionally, we established a mathematical formula to adjust for the influence of RBC lifespan on HbA1c measurement values. We provided accurate information about blood glucose levels in T2DM patients, thereby furthering the advanced management of blood glucose and diabetes-related chronic complications for clinical practice.

To ascertain the impact of RBC lifespan on T2DM patients’ HbA1c and to correct and verify this influence, we incorporated a construction cohort, an internal validation cohort, and an independent test cohort, all of which underwent a standardized HbA1c, AG, and biochemical indices detection protocol. The findings revealed substantial variability in RBC lifespan among T2DM patients. We observed the different correlations between AG, HbA1c and the RBC lifespan, suggesting that measured HbA1c might not accurately represent patients’ glycemic status, and hyperglycemia could contribute to a shortened RBC lifespan in T2DM individuals.

The linear association between RBC lifespan and HGI, which reflected the degree of HbA1c detection value deviating from eHbA1c, strongly indicated that the RBC lifespan variability is a significant cause of large HGI. Research has confirmed that the HGI, or glycosylation gap (Ggap)—an alternative measure to evaluate the discrepancy between HbA1c measurement values and AG—is an independent risk element for microvascular complications in T2DM patients, including diabetic nephropathy (12), diabetic retinopathy (19, 20), and macrovascular diseases (13, 14, 21). The presence of a significant HGI may result in HbA1c measurements that fail to accurately represent the true blood glucose levels of patients, leading to chronic suboptimal glycemic control. Consequently, identifying diabetic patients with large HGI is crucial for diminishing the risk of diabetes-related complications and enhancing their overall prognosis. Our results strongly suggested that RBC lifespan is a suitable parameter to assess HGI. Some studies have also reported that the characteristics of RBC lifespan themselves influence the HGI, including the survival time of RBC, the glycosylation rate of Hb, and genetic factors (16, 22). Malka reported that the variation in the average RBC age among individuals accounts for all the glucose-independent variability in HbA1c (23). Although this study had a robust design, its sample size was very small. Our study with a large sample also supported the conclusion that a shorter RBC lifespan may be the primary driver of HGI in diabetic individuals.

In our study, there were half of T2DM patients with a shorter RBC lifespan, which could substantially raise the risk of cardiovascular disease and peripheral neurology. This result may partly explain those clinical cases with suitable HbA1c levels who suffered from severe diabetic-related chronic complications. These cases may have reached chronic hyperglycemic status. Our study also strongly suggested that in the management of blood glucose levels in T2DM patients, it is essential to adjust for the impact of a shorter RBC lifespan on HbA1c test values to ascertain the actual blood glucose levels and to mitigate the risk of chronic complications. Consequently, a pilot study is warranted to elucidate the causal link between a shortened RBC lifespan and diabetes-related complications.

To accurately gauge whether T2DM patients with a shorter RBC lifespan have achieved the desired blood glucose levels, it is imperative to correct for the influence of RBC lifespan on HbA1c values. Utilizing the linear relationship between estimated HbA1c value and measured HbA1c, and considering the distinct effects observed in patients with an RBC lifespan of ≤ 66 days and those with 67 to 89 days, we employed a multivariate linear regression model to establish mathematical formulas that adjust for the impact of a shorter RBC lifespan on HbA1c measurement values. In patients with RBC lifespan ≤ 66 days, HbA1c(c) = -0.05629×RBC lifespan + 1.127×HbA1c + 3.178 (R = 0.7360). So, for patients with RBC lifespan less than 66 days, this formula helps us obtain a more trustable HbA1c value. In patients with an RBC lifespan of 67 ≤ RBC lifespan ≤ 89 days, HbA1c(c) = -0.004772 × RBC lifespan + 0.7569 × HbA1c + 2.394 (R = 0.7344) may be used for correction. The corrected HbA1c models exhibited satisfactory predictive performance and calibration in the construction cohort, internal cohort and independent cohort. In clinical practice, to precisely assess the blood glucose levels of T2DM individuals, both the tested HbA1c and the values of RBC lifespan are requisite. When a patient’s RBC lifespan is shorter than 90 days, their true HbA1c should be calculated using the aforementioned formulas. These formulas will provide valuable clinical insights to more accurately evaluate patients’ blood glucose levels and guide treatment decisions.

Our study had notable strengths. We enrolled a large and multi-center study to investigate the impact of variability in RBC lifespan on the HbA1cmeasurement value, clarifying the need and importance of correcting this influence in the management of blood glucose. We established the mathematic formulas to rectify the influence of RBC lifespan on HbA1c measurements and validated them across various population cohorts. The correction formula offers a more precise evaluation of glycemic control, particularly in patients with shortened RBC lifespan. This is crucial as standard HbA1c measurements may underestimate blood glucose levels in such cases, potentially leading to inadequate treatment. By providing a more accurate reflection of a patient’s true glycemic status, the formula enables clinicians to tailor treatment plans more effectively. This can result in more appropriate adjustments to diabetes medications and lifestyle interventions. Additionally, the corrected HbA1c values can improve the assessment of a patient’s risk for diabetes-related complications, allowing for better preventive measures and more timely interventions.

However, some questions still remain to be answered: 1) How often do we need to test RBC lifespan in patients with T2DM? 2) Do we need to avoid the menstrual period of women patients when testing their RBC lifespan? We acknowledge that our study has several limitations that need to be addressed in future research. Specifically, we did not account for potential confounding factors such as interindividual differences in hemoglobin glycation rates, anemia, and chronic kidney disease, that may affect the accuracy of HbA1c measurements. Future research should aim to incorporate these variables into more comprehensive models to further enhance the precision of glycemic assessment. Additionally, the study was conducted in Tianjin and the sample size was relatively small, which may limit the generalizability of our results and the statistical power of our analysis. Meanwhile, the specific characteristics of our cohorts may limit the direct applicability of these formulas to other populations. Further validation is necessary to confirm the effectiveness of our correction formulas in different patient populations with varying ethnic backgrounds, different stages of diabetes, and those with comorbid conditions.

In summary, the variation in RBC lifespan among T2DM patients is a crucial factor contributing to the discrepancy between measured and estimated HbA1c levels, known as HGI. A substantial proportion of diabetic individuals with a reduced RBC lifespan exhibited significantly underestimated HbA1c measurement values. Correcting the HbA1c measurements accurately in T2DM patients with a shorter RBC lifespan is essential. This correction is crucial for optimizing blood glucose management and for enhancing the outcomes related to chronic complications of diabetes. This approach will ultimately contribute to the advancement of clinical practices in diabetes care.

## Data Availability

The raw data supporting the conclusions of this article will be made available by the authors, without undue reservation.
