# Supplementary material for: Rectify the impact of shorter red blood cell lifespan upon HbA1c detection values in T2DM patients: modeling and internal-external verification
Source: Front Endocrinol (Lausanne). 2025 Apr 15;16:1500660. doi: 10.3389/fendo.2025.1500660 (PMC12037378; doi:10.3389/fendo.2025.1500660)
Supplement: Supplementary file 1 [file DataSheet1.pdf]

| RBCs | sex | Age | AG | HbA1c | Hb | course | HGI | ehba1c | TG |
|---|---|---|---|---|---|---|---|---|---|
| 134 | 2 | 55 | 6.5 | 5.8 | 138 | 12 | 0.10 | 5.70 | 2.58 |
| 22 | 1 | 42 | 8 | 3.9 | 170 | 3 | -2.74 | 6.64 | 2.24 |
| 28 | 1 | 33 | 8.2 | 3.9 | 132 | 9 | -2.87 | 6.77 | 1.38 |
| 28 | 1 | 40 | 7.1 | 3.9 | 173 | 3 | -2.18 | 6.08 | 1.13 |
| 34 | 1 | 56 | 7.2 | 6.6 | 151 | 11 | 0.46 | 6.14 | 1.97 |
| 38 | 1 | 65 | 8.8 | 6.1 | 158 | 10 | -1.05 | 7.15 | 1.4 |
| 40 | 1 | 65 | 8.8 | 6 | 140 | 12 | -1.15 | 7.15 | 1.36 |
| 40 | 1 | 59 | 9.7 | 6 | 172 | 4 | -1.71 | 7.71 | 6.79 |
| 41 | 1 | 58 | 9.2 | 6.4 | 170 | 2 | -1.00 | 7.40 | 2.34 |
| 43 | 1 | 42 | 8.2 | 6 | 144 | 8 | -0.77 | 6.77 | 0.9 |
| 44 | 1 | 49 | 10.9 | 6.4 | 188 | 3 | -2.06 | 8.46 | 2.28 |
| 44 | 1 | 38 | 8.1 | 6 | 137 | 1 | -0.71 | 6.71 | 1.25 |
| 45 | 1 | 43 | 7.6 | 6.9 | 71 | 20 | 0.51 | 6.39 | 2.02 |
| 47 | 1 | 58 | 7.3 | 6 | 148 | 2 | -0.21 | 6.21 | 2.18 |
| 49 | 1 | 70 | 8.3 | 5.7 | 162 | 2 | -1.13 | 6.83 | 0.72 |
| 49 | 1 | 73 | 10.7 | 6.7 | 164 | 5 | -1.64 | 8.34 | 0.78 |
| 50 | 1 | 47 | 8.1 | 6.1 | 169 | 3 | -0.61 | 6.71 | 1.09 |
| 52 | 1 | 63 | 9.1 | 6.3 | 161 | 10 | -1.03 | 7.33 | 0.75 |
| 52 | 1 | 55 | 7 | 5.9 | 89 | 2 | -0.12 | 6.02 | 3.9 |
| 52 | 1 | 38 | 7.6 | 6 | 149 | 7 | -0.39 | 6.39 | 0.73 |
| 54 | 1 | 50 | 8.1 | 7.5 | 156 | 21 | 0.79 | 6.71 | 1.03 |
| 54 | 1 | 71 | 8.1 | 6 | 164 | 2 | -0.71 | 6.71 | 1.83 |
| 54 | 1 | 63 | 10.9 | 5.7 | 145 | 16 | -2.76 | 8.46 | 2.1 |
| 55 | 1 | 65 | 9.1 | 5.8 | 163 | 15 | -1.53 | 7.33 | 3 |
| 55 | 1 | 68 | 7.7 | 6.2 | 161 | 0 | -0.26 | 6.46 | 1.3 |
| 55 | 1 | 58 | 6.3 | 6.1 | 166 | 8 | 0.52 | 5.58 | 1.9 |
| 55 | 1 | 60 | 14 | 8.8 | 133 | 10 | -1.61 | 10.41 | 0.78 |
| 57 | 1 | 33 | 7.1 | 6.1 | 145 | 16 | 0.02 | 6.08 | 2.46 |
| 58 | 1 | 47 | 7.4 | 5.2 | 166 | 2 | -1.07 | 6.27 | 0.8 |
| 58 | 1 | 43 | 9.4 | 7 | 170 | 1 | -0.52 | 7.52 | 0.8 |
| 59 | 1 | 60 | 12.8 | 7.3 | 164 | 0 | -2.36 | 9.66 | 0.51 |
| 59 | 1 | 55 | 8.2 | 6.5 | 149 | 3 | -0.27 | 6.77 | 1.06 |
| 60 | 1 | 62 | 6.8 | 5.3 | 153 | 2 | -0.59 | 5.89 | 1.2 |
| 60 | 1 | 69 | 7 | 5.6 | 152 | 2 | -0.42 | 6.02 | 2.2 |
| 61 | 1 | 62 | 9.6 | 6.7 | 132 | 20 | -0.95 | 7.65 | 0.98 |
| 61 | 1 | 44 | 18.7 | 11.7 | 155 | 11 | -1.66 | 13.36 | 1.6 |
| 61 | 1 | 72 | 11.8 | 6.9 | 143 | 3 | -2.13 | 9.03 | 1.44 |
| 62 | 1 | 52 | 9 | 6.6 | 160 | 5 | -0.67 | 7.27 | 1.37 |
| 62 | 1 | 52 | 10.1 | 6.5 | 158 | 2 | -1.46 | 7.96 | 1.49 |
| 63 | 1 | 65 | 8.1 | 6.2 | 157 | 6 | -0.51 | 6.71 | 0.98 |
| 63 | 1 | 46 | 9 | 6.4 | 176 | 12 | -0.87 | 7.27 | 0.95 |
| 64 | 1 | 57 | 11.7 | 8 | 171 | 1 | -0.97 | 8.97 | 1.38 |
| 65 | 1 | 36 | 7.4 | 5.9 | 129 | 24 | -0.37 | 6.27 | 1.73 |
| 66 | 1 | 52 | 9.2 | 6.7 | 150 | 5 | -0.70 | 7.40 | 3.34 |
| 66 | 1 | 35 | 6.7 | 5.8 | 142 | 20 | -0.03 | 5.83 | 1.13 |
| 66 | 1 | 35 | 7.2 | 6.1 | 151 | 1 | -0.04 | 6.14 | 1.43 |
| 67 | 1 | 52 | 9.1 | 5.5 | 148 | 8 | -1.83 | 7.33 | 0.65 |
| 67 | 1 | 67 | 9 | 5.7 | 164 | 12 | -1.57 | 7.27 | 0.76 |
| 67 | 1 | 61 | 9.9 | 6.8 | 165 | 18 | -1.04 | 7.84 | 0.92 |
| 67 | 1 | 39 | 10.5 | 5.7 | 150 | 18 | -2.51 | 8.21 | 1.47 |
| 68 | 1 | 55 | 7.8 | 6.6 | 154 | 10 | 0.08 | 6.52 | 3.92 |
| 70 | 1 | 59 | 7.6 | 6.6 | 140 | 3 | 0.21 | 6.39 | 1.2 |
| 70 | 1 | 53 | 8.4 | 6.7 | 139 | 20 | -0.20 | 6.90 | 1.56 |
| 70 | 1 | 34 | 8.3 | 6.1 | 149 | 4 | -0.73 | 6.83 | 0.66 |
| 71 | 1 | 68 | 9.1 | 6.1 | 164 | 26 | -1.23 | 7.33 | 0.34 |
| 72 | 1 | 60 | 7.7 | 5.5 | 160 | 8 | -0.96 | 6.46 | 1.16 |
| 72 | 1 | 41 | 8 | 6.1 | 150 | 10 | -0.54 | 6.64 | 1.67 |
| 72 | 1 | 64 | 12.5 | 9.6 | 113 | 12 | 0.13 | 9.47 | 3.58 |
| 73 | 1 | 34 | 14.4 | 10.3 | 144 | 1 | -0.36 | 10.66 | 1.97 |
| 73 | 1 | 58 | 7.5 | 5.7 | 148 | 3 | -0.63 | 6.33 | 3.01 |
| 73 | 1 | 46 | 6.2 | 5.7 | 176 | 0 | 0.18 | 5.52 | 0.78 |
| 74 | 1 | 47 | 9.3 | 6.9 | 167 | 6 | -0.56 | 7.46 | 0.83 |

| | | | | | | | | | |
|---|---|---|---|---|---|---|---|---|---|
| 74 | 1 | 35 | 7.2 | 5.8 | 157 | 3 | -0.34 | 6.14 | 0.9 |
| 74 | 1 | 67 | 7.8 | 5.4 | 149 | 8 | -1.12 | 6.52 | 0.81 |
| 75 | 1 | 83 | 10.1 | 6.9 | 159 | 10 | -1.06 | 7.96 | 4 |
| 75 | 1 | 69 | 10.3 | 6.9 | 149 | 30 | -1.19 | 8.09 | 1.3 |
| 75 | 1 | 64 | 7.3 | 5.8 | 161 | 25 | -0.41 | 6.21 | 1.09 |
| 75 | 1 | 66 | 6.6 | 5.3 | 182 | 7 | -0.47 | 5.77 | 1.35 |
| 75 | 1 | 36 | 7.2 | 5.8 | 147 | 11 | -0.34 | 6.14 | 1.22 |
| 75 | 1 | 61 | 7.2 | 5.8 | 146 | 2 | -0.34 | 6.14 | 0.57 |
| 75 | 1 | 38 | 8.2 | 6.4 | 138 | 10 | -0.37 | 6.77 | 1.12 |
| 76 | 1 | 57 | 8.5 | 7.1 | 158 | 8 | 0.14 | 6.96 | 1.68 |
| 76 | 1 | 48 | 6.1 | 6.1 | 153 | 2 | 0.65 | 5.45 | 1 |
| 77 | 1 | 46 | 8.1 | 7.6 | 162 | 3 | 0.89 | 6.71 | 1.18 |
| 78 | 1 | 61 | 10.4 | 6.8 | 168 | 3 | -1.35 | 8.15 | 1.44 |
| 78 | 1 | 64 | 10.1 | 6.5 | 158 | 1 | -1.46 | 7.96 | 2.81 |
| 78 | 1 | 60 | 12.5 | 10 | 146 | 3 | 0.53 | 9.47 | 0.77 |
| 79 | 1 | 64 | 8.9 | 6.2 | 166 | 6 | -1.01 | 7.21 | 1.86 |
| 80 | 1 | 41 | 9.8 | 6.5 | 150 | 1 | -1.27 | 7.77 | 2.76 |
| 80 | 1 | 67 | 6.8 | 6.5 | 150 | 10 | 0.61 | 5.89 | 1.1 |
| 80 | 1 | 57 | 8 | 6.6 | 159 | 12 | -0.04 | 6.64 | 1.1 |
| 80 | 1 | 70 | 11.9 | 6.5 | 164 | 2 | -2.59 | 9.09 | 2.3 |
| 80 | 1 | 51 | 9.6 | 6.7 | 159 | 11 | -0.95 | 7.65 | 3.07 |
| 81 | 1 | 64 | 7.1 | 5.6 | 138 | 14 | -0.48 | 6.08 | 1.06 |
| 81 | 1 | 59 | 9.1 | 7.3 | 157 | 6 | -0.03 | 7.33 | 0.77 |
| 81 | 1 | 53 | 8.3 | 6.8 | 151 | 4 | -0.03 | 6.83 | 2.16 |
| 82 | 1 | 29 | 7.3 | 5.6 | 157 | 20 | -0.61 | 6.21 | 2.22 |
| 82 | 1 | 29 | 6.8 | 5.5 | 148 | 5 | -0.39 | 5.89 | 2.01 |
| 83 | 1 | 57 | 9.7 | 6.2 | 183 | 3 | -1.51 | 7.71 | 2.39 |
| 83 | 1 | 60 | 7.1 | 5.6 | 157 | 1 | -0.48 | 6.08 | 5.6 |
| 83 | 1 | 43 | 7.2 | 6.5 | 159 | 9 | 0.36 | 6.14 | 2.49 |
| 83 | 1 | 47 | 8.3 | 5.8 | 153 | 2 | -1.03 | 6.83 | 0.61 |
| 83 | 1 | 41 | 12.3 | 8.8 | 166 | 7 | -0.54 | 9.34 | 2.27 |
| 83 | 1 | 55 | 13.3 | 8.7 | 145 | 9 | -1.27 | 9.97 | 1.28 |
| 83 | 1 | 48 | 10.3 | 7.7 | 169 | 12 | -0.39 | 8.09 | 5.7 |
| 83 | 1 | 64 | 9 | 7.2 | 171 | 2 | -0.07 | 7.27 | 3.3 |
| 84 | 1 | 66 | 9.3 | 6.5 | 134 | 17 | -0.96 | 7.46 | 1.6 |
| 84 | 1 | 50 | 9.2 | 6.2 | 153 | 7 | -1.20 | 7.40 | 1.5 |
| 84 | 1 | 31 | 9.5 | 5.5 | 150 | 20 | -2.09 | 7.59 | 1.8 |
| 84 | 1 | 49 | 7.7 | 6.1 | 151 | 2 | -0.36 | 6.46 | 2.2 |
| 85 | 1 | 35 | 13.8 | 9.4 | 165 | 2 | -0.88 | 10.28 | 0.91 |
| 86 | 1 | 70 | 9 | 6.3 | 147 | 20 | -0.97 | 7.27 | 1.5 |
| 86 | 1 | 75 | 8 | 6 | 172 | 1 | -0.64 | 6.64 | 1.3 |
| 86 | 1 | 59 | 8.8 | 6.6 | 171 | 3 | -0.55 | 7.15 | 9.51 |
| 87 | 1 | 27 | 13.4 | 7.8 | 140 | 10 | -2.23 | 10.03 | 3.07 |
| 87 | 1 | 22 | 15.1 | 11.6 | 163 | 18 | 0.50 | 11.10 | 1.24 |
| 87 | 1 | 39 | 10 | 7.8 | 167 | 10 | -0.10 | 7.90 | 1.08 |
| 87 | 1 | 40 | 18 | 11.4 | 165 | 11 | -1.52 | 12.92 | 1.2 |
| 87 | 1 | 48 | 7.3 | 5.4 | 161 | 9 | -0.81 | 6.21 | 0.6 |
| 88 | 1 | 74 | 7.1 | 5.1 | 124 | 15 | -0.98 | 6.08 | 3.05 |
| 88 | 1 | 66 | 6.9 | 6 | 146 | 13 | 0.05 | 5.95 | 0.88 |
| 89 | 1 | 42 | 7.2 | 6.4 | 164 | 3 | 0.26 | 6.14 | 3.11 |
| 89 | 1 | 60 | 7.4 | 7.1 | 148 | 18 | 0.83 | 6.27 | 2.9 |
| 89 | 1 | 41 | 7.5 | 5.8 | 150 | 8 | -0.53 | 6.33 | 1.8 |
| 89 | 1 | 51 | 8.1 | 6.9 | 168 | 20 | 0.19 | 6.71 | 1.4 |
| 90 | 1 | 32 | 9.1 | 7 | 159 | 9 | -0.33 | 7.33 | 1.48 |
| 94 | 1 | 48 | 7.5 | 6.6 | 163 | 16 | 0.27 | 6.33 | 1.62 |
| 95 | 1 | 45 | 8.8 | 7.9 | 164 | 3 | 0.75 | 7.15 | 1.15 |
| 95 | 1 | 68 | 8.6 | 6.3 | 143 | 10 | -0.72 | 7.02 | 2.1 |
| 95 | 1 | 40 | 8.4 | 6.9 | 146 | 20 | 0.00 | 6.90 | 1.5 |
| 96 | 1 | 67 | 9 | 6.9 | 161 | 18 | -0.37 | 7.27 | 1.96 |
| 96 | 1 | 61 | 6.4 | 5.4 | 159 | 2 | -0.24 | 5.64 | 6.08 |
| 96 | 1 | 70 | 10.3 | 6.8 | 146 | 20 | -1.29 | 8.09 | 0.6 |
| 97 | 1 | 42 | 9.3 | 6.9 | 167 | 5 | -0.56 | 7.46 | 0.8 |
| 98 | 1 | 61 | 8.4 | 6.8 | 136 | 4 | -0.10 | 6.90 | 1.12 |

| | | | | | | | | | |
|---|---|---|---|---|---|---|---|---|---|
| 98 | 1 | 44 | 10.8 | 7.1 | 158 | 18 | -1.30 | 8.40 | 1.26 |
| 98 | 1 | 70 | 9 | 7.8 | 158 | 12 | 0.53 | 7.27 | 1.2 |
| 98 | 1 | 72 | 8.8 | 6.8 | 141 | 2 | -0.35 | 7.15 | 1.15 |
| 98 | 1 | 69 | 7.4 | 6.8 | 122 | 8 | 0.53 | 6.27 | 1.05 |
| 100 | 1 | 48 | 6.6 | 5.9 | 134 | 16 | 0.13 | 5.77 | 1.43 |
| 100 | 1 | 53 | 7 | 5.9 | 139 | 3 | -0.12 | 6.02 | 1.9 |
| 101 | 1 | 61 | 8.2 | 6.5 | 151 | 6 | -0.27 | 6.77 | 1.6 |
| 101 | 1 | 43 | 7.8 | 7 | 142 | 2 | 0.48 | 6.52 | 2.38 |
| 51 | 1 | 45 | 12.2 | 7.6 | 125 | 7 | -1.68 | 9.28 | 1.5 |
| 102 | 1 | 58 | 7.7 | 6.3 | 120 | 13 | -0.16 | 6.46 | 2.08 |
| 102 | 1 | 57 | 8.2 | 7.6 | 152 | 2 | 0.83 | 6.77 | 1.38 |
| 104 | 1 | 52 | 7.1 | 7.2 | 153 | 3 | 1.12 | 6.08 | 1.81 |
| 104 | 1 | 67 | 8.4 | 7.9 | 157 | 10 | 1.00 | 6.90 | 1.33 |
| 104 | 1 | 58 | 7.5 | 6.1 | 140 | 12 | -0.23 | 6.33 | 1.77 |
| 104 | 1 | 58 | 7.8 | 6.3 | 125 | 10 | -0.22 | 6.52 | 1.87 |
| 105 | 1 | 65 | 6.8 | 5.7 | 122 | 4 | -0.19 | 5.89 | 2.36 |
| 105 | 1 | 61 | 7 | 5.3 | 147 | 2 | -0.72 | 6.02 | 1.76 |
| 105 | 1 | 54 | 8.7 | 7 | 123 | 16 | -0.08 | 7.08 | 1.33 |
| 106 | 1 | 68 | 10.7 | 6.6 | 130 | 5 | -1.74 | 8.34 | 1.38 |
| 106 | 1 | 37 | 8.3 | 6.8 | 101 | 14 | -0.03 | 6.83 | 1.4 |
| 106 | 1 | 57 | 8.7 | 7.5 | 149 | 20 | 0.42 | 7.08 | 1.48 |
| 106 | 1 | 61 | 9.1 | 6.3 | 83 | 2 | -1.03 | 7.33 | 0.94 |
| 57 | 1 | 55 | 13.6 | 7.2 | 128 | 20 | -2.96 | 10.16 | 3.12 |
| 108 | 1 | 71 | 10 | 8 | 123 | 5 | 0.10 | 7.90 | 1.35 |
| 108 | 1 | 35 | 7.6 | 6.7 | 116 | 6 | 0.31 | 6.39 | 1.82 |
| 108 | 1 | 42 | 7 | 5.9 | 138 | 5 | -0.12 | 6.02 | 0.88 |
| 108 | 1 | 61 | 8.6 | 6.6 | 132 | 7 | -0.42 | 7.02 | 1.09 |
| 109 | 1 | 69 | 10.4 | 6.5 | 126 | 5 | -1.65 | 8.15 | 1.14 |
| 109 | 1 | 59 | 7.9 | 6.7 | 148 | 10 | 0.12 | 6.58 | 1.91 |
| 109 | 1 | 48 | 9.8 | 7.1 | 122 | 21 | -0.67 | 7.77 | 1.2 |
| 110 | 1 | 58 | 6.5 | 6 | 144 | 4 | 0.30 | 5.70 | 2.15 |
| 110 | 1 | 66 | 7.2 | 5.8 | 136 | 3 | -0.34 | 6.14 | 5.47 |
| 111 | 1 | 67 | 8.1 | 7.7 | 140 | 5 | 0.99 | 6.71 | 1.78 |
| 111 | 1 | 38 | 7.9 | 8 | 81 | 12 | 1.42 | 6.58 | 1.89 |
| 112 | 1 | 46 | 7.3 | 6.7 | 116 | 13 | 0.49 | 6.21 | 0.94 |
| 113 | 1 | 62 | 6.9 | 6.3 | 159 | 6 | 0.35 | 5.95 | 1.4 |
| 113 | 1 | 65 | 8 | 9 | 121 | 2 | 2.36 | 6.64 | 0.99 |
| 114 | 1 | 65 | 7.1 | 5.9 | 138 | 1 | -0.18 | 6.08 | 1.35 |
| 114 | 1 | 59 | 9.8 | 7.5 | 130 | 2 | -0.27 | 7.77 | 1.8 |
| 114 | 1 | 43 | 7.4 | 7.1 | 165 | 10 | 0.83 | 6.27 | 6.7 |
| 115 | 1 | 74 | 7.9 | 7.1 | 134 | 13 | 0.52 | 6.58 | 1.69 |
| 115 | 1 | 51 | 7.8 | 6.8 | 135 | 19 | 0.28 | 6.52 | 1.3 |
| 117 | 1 | 58 | 8.3 | 6.6 | 127 | 20 | -0.23 | 6.83 | 1.8 |
| 118 | 1 | 65 | 7.2 | 6.4 | 130 | 5 | 0.26 | 6.14 | 1.32 |
| 118 | 1 | 55 | 6.3 | 5.3 | 159 | 14 | -0.28 | 5.58 | 2.38 |
| 118 | 1 | 41 | 6.8 | 6.1 | 117 | 1 | 0.21 | 5.89 | 1.21 |
| 120 | 1 | 73 | 10 | 7.3 | 140 | 20 | -0.60 | 7.90 | 1.93 |
| 120 | 1 | 50 | 6.5 | 5.7 | 126 | 30 | -0.00 | 5.70 | 1.4 |
| 120 | 1 | 58 | 9.8 | 7.6 | 98 | 1 | -0.17 | 7.77 | 1.67 |
| 121 | 1 | 72 | 7.7 | 7.2 | 146 | 0 | 0.74 | 6.46 | 1.3 |
| 121 | 1 | 48 | 9.3 | 7.5 | 162 | 1 | 0.04 | 7.46 | 1.85 |
| 122 | 1 | 68 | 6.9 | 7.1 | 100 | 9 | 1.15 | 5.95 | 2.62 |
| 124 | 1 | 49 | 8.4 | 6.6 | 144 | 6 | -0.30 | 6.90 | 1.9 |
| 125 | 1 | 67 | 8 | 6.4 | 151 | 16 | -0.24 | 6.64 | 1.4 |
| 127 | 1 | 69 | 10.4 | 6.9 | 89 | 19 | -1.25 | 8.15 | 1.06 |
| 128 | 1 | 48 | 7.5 | 5.8 | 131 | 5 | -0.53 | 6.33 | 1.94 |
| 130 | 1 | 48 | 8.1 | 5.7 | 109 | 2 | -1.01 | 6.71 | 1.71 |
| 130 | 1 | 58 | 7.5 | 6.2 | 163 | 2 | -0.13 | 6.33 | 2.32 |
| 130 | 1 | 47 | 9.4 | 6.3 | 140 | 18 | -1.22 | 7.52 | 0.84 |
| 131 | 1 | 54 | 8.1 | 7 | 160 | 3 | 0.29 | 6.71 | 1.7 |
| 131 | 1 | 72 | 8.4 | 7 | 148 | 1 | 0.10 | 6.90 | 1.05 |
| 133 | 1 | 59 | 7.1 | 6.8 | 124 | 1 | 0.72 | 6.08 | 0.8 |
| 135 | 1 | 35 | 6.2 | 6 | 139 | 19 | 0.48 | 5.52 | 1.3 |

| | | | | | | | | | |
|---|---|---|---|---|---|---|---|---|---|
| 136 | 1 | 23 | 6.2 | 5.5 | 124 | 15 | -0.02 | 5.52 | 1.41 |
| 137 | 1 | 59 | 9.4 | 7.8 | 133 | 2 | 0.28 | 7.52 | 3.45 |
| 141 | 1 | 71 | 8.4 | 5.9 | 137 | 7 | -1.00 | 6.90 | 1.6 |
| 145 | 1 | 58 | 8.1 | 5.5 | 158 | 10 | -1.21 | 6.71 | 2.26 |
| 148 | 1 | 66 | 6.2 | 7.7 | 134 | 4 | 2.18 | 5.52 | 2.1 |
| 150 | 1 | 59 | 11.6 | 8.6 | 144 | 2 | -0.30 | 8.90 | 3.24 |
| 154 | 1 | 75 | 6.7 | 5.3 | 139 | 1 | -0.53 | 5.83 | 1.95 |
| 157 | 1 | 72 | 10.3 | 7.6 | 141 | 3 | -0.49 | 8.09 | 1.4 |
| 161 | 1 | 25 | 6.5 | 5.8 | 152 | 13 | 0.10 | 5.70 | 1.1 |
| 175 | 1 | 57 | 6.8 | 6.4 | 121 | 22 | 0.51 | 5.89 | 2.1 |
| 205 | 1 | 37 | 7.5 | 6.6 | 110 | 1 | 0.27 | 6.33 | 1.21 |
| 208 | 1 | 65 | 7 | 5.6 | 127 | 2 | -0.42 | 6.02 | 0.46 |
| 28 | 2 | 28 | 20.3 | 6.8 | 136 | 16 | -7.56 | 14.36 | 0.78 |
| 33 | 2 | 66 | 7.7 | 6 | 130 | 1 | -0.46 | 6.46 | 1.6 |
| 35 | 2 | 29 | 11.5 | 7.2 | 138 | 12 | -1.64 | 8.84 | 0.86 |
| 44 | 2 | 57 | 8.7 | 6.8 | 151 | 10 | -0.28 | 7.08 | 1.54 |
| 47 | 2 | 42 | 9.6 | 6.6 | 120 | 10 | -1.05 | 7.65 | 1.54 |
| 48 | 2 | 72 | 8.1 | 5.5 | 135 | 15 | -1.21 | 6.71 | 3.06 |
| 49 | 2 | 72 | 10.6 | 5.5 | 140 | 5 | -2.78 | 8.28 | 1.64 |
| 49 | 2 | 66 | 9.7 | 6.5 | 114 | 7 | -1.21 | 7.71 | 4.8 |
| 50 | 2 | 67 | 18.2 | 10.5 | 135 | 16 | -2.54 | 13.04 | 1.3 |
| 50 | 2 | 61 | 11.5 | 6.7 | 139 | 14 | -2.14 | 8.84 | 0.8 |
| 53 | 2 | 78 | 10.5 | 6.7 | 130 | 1 | -1.51 | 8.21 | 0.9 |
| 53 | 2 | 47 | 8.4 | 6.5 | 141 | 13 | -0.40 | 6.90 | 2.67 |
| 53 | 2 | 67 | 10 | 6.6 | 112 | 19 | -1.30 | 7.90 | 1.4 |
| 55 | 2 | 58 | 16.4 | 6.2 | 146 | 1 | -5.71 | 11.91 | 1.4 |
| 56 | 2 | 52 | 8.3 | 6.6 | 157 | 14 | -0.23 | 6.83 | 1.72 |
| 56 | 2 | 43 | 8.9 | 5.6 | 136 | 10 | -1.61 | 7.21 | 1.2 |
| 59 | 2 | 59 | 10.7 | 7.5 | 117 | 7 | -0.84 | 8.34 | 1.16 |
| 60 | 2 | 72 | 11.8 | 7.3 | 162 | 23 | -1.73 | 9.03 | 1.06 |
| 60 | 2 | 56 | 6.9 | 5.8 | 156 | 12 | -0.15 | 5.95 | 0.91 |
| 61 | 2 | 57 | 7.3 | 6.2 | 138 | 7 | -0.01 | 6.21 | 1.33 |
| 64 | 2 | 33 | 12.3 | 10.1 | 130 | 4 | 0.76 | 9.34 | 2.93 |
| 64 | 2 | 65 | 16 | 10.7 | 126 | 18 | -0.96 | 11.66 | 4.04 |
| 64 | 2 | 65 | 11.6 | 8.3 | 135 | 20 | -0.60 | 8.90 | 2.55 |
| 65 | 2 | 57 | 6.4 | 6.3 | 123 | 2 | 0.66 | 5.64 | 1.74 |
| 66 | 2 | 66 | 9.6 | 6.9 | 154 | 1 | -0.75 | 7.65 | 1.8 |
| 67 | 2 | 57 | 8.4 | 6.3 | 141 | 0 | -0.60 | 6.90 | 1.04 |
| 68 | 2 | 61 | 7 | 6.6 | 136 | 3 | 0.58 | 6.02 | 1.39 |
| 68 | 2 | 75 | 10.8 | 7.6 | 133 | 14 | -0.80 | 8.40 | 1.95 |
| 68 | 2 | 44 | 9.1 | 7.1 | 161 | 44 | -0.23 | 7.33 | 1.48 |
| 70 | 2 | 67 | 8.2 | 6.7 | 147 | 14 | -0.07 | 6.77 | 0.98 |
| 70 | 2 | 57 | 5.8 | 5.4 | 102 | 6 | 0.14 | 5.26 | 2.72 |
| 71 | 2 | 51 | 8.5 | 5.7 | 172 | 0 | -1.26 | 6.96 | 2.49 |
| 72 | 2 | 61 | 9.7 | 6.8 | 144 | 1 | -0.91 | 7.71 | 2.91 |
| 73 | 2 | 57 | 9.7 | 6.1 | 135 | 20 | -1.61 | 7.71 | 2.54 |
| 73 | 2 | 67 | 9.1 | 6.8 | 149 | 1 | -0.53 | 7.33 | 1.63 |
| 73 | 2 | 66 | 8.1 | 6.1 | 144 | 3 | -0.61 | 6.71 | 3.46 |
| 73 | 2 | 55 | 9.8 | 6.8 | 148 | 3 | -0.97 | 7.77 | 1.26 |
| 74 | 2 | 56 | 8.5 | 9.3 | 134 | 5 | 2.34 | 6.96 | 1.88 |
| 74 | 2 | 68 | 9 | 6.9 | 135 | 0 | -0.37 | 7.27 | 1.39 |
| 74 | 2 | 49 | 8.1 | 5.8 | 142 | 12 | -0.91 | 6.71 | 1.24 |
| 75 | 2 | 61 | 6.6 | 5.9 | 149 | 2 | 0.13 | 5.77 | 1.12 |
| 75 | 2 | 64 | 8.6 | 6.6 | 132 | 12 | -0.42 | 7.02 | 1.2 |
| 76 | 2 | 63 | 9.4 | 7.3 | 153 | 4 | -0.22 | 7.52 | 1.33 |
| 77 | 2 | 53 | 11.8 | 7.6 | 139 | 5 | -1.43 | 9.03 | 1.73 |
| 78 | 2 | 65 | 9.5 | 5.9 | 129 | 6 | -1.69 | 7.59 | 1.1 |
| 78 | 2 | 68 | 8.1 | 6.9 | 130 | 8 | 0.19 | 6.71 | 0.81 |
| 79 | 2 | 57 | 8.4 | 5.9 | 134 | 2 | -1.00 | 6.90 | 3.2 |
| 79 | 2 | 65 | 11.4 | 6.8 | 136 | 5 | -1.98 | 8.78 | 2.63 |
| 80 | 2 | 60 | 10.4 | 8.4 | 167 | 4 | 0.25 | 8.15 | 4.74 |
| 81 | 2 | 65 | 8.3 | 6.8 | 149 | 10 | -0.03 | 6.83 | 1.4 |
| 81 | 2 | 62 | 7 | 5.3 | 131 | 1 | -0.72 | 6.02 | 1.09 |

| | | | | | | | | | |
|---|---|---|---|---|---|---|---|---|---|
| 81 | 2 | 59 | 8.5 | 6.5 | 126 | 1 | -0.46 | 6.96 | 1.01 |
| 81 | 2 | 59 | 7.6 | 6 | 147 | 15 | -0.39 | 6.39 | 1.29 |
| 81 | 2 | 68 | 7.7 | 5.6 | 128 | 1 | -0.86 | 6.46 | 0.7 |
| 81 | 2 | 70 | 11.8 | 6.7 | 133 | 25 | -2.33 | 9.03 | 2.53 |
| 82 | 2 | 54 | 8.4 | 6.8 | 119 | 3 | -0.10 | 6.90 | 2.62 |
| 82 | 2 | 62 | 6.1 | 5.7 | 132 | 20 | 0.25 | 5.45 | 1.3 |
| 83 | 2 | 55 | 8 | 6.2 | 124 | 13 | -0.44 | 6.64 | 1.37 |
| 83 | 2 | 42 | 7.2 | 6.8 | 154 | 29 | 0.66 | 6.14 | 1.43 |
| 83 | 2 | 53 | 9.3 | 7 | 149 | 4 | -0.46 | 7.46 | 0.96 |
| 83 | 2 | 29 | 7.6 | 6.2 | 154 | 3 | -0.19 | 6.39 | 1.89 |
| 83 | 2 | 66 | 7.8 | 7.2 | 148 | 0 | 0.68 | 6.52 | 2.5 |
| 84 | 2 | 22 | 5.7 | 4.8 | 118 | 30 | -0.40 | 5.20 | 0.59 |
| 84 | 2 | 79 | 11.5 | 8.7 | 136 | 13 | -0.14 | 8.84 | 1 |
| 84 | 2 | 58 | 8.8 | 6.6 | 133 | 20 | -0.55 | 7.15 | 1.84 |
| 84 | 2 | 54 | 7.2 | 6.3 | 146 | 40 | 0.16 | 6.14 | 1.29 |
| 85 | 2 | 54 | 6 | 5.8 | 138 | 12 | 0.41 | 5.39 | 1.62 |
| 85 | 2 | 71 | 10.6 | 11.8 | 129 | 1 | 3.52 | 8.28 | 1.54 |
| 86 | 2 | 62 | 9 | 5.9 | 134 | 2 | -1.37 | 7.27 | 0.9 |
| 86 | 2 | 63 | 8.8 | 7.3 | 114 | 12 | 0.15 | 7.15 | 1.1 |
| 86 | 2 | 66 | 7.3 | 6.3 | 138 | 11 | 0.09 | 6.21 | 1.27 |
| 86 | 2 | 55 | 7.4 | 6.6 | 123 | 14 | 0.33 | 6.27 | 2.5 |
| 87 | 2 | 48 | 6.8 | 5.4 | 78 | 30 | -0.49 | 5.89 | 3.66 |
| 87 | 2 | 49 | 7.7 | 7.4 | 148 | 23 | 0.94 | 6.46 | 3.21 |
| 87 | 2 | 66 | 6.6 | 6.1 | 122 | 5 | 0.33 | 5.77 | 1.98 |
| 88 | 2 | 68 | 10.1 | 6.8 | 159 | 2 | -1.16 | 7.96 | 2.6 |
| 88 | 2 | 49 | 9.2 | 6.1 | 144 | 1 | -1.30 | 7.40 | 1.3 |
| 88 | 2 | 58 | 6.9 | 5.3 | 133 | 2 | -0.65 | 5.95 | 1.47 |
| 88 | 2 | 74 | 7.2 | 5.9 | 126 | 1 | -0.24 | 6.14 | 1.19 |
| 88 | 2 | 53 | 8.1 | 6.1 | 143 | 1 | -0.61 | 6.71 | 1.1 |
| 88 | 2 | 55 | 9.4 | 7.3 | 147 | 28 | -0.22 | 7.52 | 1.48 |
| 88 | 2 | 63 | 7.7 | 5.9 | 152 | 12 | -0.56 | 6.46 | 1.12 |
| 89 | 2 | 67 | 8.3 | 6.2 | 130 | 8 | -0.63 | 6.83 | 1 |
| 89 | 2 | 61 | 7.9 | 6.2 | 126 | 1 | -0.38 | 6.58 | 1.8 |
| 90 | 2 | 58 | 7.6 | 5.9 | 124 | 1 | -0.49 | 6.39 | 1.44 |
| 91 | 2 | 62 | 8.7 | 7 | 160 | 4 | -0.08 | 7.08 | 2.33 |
| 91 | 2 | 75 | 7.1 | 5.9 | 138 | 5 | -0.18 | 6.08 | 1.16 |
| 92 | 2 | 54 | 7.9 | 9.6 | 140 | 6 | 3.02 | 6.58 | 0.98 |
| 93 | 2 | 74 | 10.4 | 7 | 86 | 1 | -1.15 | 8.15 | 0.58 |
| 94 | 2 | 30 | 8.6 | 6.3 | 130 | 15 | -0.72 | 7.02 | 2.56 |
| 94 | 2 | 40 | 9.4 | 6.4 | 153 | 5 | -1.12 | 7.52 | 1.8 |
| 95 | 2 | 70 | 8.1 | 6.8 | 142 | 8 | 0.09 | 6.71 | 0.8 |
| 95 | 2 | 55 | 8.3 | 6.2 | 139 | 14 | -0.63 | 6.83 | 1.63 |
| 96 | 2 | 25 | 5.5 | 5.5 | 119 | 17 | 0.42 | 5.08 | 1.42 |
| 97 | 2 | 52 | 8.3 | 5.6 | 136 | 0 | -1.23 | 6.83 | 1.5 |
| 98 | 2 | 50 | 9.2 | 6.8 | 125 | 1 | -0.60 | 7.40 | 2.74 |
| 98 | 2 | 35 | 9.2 | 6.7 | 128 | 2 | -0.70 | 7.40 | 0.79 |
| 98 | 2 | 49 | 9.3 | 6.1 | 121 | 7 | -1.36 | 7.46 | 1.75 |
| 99 | 2 | 75 | 8.5 | 6.1 | 153 | 3 | -0.86 | 6.96 | 1.8 |
| 99 | 2 | 62 | 12.6 | 11.1 | 144 | 12 | 1.57 | 9.53 | 1.2 |
| 100 | 2 | 65 | 8.8 | 7.7 | 132 | 15 | 0.55 | 7.15 | 0.8 |
| 100 | 2 | 62 | 8.8 | 6.8 | 114 | 27 | -0.35 | 7.15 | 1.34 |
| 100 | 2 | 66 | 7.9 | 12.7 | 136 | 2 | 6.12 | 6.58 | 1.9 |
| 101 | 2 | 55 | 9.8 | 7.5 | 137 | 10 | -0.27 | 7.77 | 0.95 |
| 102 | 2 | 57 | 7.6 | 6.1 | 98 | 4 | -0.29 | 6.39 | 2.94 |
| 102 | 2 | 69 | 8.5 | 9.1 | 109 | 12 | 2.14 | 6.96 | 2.7 |
| 102 | 2 | 51 | 9.7 | 6.7 | 131 | 4 | -1.01 | 7.71 | 2.91 |
| 102 | 2 | 64 | 9.5 | 6.2 | 142 | 2 | -1.39 | 7.59 | 0.95 |
| 103 | 2 | 69 | 9 | 5.9 | 115 | 22 | -1.37 | 7.27 | 1.9 |
| 103 | 2 | 45 | 7.9 | 6.9 | 130 | 2 | 0.32 | 6.58 | 1.48 |
| 104 | 2 | 66 | 7.2 | 7.1 | 141 | 2 | 0.96 | 6.14 | 1.3 |
| 105 | 2 | 40 | 6.3 | 5.7 | 145 | 10 | 0.12 | 5.58 | 1.2 |
| 105 | 2 | 66 | 7.8 | 6.1 | 105 | 16 | -0.42 | 6.52 | 2.1 |
| 106 | 2 | 51 | 8.4 | 7.3 | 115 | 2 | 0.40 | 6.90 | 1.55 |

| | | | | | | | | | |
|---|---|---|---|---|---|---|---|---|---|
| 106 | 2 | 60 | 9.6 | 7.4 | 146 | 5 | -0.25 | 7.65 | 2.61 |
| 106 | 2 | 75 | 8.3 | 6.7 | 154 | 3 | -0.13 | 6.83 | 1.17 |
| 106 | 2 | 66 | 6.9 | 6.7 | 108 | 20 | 0.75 | 5.95 | 2.26 |
| 107 | 2 | 42 | 7.4 | 6 | 138 | 1 | -0.27 | 6.27 | 2.31 |
| 107 | 2 | 52 | 6.2 | 5.7 | 149 | 3 | 0.18 | 5.52 | 2.2 |
| 108 | 2 | 51 | 9.6 | 7.2 | 134 | 9 | -0.45 | 7.65 | 1.98 |
| 108 | 2 | 71 | 8.4 | 7.2 | 137 | 7 | 0.30 | 6.90 | 0.8 |
| 108 | 2 | 62 | 10.7 | 8 | 126 | 3 | -0.34 | 8.34 | 3.4 |
| 109 | 2 | 74 | 8.8 | 6.7 | 138 | 0 | -0.45 | 7.15 | 1.95 |
| 109 | 2 | 56 | 5.9 | 5.6 | 129 | 24 | 0.27 | 5.33 | 3.1 |
| 109 | 2 | 57 | 7.2 | 6 | 118 | 25 | -0.14 | 6.14 | 1.68 |
| 110 | 2 | 55 | 8.2 | 6.5 | 83 | 9 | -0.27 | 6.77 | 0.87 |
| 111 | 2 | 74 | 8.4 | 6 | 148 | 18 | -0.90 | 6.90 | 1 |
| 111 | 2 | 57 | 7.6 | 7 | 143 | 12 | 0.61 | 6.39 | 1 |
| 112 | 2 | 57 | 6.5 | 5.9 | 132 | 4 | 0.20 | 5.70 | 3.4 |
| 112 | 2 | 63 | 7.3 | 5.6 | 131 | 1 | -0.61 | 6.21 | 2.31 |
| 112 | 2 | 56 | 8.4 | 6.4 | 137 | 2 | -0.50 | 6.90 | 1.1 |
| 112 | 2 | 71 | 8.6 | 6.9 | 143 | 9 | -0.12 | 7.02 | 1 |
| 52 | 2 | 31 | 11.7 | 6 | 144 | 10 | -2.97 | 8.97 | 1.64 |
| 113 | 2 | 63 | 7.3 | 6.7 | 146 | 1 | 0.49 | 6.21 | 2.7 |
| 114 | 2 | 49 | 8 | 5.7 | 136 | 3 | -0.94 | 6.64 | 2.2 |
| 115 | 2 | 65 | 8.8 | 6.9 | 149 | 4 | -0.25 | 7.15 | 2.2 |
| 116 | 2 | 59 | 11.7 | 8.1 | 152 | 7 | -0.87 | 8.97 | 1.07 |
| 116 | 2 | 55 | 7.5 | 7.4 | 148 | 2 | 1.07 | 6.33 | 1.4 |
| 116 | 2 | 50 | 7.4 | 6.7 | 158 | 18 | 0.43 | 6.27 | 2.23 |
| 119 | 2 | 61 | 8.7 | 7.7 | 160 | 2 | 0.62 | 7.08 | 2.03 |
| 122 | 2 | 58 | 6.8 | 5.6 | 157 | 7 | -0.29 | 5.89 | 1.8 |
| 122 | 2 | 64 | 8.4 | 6.6 | 162 | 5 | -0.30 | 6.90 | 2.08 |
| 123 | 2 | 63 | 7.3 | 5.9 | 181 | 3 | -0.31 | 6.21 | 0.59 |
| 125 | 2 | 65 | 6.2 | 5.5 | 140 | 15 | -0.02 | 5.52 | 3.12 |
| 125 | 2 | 62 | 7.2 | 5.4 | 104 | 22 | -0.74 | 6.14 | 1.27 |
| 126 | 2 | 61 | 7.1 | 5.4 | 156 | 3 | -0.68 | 6.08 | 2.53 |
| 126 | 2 | 69 | 8.4 | 7.5 | 134 | 20 | 0.60 | 6.90 | 2.1 |
| 126 | 2 | 70 | 10.6 | 6.9 | 159 | 4 | -1.38 | 8.28 | 1.74 |
| 126 | 2 | 45 | 7.7 | 7.2 | 155 | 10 | 0.74 | 6.46 | 1.27 |
| 127 | 2 | 53 | 8.7 | 7.4 | 159 | 6 | 0.32 | 7.08 | 6.06 |
| 128 | 2 | 44 | 8.8 | 6.6 | 143 | 1 | -0.55 | 7.15 | 3 |
| 129 | 2 | 70 | 7.9 | 6.1 | 152 | 11 | -0.48 | 6.58 | 2.5 |
| 130 | 2 | 61 | 6 | 5.7 | 128 | 10 | 0.31 | 5.39 | 1 |
| 130 | 2 | 61 | 6 | 5.5 | 150 | 4 | 0.11 | 5.39 | 0.88 |
| 130 | 2 | 61 | 6.1 | 5.9 | 133 | 11 | 0.45 | 5.45 | 4.02 |
| 33 | 2 | 63 | 10.1 | 5.4 | 162 | 5 | -2.56 | 7.96 | 1.3 |
| 131 | 2 | 60 | 7.4 | 5.8 | 170 | 20 | -0.47 | 6.27 | 2.95 |
| 131 | 2 | 64 | 8.8 | 6.7 | 169 | 12 | -0.45 | 7.15 | 4.07 |
| 134 | 2 | 54 | 7.5 | 6.1 | 159 | 6 | -0.23 | 6.33 | 0.77 |
| 136 | 2 | 45 | 7.4 | 6.5 | 137 | 1 | 0.23 | 6.27 | 1.2 |
| 136 | 2 | 55 | 8.3 | 7.5 | 159 | 13 | 0.67 | 6.83 | 0.9 |
| 137 | 2 | 62 | 7.7 | 6.5 | 160 | 2 | 0.04 | 6.46 | 2.39 |
| 138 | 2 | 65 | 9.1 | 6.9 | 161 | 6 | -0.43 | 7.33 | 1.41 |
| 139 | 2 | 58 | 7.9 | 6.3 | 159 | 10 | -0.28 | 6.58 | 2.63 |
| 140 | 2 | 62 | 7.1 | 5.9 | 155 | 1 | -0.18 | 6.08 | 1 |
| 141 | 2 | 71 | 7.6 | 5.6 | 111 | 15 | -0.79 | 6.39 | 1.06 |
| 71 | 2 | 64 | 9.5 | 6.1 | 145 | 5 | -1.49 | 7.59 | 1.43 |
| 143 | 2 | 61 | 10.4 | 10 | 156 | 0 | 1.85 | 8.15 | 1.4 |
| 146 | 2 | 56 | 7.9 | 6.1 | 132 | 14 | -0.48 | 6.58 | 1.54 |
| 146 | 2 | 61 | 8.1 | 6.5 | 154 | 3 | -0.21 | 6.71 | 3.3 |
| 148 | 2 | 60 | 9.7 | 7.9 | 165 | 10 | 0.19 | 7.71 | 2.79 |
| 150 | 2 | 37 | 6.3 | 5.9 | 102 | 2 | 0.32 | 5.58 | 1.58 |
| 152 | 2 | 66 | 6.9 | 7.2 | 176 | 7 | 1.25 | 5.95 | 1.92 |
| 152 | 2 | 70 | 8.3 | 7.7 | 118 | 13 | 0.87 | 6.83 | 1.2 |
| 157 | 2 | 41 | 7.7 | 7.1 | 140 | 0 | 0.64 | 6.46 | 1.87 |
| 159 | 2 | 50 | 7 | 6.7 | 161 | 6 | 0.68 | 6.02 | 1.82 |
| 161 | 2 | 59 | 7.3 | 7.1 | 139 | 18 | 0.89 | 6.21 | 3.82 |

| | | | | | | | | | |
|---|---|---|---|---|---|---|---|---|---|
| 161 | 2 | 41 | 7.8 | 6.4 | 156 | 0 | -0.12 | 6.52 | 3.5 |
| 164 | 2 | 62 | 6 | 7.4 | 116 | 30 | 2.01 | 5.39 | 0.72 |
| 165 | 2 | 47 | 6.8 | 5.9 | 170 | 5 | 0.01 | 5.89 | 1.9 |
| 176 | 2 | 53 | 7.2 | 6.2 | 158 | 15 | 0.06 | 6.14 | 1.1 |
| 179 | 2 | 49 | 6.8 | 5.3 | 160 | 3 | -0.59 | 5.89 | 2.35 |
| 188 | 2 | 66 | 7.5 | 7.2 | 166 | 8 | 0.87 | 6.33 | 2.21 |
| 120 | 1 | 60 | 10.1 | 9 | 155 | 9 | 1.04 | 7.96 | 0.53 |
| 137 | 1 | 43 | 5.9 | 8.5 | 134 | 12 | 3.17 | 5.33 | 1.92 |
| 80 | 2 | 42 | 6.6 | 5.7 | 145 | 7 | -0.07 | 5.77 | 2.08 |
| 56 | 1 | 55 | 6.1 | 6.3 | 132 | 12 | 0.85 | 5.45 | 0.96 |
| 145 | 2 | 53 | 6.2 | 6.7 | 169 | 1 | 1.18 | 5.52 | 1.09 |
| 120 | 2 | 52 | 5.3 | 6.3 | 179 | 3 | 1.35 | 4.95 | 3.67 |
| 54 | 1 | 61 | 8 | 7.1 | 177 | 1 | 0.46 | 6.64 | 2.67 |
| 103 | 1 | 73 | 7 | 6.1 | 159 | 3 | 0.08 | 6.02 | 1.82 |
| 108 | 2 | 58 | 5.2 | 5.8 | 150 | 2 | 0.91 | 4.89 | 1.49 |
| 113 | 1 | 33 | 8 | 7.4 | 158 | 9 | 0.76 | 6.64 | 0.96 |
| 98 | 2 | 54 | 7.7 | 7.4 | 154 | 5 | 0.94 | 6.46 | 2.6 |
| 44 | 1 | 32 | 6.9 | 5.8 | 164 | 8 | -0.15 | 5.95 | 1.14 |
| 70 | 2 | 33 | 7.7 | 7.8 | 174 | 6 | 1.34 | 6.46 | 5 |
| 88 | 1 | 54 | 7 | 6.3 | 159 | 10 | 0.28 | 6.02 | 2.09 |
| 109 | 1 | 41 | 6.2 | 8.2 | 142 | 15 | 2.68 | 5.52 | 2.88 |
| 99 | 1 | 43 | 5.4 | 5.3 | 172 | 5 | 0.29 | 5.01 | 1.74 |
| 120 | 1 | 34 | 6.2 | 10.3 | 129 | 7 | 4.78 | 5.52 | 1.82 |
| 75 | 1 | 50 | 5.8 | 7.1 | 146 | 7 | 1.84 | 5.26 | 1.38 |
| 78 | 1 | 50 | 7.4 | 6.6 | 134 | 1 | 0.33 | 6.27 | 1.43 |
| 145 | 1 | 39 | 5.8 | 14.8 | 153 | 3 | 9.54 | 5.26 | 2.18 |
| 99 | 1 | 40 | 6.2 | 6.4 | 140 | 9 | 0.88 | 5.52 | 1.2 |
| 81 | 2 | 60 | 5.3 | 5.7 | 158 | 13 | 0.75 | 4.95 | 1.41 |
| 109 | 1 | 36 | 5.9 | 6.6 | 146 | 10 | 1.27 | 5.33 | 1.06 |
| 90 | 2 | 60 | 9.2 | 8.2 | 168 | 5 | 0.80 | 7.40 | 3.16 |
| 120 | 1 | 50 | 8.5 | 7.3 | 134 | 10 | 0.34 | 6.96 | 1.44 |
| 70 | 2 | 55 | 10 | 7.7 | 147 | 7 | -0.20 | 7.90 | 2.49 |
| 66 | 2 | 66 | 7.7 | 7.2 | 151 | 6 | 0.74 | 6.46 | 1.61 |
| 70 | 2 | 43 | 6.7 | 7.7 | 142 | 5 | 1.87 | 5.83 | 1.72 |
| 76 | 1 | 70 | 5.1 | 6.4 | 131 | 9 | 1.57 | 4.83 | 1.05 |
| 47 | 2 | 59 | 5.9 | 5.2 | 135 | 1 | -0.13 | 5.33 | 1.32 |
| 66 | 1 | 68 | 5.7 | 6 | 152 | 5 | 0.80 | 5.20 | 1.23 |
| 137 | 2 | 42 | 7.6 | 7.8 | 136 | 14 | 1.41 | 6.39 | 1.04 |
| 98 | 2 | 42 | 7.7 | 6.4 | 133 | 3 | -0.06 | 6.46 | 1.37 |

| TC | HDL | LDL | ALT | AST | SCr | BUN | Cardiovascular disease | Fundus lesions | peripheral neuropathy |
|---|---|---|---|---|---|---|---|---|---|
| 3.92 | 0.91 | 2.61 | 13.7 | 21.5 | 256.6 | 22.01 | 0 | 0 | 1 |
| 7.58 | 0.84 | 4.57 | 54.8 | 43.2 | 77.6 | 8.38 | 1 | 1 | 1 |
| 6.44 | 1.09 | 4.17 | 44.9 | 45.8 | 64.6 | 7.3 | 1 | 1 | 1 |
| 6.79 | 0.85 | 4.74 | 20 | 17.8 | 87.3 | 6.08 | 1 | 1 | 1 |
| 5.83 | 0.95 | 3.29 | 30.8 | 17.8 | 63.9 | 3.7 | 1 | 0 | 0 |
| 7.7 | 1.3 | 5.6 | 23.1 | 14.8 | 61.2 | 5.6 | 1 | 0 | 1 |
| 5.83 | 1.05 | 4.2 | 13.7 | 15.1 | 66.8 | 6.55 | 1 | 0 | 0 |
| 6.5 | 0.99 | 5.07 | 20.5 | 12.7 | 69.3 | 5.99 | 1 | 1 | 0 |
| 6.25 | 0.71 | 4.73 | 20 | 22.4 | 85.2 | 4.71 | 1 | 1 | 0 |
| 3.28 | 1.3 | 1.8 | 17 | 20 | 78.7 | 5.5 | 0 | 0 | 0 |
| 6.96 | 1.22 | 4.53 | 12.7 | 25.1 | 69.5 | 6.84 | 1 | 1 | 1 |
| 3.92 | 0.89 | 2.77 | 10.2 | 14.9 | 67.2 | 4.25 | 0 | 0 | 0 |
| 6.91 | 0.92 | 4.15 | 42.9 | 40.2 | 62.4 | 5.71 | 1 | 0 | 0 |
| 3.82 | 1.2 | 1.89 | 16 | 19.1 | 61 | 4.25 | 0 | 0 | 0 |
| 5.38 | 0.99 | 3.25 | 21.4 | 15.2 | 71.8 | 5.42 | 1 | 0 | 0 |
| 3.73 | 1.04 | 2.49 | 8.6 | 16.4 | 99.2 | 6.17 | 0 | 0 | 1 |
| 4.43 | 1.38 | 2.76 | 22.6 | 19.3 | 65.3 | 6.39 | 0 | 0 | 0 |
| 4.16 | 1.6 | 2.43 | 7.9 | 21.8 | 84.1 | 8.34 | 0 | 0 | 1 |
| 4.92 | 1.15 | 3.08 | 13 | 22 | 78.5 | 5.3 | 1 | 0 | 0 |
| 4.59 | 1.33 | 3.08 | 11.2 | 12 | 70.5 | 7.5 | 0 | 0 | 0 |
| 5.66 | 0.94 | 4.43 | 55 | 50.1 | 67.4 | 5.85 | 1 | 0 | 0 |
| 3.97 | 0.9 | 2.81 | 18.9 | 19.3 | 74.5 | 6 | 0 | 0 | 0 |
| 6.2 | 1.1 | 4.7 | 42.9 | 40.2 | 58.2 | 5.61 | 1 | 1 | 1 |
| 4.4 | 0.9 | 2.8 | 18.5 | 25.8 | 116 | 6.25 | 0 | 1 | 1 |
| 3.16 | 1.04 | 1.99 | 23.5 | 19.2 | 114.2 | 11.67 | 0 | 0 | 0 |
| 5.6 | 1.2 | 3.9 | 19.9 | 21.4 | 74.8 | 5.4 | 0 | 0 | 0 |
| 3.23 | 0.91 | 2.08 | 22 | 16.9 | 97.3 | 7.22 | 0 | 0 | 1 |
| 5.71 | 0.91 | 4.04 | 6.1 | 10.9 | 70.9 | 3.99 | 0 | 0 | 0 |
| 4.3 | 1.6 | 2.1 | 27.9 | 32.3 | 88.2 | 8.61 | 1 | 0 | 0 |
| 3.6 | 1.3 | 2 | 13.2 | 21.5 | 170.4 | 11.96 | 0 | 0 | 0 |
| 4.85 | 1.23 | 3.32 | 22.6 | 23.2 | 65.7 | 3.71 | 1 | 0 | 1 |
| 6.41 | 1.51 | 4.71 | 41.6 | 40.4 | 86.3 | 6.96 | 0 | 0 | 0 |
| 3.4 | 1.1 | 2.2 | 20.7 | 21.4 | 74.1 | 5.95 | 0 | 0 | 0 |
| 4.1 | 1.07 | 2.85 | 36.2 | 27.3 | 74 | 8.26 | 1 | 0 | 0 |
| 5.07 | 1.09 | 3.74 | 10 | 18.1 | 73.6 | 3.92 | 0 | 0 | 0 |
| 6.3 | 1.5 | 4.2 | 17.7 | 19.9 | 86.6 | 6.2 | 0 | 0 | 0 |
| 3.37 | 0.98 | 2.16 | 13.2 | 15.9 | 68.3 | 4.7 | 0 | 0 | 1 |
| 3.46 | 1.17 | 2.08 | 15.9 | 16.4 | 365.8 | 17.42 | 0 | 0 | 0 |
| 2.11 | 0.78 | 1.15 | 18.8 | 15.8 | 478.7 | 20.76 | 0 | 1 | 0 |
| 3.07 | 1.22 | 1.57 | 21.1 | 24.7 | 103.2 | 6.68 | 0 | 0 | 0 |
| 2.54 | 0.95 | 1.39 | 16.8 | 19.6 | 86.4 | 6.1 | 1 | 0 | 0 |
| 3.59 | 1.48 | 1.9 | 15.5 | 20.7 | 67.2 | 4.44 | 0 | 0 | 0 |
| 4.73 | 1.1 | 3.34 | 28.4 | 18.8 | 68.6 | 5.45 | 0 | 0 | 0 |
| 5.23 | 1.11 | 3.54 | 15 | 14.9 | 92.3 | 7.1 | 1 | 0 | 0 |
| 3.82 | 1.09 | 2.6 | 18.8 | 14.4 | 72.8 | 5.44 | 1 | 0 | 1 |
| 3.8 | 0.89 | 2.74 | 20.3 | 20.1 | 78.9 | 5.35 | 0 | 0 | 0 |
| 4.39 | 0.93 | 2.98 | 14.1 | 11.9 | 58.9 | 6.17 | 1 | 1 | 1 |
| 5.38 | 1.29 | 3.79 | 17 | 17.3 | 74.1 | 6.74 | 1 | 1 | 1 |
| 4.3 | 1.7 | 2.5 | 11.9 | 24.4 | 75.3 | 5.05 | 0 | 0 | 0 |
| 5.15 | 1.02 | 3.78 | 50.5 | 34.5 | 72 | 6.94 | 1 | 1 | 1 |
| 5.47 | 1.02 | 3.76 | 35 | 24.3 | 67.5 | 4.38 | 1 | 0 | 0 |
| 4.7 | 1 | 3.2 | 23.6 | 26.9 | 90.7 | 6 | 0 | 0 | 0 |
| 4.15 | 0.78 | 2.98 | 20.2 | 18.3 | 110.7 | 6.03 | 0 | 0 | 0 |
| 3.38 | 1.13 | 2.18 | 19.4 | 19.8 | 54.4 | 3.9 | 1 | 0 | 0 |
| 3.16 | 1.61 | 1.51 | 19.4 | 19.7 | 79.2 | 8.2 | 1 | 0 | 1 |
| 4.43 | 1.21 | 3.02 | 29.6 | 20.8 | 74.3 | 5.1 | 0 | 0 | 0 |
| 4.17 | 0.96 | 2.7 | 10.6 | 15.2 | 72.3 | 5.66 | 0 | 0 | 0 |
| 7.55 | 1.34 | 5.46 | 23.3 | 17.8 | 67.8 | 5.33 | 0 | 0 | 0 |
| 4.71 | 1.33 | 2.85 | 16.2 | 18.4 | 71.3 | 5.43 | 0 | 0 | 0 |
| 3.43 | 0.88 | 2.35 | 11.8 | 12.3 | 62.5 | 7.99 | 0 | 0 | 0 |
| 4.75 | 1.53 | 2.78 | 19.3 | 20.3 | 66.6 | 6.49 | 0 | 0 | 0 |
| 4.97 | 1.36 | 3.31 | 19.4 | 26.9 | 82 | 10.61 | 0 | 0 | 0 |

| | | | | | | | | | |
|---|---|---|---|---|---|---|---|---|---|
| 4.37 | 1.23 | 3 | 15.6 | 19.8 | 66 | 7.41 | 0 | 0 | 0 |
| 4.92 | 1.41 | 3.28 | 24.8 | 17.6 | 72.8 | 5.15 | 0 | 1 | 0 |
| 5.2 | 0.9 | 3.6 | 9.5 | 16.7 | 97.5 | 5.8 | 1 | 0 | 0 |
| 5.6 | 1.4 | 3.9 | 34.1 | 24.5 | 59.8 | 5 | 0 | 0 | 1 |
| 4.83 | 1.6 | 2.99 | 15.9 | 22.2 | 68 | 5 | 1 | 1 | 0 |
| 5.62 | 1.54 | 3.67 | 13.5 | 15.4 | 64 | 4.3 | 0 | 0 | 0 |
| 4.93 | 1.49 | 2.85 | 13.7 | 17 | 80.5 | 5.71 | 1 | 0 | 1 |
| 4.11 | 1.58 | 2.36 | 18.3 | 17.8 | 86.9 | 9.5 | 0 | 0 | 0 |
| 3.32 | 1.13 | 2.06 | 15 | 14.6 | 153.6 | 8.07 | 1 | 0 | 0 |
| 5.51 | 1.02 | 3.78 | 17.8 | 15 | 93 | 5.63 | 0 | 0 | 0 |
| 4.65 | 1.4 | 2.86 | 11.8 | 23.7 | 103.4 | 7.54 | 0 | 0 | 0 |
| 4.87 | 1.26 | 3.36 | 13.6 | 13 | 60.9 | 6.3 | 0 | 0 | 0 |
| 4.76 | 1.32 | 3.22 | 14.7 | 15.5 | 57.9 | 4.57 | 0 | 0 | 0 |
| 5.06 | 1.16 | 3.61 | 57.3 | 31.8 | 105.2 | 8.58 | 1 | 0 | 0 |
| 4.32 | 1.06 | 3.06 | 20.9 | 15.5 | 82.7 | 7.93 | 0 | 0 | 0 |
| 5.97 | 1.15 | 4.43 | 21 | 22.5 | 74.9 | 3.9 | 0 | 0 | 0 |
| 5.05 | 1.08 | 3.53 | 28.4 | 20.1 | 77.6 | 5.91 | 0 | 0 | 0 |
| 3.1 | 1.4 | 1.4 | 16.7 | 16.5 | 58.9 | 4 | 0 | 0 | 0 |
| 5.2 | 1.2 | 4.1 | 17.7 | 22.9 | 58.9 | 4.52 | 0 | 1 | 0 |
| 3.2 | 0.8 | 2.1 | 8.6 | 20 | 46.6 | 5.7 | 1 | 0 | 0 |
| 7.2 | 1.49 | 4.98 | 14.1 | 15.9 | 197.9 | 11.75 | 1 | 1 | 1 |
| 4.36 | 1.15 | 3.05 | 20.4 | 17.3 | 60 | 6.5 | 1 | 1 | 1 |
| 3.43 | 0.89 | 2.38 | 20.5 | 17.7 | 65.6 | 5.75 | 1 | 1 | 1 |
| 4.35 | 0.78 | 3.1 | 20.9 | 17.8 | 96.7 | 4.31 | 0 | 0 | 0 |
| 4.74 | 0.96 | 3.07 | 22 | 22.9 | 49.6 | 5.32 | 1 | 0 | 1 |
| 5.1 | 1.26 | 3.45 | 29 | 24.1 | 62 | 4.3 | 0 | 0 | 0 |
| 8.05 | 1.3 | 5.59 | 19.9 | 16.5 | 75.2 | 4.86 | 1 | 1 | 0 |
| 4.7 | 0.9 | 3 | 22.4 | 18 | 72.7 | 5.07 | 0 | 0 | 0 |
| 6.86 | 1.3 | 4.71 | 31.1 | 16.6 | 62.3 | 6.8 | 0 | 0 | 1 |
| 4.96 | 1.64 | 2.95 | 32.4 | 21.3 | 81.7 | 6.34 | 0 | 0 | 0 |
| 8.66 | 1.31 | 6 | 31 | 20.9 | 61.4 | 4.85 | 1 | 1 | 1 |
| 4.55 | 1.25 | 4.25 | 15 | 14.5 | 76 | 6.79 | 0 | 0 | 0 |
| 5.6 | 1 | 3.7 | 28 | 20 | 75.3 | 5.5 | 0 | 0 | 0 |
| 4.72 | 0.87 | 2.98 | 33.5 | 22 | 76.5 | 8.57 | 0 | 0 | 1 |
| 3.5 | 1.2 | 2.1 | 28.2 | 18.3 | 92.9 | 5.5 | 0 | 0 | 0 |
| 4.2 | 1.4 | 2.5 | 16.9 | 24.1 | 73.6 | 4.74 | 0 | 0 | 0 |
| 4.9 | 1.1 | 3.4 | 17 | 14.1 | 68.2 | 4.8 | 1 | 0 | 0 |
| 6.5 | 1.2 | 4.8 | 36 | 30.1 | 72 | 5.24 | 0 | 0 | 0 |
| 4.35 | 1.17 | 2.75 | 4.7 | 13.1 | 136.2 | 14.9 | 0 | 0 | 0 |
| 2.9 | 0.9 | 1.8 | 26.8 | 20.8 | 66.7 | 4.7 | 0 | 0 | 0 |
| 4.9 | 1.3 | 3.2 | 28.7 | 19.5 | 89 | 4.5 | 0 | 0 | 0 |
| 5.08 | 0.96 | 2.94 | 53.3 | 32.1 | 75.2 | 4.22 | 0 | 0 | 0 |
| 5.64 | 1.06 | 3.88 | 24.8 | 21.1 | 90.4 | 7.05 | 0 | 1 | 1 |
| 5.13 | 0.81 | 3.82 | 69.3 | 47.5 | 77.7 | 4.05 | 1 | 0 | 0 |
| 3.94 | 0.99 | 2.56 | 23 | 23 | 79.2 | 3.2 | 1 | 0 | 0 |
| 3.7 | 1 | 2.4 | 33 | 29.6 | 68.9 | 4.92 | 1 | 1 | 1 |
| 4.5 | 1.9 | 2.6 | 61.7 | 42.6 | 75.4 | 5.5 | 0 | 0 | 0 |
| 5.26 | 0.92 | 3.68 | 27.6 | 22.9 | 57.9 | 5.32 | 1 | 1 | 1 |
| 4.08 | 1.14 | 2.71 | 51.4 | 24.5 | 41.1 | 3.5 | 0 | 0 | 0 |
| 3.78 | 0.89 | 2.52 | 25.1 | 27.2 | 68.3 | 6.01 | 0 | 1 | 0 |
| 4.5 | 1.2 | 2.9 | 15 | 19.8 | 118.1 | 6.3 | 1 | 1 | 0 |
| 4.31 | 1.05 | 3.02 | 15.6 | 20.2 | 78.4 | 8.63 | 0 | 0 | 0 |
| 4.59 | 1.36 | 3.04 | 14.1 | 18.6 | 51.6 | 3.63 | 0 | 0 | 0 |
| 4.05 | 0.95 | 2.81 | 23.8 | 33 | 115.1 | 9.96 | 0 | 0 | 0 |
| 5.05 | 1.05 | 3.61 | 9.2 | 10.9 | 182.3 | 14.32 | 0 | 1 | 1 |
| 4.49 | 1.25 | 3.05 | 8.2 | 13.6 | 87.3 | 5.04 | 0 | 0 | 0 |
| 5.9 | 1.4 | 3.8 | 13.4 | 15 | 70.1 | 5.1 | 0 | 0 | 0 |
| 3.6 | 0.9 | 2.5 | 63.1 | 31 | 70.1 | 5.8 | 0 | 0 | 0 |
| 6.2 | 1.71 | 4.19 | 28.4 | 18.7 | 42.3 | 3.17 | 0 | 0 | 0 |
| 5.69 | 1.04 | 3.68 | 42.9 | 29.9 | 71.4 | 5 | 1 | 1 | 1 |
| 4.2 | 1.2 | 2.7 | 19.2 | 13.8 | 70.5 | 3.66 | 0 | 0 | 0 |
| 5 | 1.2 | 3.6 | 36.7 | 28.5 | 72.6 | 4.2 | 0 | 0 | 0 |
| 3.88 | 1.25 | 2.54 | 20.4 | 20.9 | 82.8 | 6.91 | 0 | 0 | 0 |

| | | | | | | | | | |
|---|---|---|---|---|---|---|---|---|---|
| 4.81 | 1.61 | 2.99 | 5.8 | 15.8 | 56.8 | 2.38 | 1 | 1 | 1 |
| 4.2 | 1.1 | 2.9 | 39.7 | 34 | 53.5 | 6.5 | 0 | 0 | 0 |
| 4.42 | 0.78 | 3.32 | 73.6 | 58.6 | 41.9 | 2.95 | 0 | 1 | 1 |
| 5.25 | 1.22 | 3.75 | 12.8 | 18.4 | 56.2 | 4.88 | 0 | 0 | 0 |
| 3.63 | 0.97 | 2.42 | 49.5 | 34.4 | 63.5 | 4.07 | 0 | 0 | 0 |
| 5.4 | 1.1 | 3.8 | 20.6 | 21.4 | 57 | 4.88 | 0 | 0 | 0 |
| 6.8 | 1.2 | 5 | 20.6 | 21.4 | 87 | 10.39 | 0 | 1 | 1 |
| 4.11 | 1.28 | 2.39 | 43.2 | 36.5 | 52.4 | 3.89 | 0 | 0 | 0 |
| 5.8 | 1.8 | 3.5 | 21.8 | 20.5 | 41.6 | 3.73 | 1 | 1 | 1 |
| 7.09 | 1.45 | 5.14 | 13.9 | 16.5 | 127.1 | 18.74 | 0 | 1 | 0 |
| 4.84 | 1.54 | 3.22 | 15.1 | 28.8 | 73 | 4.72 | 1 | 1 | 0 |
| 5.55 | 1.13 | 4.06 | 21 | 18.9 | 62.6 | 6.11 | 0 | 0 | 0 |
| 5.9 | 1.48 | 3.93 | 11.8 | 17.6 | 59.2 | 4.82 | 0 | 0 | 0 |
| 5 | 1.09 | 3.53 | 14.8 | 17.5 | 49.9 | 4.69 | 0 | 0 | 0 |
| 5.16 | 1.15 | 3.71 | 18.5 | 19.2 | 47.6 | 5.02 | 0 | 0 | 0 |
| 4.54 | 1 | 3.12 | 15 | 14.8 | 47.2 | 4.09 | 0 | 0 | 0 |
| 4.79 | 0.98 | 3.41 | 28.1 | 26.7 | 52.6 | 5.84 | 0 | 0 | 0 |
| 5.09 | 1.83 | 3.04 | 30.7 | 23.3 | 55 | 4.77 | 0 | 0 | 1 |
| 4.62 | 1.05 | 3.11 | 14.8 | 17.2 | 55.6 | 6.14 | 0 | 0 | 0 |
| 3.5 | 1.2 | 2.1 | 14.5 | 18.4 | 56.5 | 6.44 | 1 | 1 | 0 |
| 5.76 | 1.17 | 4.03 | 42.7 | 24.3 | 73.3 | 7.69 | 0 | 0 | 0 |
| 6.52 | 1.9 | 4.31 | 18 | 22.4 | 53.5 | 6.5 | 1 | 1 | 0 |
| 6.59 | 1.48 | 4.4 | 11.7 | 12.5 | 51.9 | 4.87 | 1 | 0 | 1 |
| 5.01 | 1.09 | 3.43 | 13.9 | 20.2 | 55.9 | 3.75 | 0 | 0 | 0 |
| 5.7 | 1.37 | 3.8 | 29.6 | 21.6 | 80.3 | 4.71 | 0 | 0 | 0 |
| 4.81 | 1.24 | 3.05 | 7.8 | 14.5 | 56.6 | 6.28 | 0 | 0 | 0 |
| 5.87 | 1.55 | 3.85 | 16.7 | 12.2 | 51.9 | 3.88 | 1 | 1 | 1 |
| 6.17 | 1.42 | 4.19 | 37.2 | 26.5 | 72 | 6.58 | 0 | 0 | 0 |
| 3.34 | 0.94 | 2.04 | 19.9 | 18.4 | 68.1 | 6.18 | 1 | 1 | 1 |
| 5.1 | 1.4 | 3.5 | 22 | 20 | 57.7 | 5.74 | 0 | 0 | 0 |
| 6.36 | 1.42 | 4.27 | 8.1 | 14.4 | 69.6 | 7.47 | 0 | 0 | 0 |
| 5.11 | 1.16 | 3.33 | 25.8 | 22 | 59 | 3.12 | 0 | 0 | 0 |
| 6.43 | 1.11 | 4.63 | 23.8 | 22.7 | 46.9 | 5.73 | 0 | 0 | 0 |
| 3.6 | 0.93 | 2.34 | 42.5 | 34.7 | 37.6 | 3.92 | 0 | 0 | 0 |
| 5.15 | 1.57 | 3.45 | 24 | 22.1 | 44.9 | 5.78 | 0 | 0 | 0 |
| 6.3 | 1.4 | 4.4 | 11.5 | 15.5 | 44 | 6.3 | 0 | 1 | 0 |
| 6.79 | 1.77 | 4.6 | 60.8 | 35.4 | 54 | 5.61 | 0 | 0 | 0 |
| 4.06 | 1.11 | 2.58 | 19.3 | 14.3 | 36.1 | 4.95 | 0 | 0 | 0 |
| 8 | 1.4 | 5.8 | 71.3 | 52 | 62.5 | 6.75 | 0 | 0 | 0 |
| 5.02 | 0.89 | 3.26 | 50.2 | 26.9 | 59.6 | 4.15 | 0 | 0 | 0 |
| 4.92 | 1.66 | 2.81 | 15.6 | 16.5 | 46.3 | 4.1 | 0 | 0 | 0 |
| 6.4 | 1.3 | 4.9 | 12.3 | 14.8 | 45.1 | 4.74 | 0 | 0 | 0 |
| 6.2 | 1.5 | 4.3 | 11.5 | 12.6 | 46.4 | 6.6 | 0 | 1 | 0 |
| 4.34 | 0.93 | 2.91 | 38.1 | 26.6 | 60 | 5.06 | 0 | 0 | 0 |
| 5.05 | 1.21 | 3.11 | 29.6 | 32.1 | 55.6 | 3.6 | 0 | 0 | 0 |
| 5.48 | 1.06 | 4.19 | 24.1 | 20.2 | 45.2 | 4.06 | 1 | 1 | 1 |
| 5.83 | 1.11 | 4.07 | 74.8 | 52.1 | 81.3 | 5.57 | 1 | 1 | 1 |
| 5.3 | 1.5 | 3.5 | 11.6 | 15.8 | 73.4 | 6.38 | 0 | 0 | 0 |
| 6.8 | 1.2 | 4.71 | 37.2 | 26.7 | 56.1 | 5.78 | 0 | 0 | 0 |
| 3.7 | 1.2 | 2.4 | 9.2 | 12.2 | 47.2 | 6.45 | 0 | 0 | 0 |
| 4.37 | 0.96 | 3.2 | 21.4 | 20.6 | 73 | 6.01 | 0 | 0 | 0 |
| 4.56 | 1.24 | 2.68 | 55.2 | 46 | 45.9 | 3.79 | 1 | 1 | 1 |
| 8.2 | 1.9 | 5.4 | 16.1 | 22.2 | 160.2 | 10.6 | 1 | 1 | 1 |
| 7 | 1.2 | 5.2 | 33.9 | 27.7 | 66.2 | 4.8 | 1 | 1 | 1 |
| 4.24 | 1.37 | 2.71 | 27 | 22.1 | 54.6 | 5.26 | 0 | 0 | 0 |
| 4.97 | 1.1 | 3.23 | 10.1 | 13.3 | 66.5 | 6.1 | 1 | 1 | 1 |
| 7.05 | 1.99 | 4.73 | 10.3 | 15.5 | 57 | 5.39 | 1 | 1 | 1 |
| 9.36 | 1.82 | 5.99 | 15.6 | 22.6 | 84.8 | 9.65 | 1 | 1 | 0 |
| 4.37 | 1.19 | 2.89 | 60.4 | 32.9 | 58.7 | 3.69 | 0 | 0 | 0 |
| 3.8 | 1 | 2.6 | 20.2 | 21.4 | 51.1 | 4.6 | 0 | 0 | 0 |
| 4.63 | 1.62 | 2.9 | 26.1 | 26.5 | 51.6 | 4.2 | 0 | 0 | 0 |
| 3.5 | 1.11 | 2.33 | 8.1 | 13.7 | 44.3 | 3.3 | 0 | 0 | 1 |
| 6.1 | 1.5 | 4.3 | 34.7 | 27.7 | 59 | 5.1 | 0 | 0 | 0 |

| | | | | | | | | | |
|---|---|---|---|---|---|---|---|---|---|
| 7.72 | 1.61 | 4.58 | 17.6 | 19.6 | 60.3 | 6.2 | 0 | 0 | 0 |
| 6.12 | 1.32 | 4.2 | 20.9 | 25.4 | 41.5 | 6.6 | 0 | 0 | 0 |
| 6.3 | 1.4 | 4.4 | 19.1 | 19.9 | 70.2 | 3.92 | 0 | 0 | 0 |
| 6.2 | 1.32 | 4.51 | 17 | 19 | 70.6 | 5.51 | 0 | 0 | 0 |
| 3.7 | 1.3 | 2.3 | 17.5 | 18.9 | 57.7 | 6.3 | 1 | 0 | 0 |
| 5.5 | 1.21 | 3.49 | 19.2 | 22.2 | 51.4 | 8.04 | 0 | 0 | 0 |
| 5.53 | 1.19 | 3.61 | 17.4 | 14.7 | 80 | 5.7 | 1 | 0 | 0 |
| 4.8 | 1.6 | 3.1 | 16.8 | 17.8 | 39.5 | 3.6 | 0 | 0 | 0 |
| 4 | 1.7 | 2.2 | 22 | 27 | 65 | 6.5 | 1 | 0 | 0 |
| 6.2 | 1.5 | 4.4 | 32.8 | 24.4 | 44.7 | 5 | 0 | 0 | 0 |
| 5.31 | 1.39 | 3.6 | 21.5 | 22.4 | 43 | 3.84 | 0 | 0 | 0 |
| 4.56 | 1.49 | 2.79 | 31.3 | 25 | 67.8 | 7 | 0 | 0 | 0 |
| 4.56 | 1.23 | 3.06 | 8.3 | 16.1 | 71.4 | 4.71 | 1 | 1 | 1 |
| 4.99 | 1.27 | 3.27 | 28 | 20.8 | 46.5 | 4.34 | 0 | 0 | 0 |
| 4.74 | 1.49 | 3.03 | 12.1 | 19.5 | 52.1 | 4.3 | 1 | 0 | 1 |
| 3.8 | 1.12 | 2.49 | 16.4 | 17.9 | 86.1 | 7.75 | 0 | 1 | 0 |
| 5.18 | 1.48 | 3.34 | 17.5 | 12.4 | 59.6 | 6.25 | 1 | 0 | 0 |
| 3.59 | 0.9 | 2.32 | 19.1 | 17.6 | 80.1 | 9.52 | 1 | 0 | 0 |
| 4.16 | 1.49 | 2.39 | 21.7 | 22.7 | 48 | 4.75 | 1 | 0 | 1 |
| 5.3 | 1 | 3.7 | 11.6 | 14.8 | 56.8 | 5.3 | 0 | 0 | 0 |
| 7.28 | 1.41 | 5.1 | 14.2 | 16.5 | 62.3 | 5.79 | 1 | 0 | 1 |
| 3.9 | 1.4 | 2.4 | 11.8 | 12.9 | 49 | 4.5 | 1 | 0 | 0 |
| 4.5 | 1.1 | 3.1 | 25.2 | 18.1 | 54.8 | 3.7 | 1 | 0 | 1 |
| 4.4 | 0.81 | 2.75 | 14 | 16.9 | 71.8 | 3.57 | 1 | 1 | 1 |
| 4.2 | 1.1 | 2.8 | 13.8 | 19 | 43.5 | 4.88 | 0 | 0 | 0 |
| 3.3 | 1.3 | 1.9 | 23.9 | 24.7 | 54.8 | 4.77 | 1 | 1 | 0 |
| 5.1 | 1.27 | 3.58 | 13.4 | 16.2 | 53.2 | 5.27 | 0 | 0 | 0 |
| 5.4 | 1.3 | 3.8 | 13.1 | 15.8 | 48.8 | 5.54 | 0 | 1 | 0 |
| 3.54 | 1.06 | 2.34 | 26.2 | 37.8 | 42.9 | 5.51 | 0 | 0 | 0 |
| 4.03 | 1.19 | 2.52 | 22.5 | 20 | 50.5 | 5.75 | 0 | 0 | 0 |
| 2.85 | 0.96 | 1.83 | 24.9 | 25.3 | 44.6 | 6.67 | 0 | 0 | 0 |
| 4.19 | 1.03 | 3.08 | 22.1 | 17.7 | 42.5 | 4.13 | 0 | 0 | 0 |
| 5.91 | 1.49 | 3.45 | 26.2 | 27 | 53.6 | 6.61 | 0 | 0 | 0 |
| 6.35 | 1.33 | 4.49 | 18.3 | 19.9 | 47.5 | 4.38 | 1 | 1 | 1 |
| 3.9 | 1.04 | 2.55 | 27.6 | 25.6 | 42 | 5.5 | 1 | 0 | 0 |
| 4.94 | 1.12 | 3.4 | 10.6 | 12.3 | 45.6 | 4.18 | 0 | 0 | 0 |
| 5.6 | 1.4 | 3.8 | 17.4 | 16.8 | 47.4 | 6.5 | 0 | 0 | 1 |
| 5.5 | 1.57 | 3.7 | 13.5 | 14.7 | 42.3 | 4.58 | 0 | 0 | 0 |
| 4.41 | 1.36 | 2.78 | 11.3 | 17 | 54.1 | 4.8 | 0 | 0 | 0 |
| 6.15 | 1.41 | 4.11 | 4.9 | 17 | 54.7 | 5.17 | 1 | 0 | 1 |
| 5.39 | 1.93 | 3.33 | 16.3 | 16.4 | 68.4 | 8.15 | 0 | 0 | 0 |
| 5.98 | 1.27 | 4.09 | 18.8 | 24.6 | 64.7 | 7.4 | 0 | 0 | 0 |
| 5 | 1.34 | 3.12 | 23.3 | 29.1 | 45.7 | 5.25 | 0 | 0 | 1 |
| 3.48 | 1.2 | 1.47 | 31.8 | 24.5 | 61 | 6.8 | 0 | 1 | 0 |
| 6.52 | 1.5 | 4.05 | 18 | 26.2 | 66.3 | 4.1 | 0 | 0 | 0 |
| 4.72 | 1.01 | 3.34 | 15.3 | 20.1 | 62.3 | 5.4 | 0 | 1 | 0 |
| 6.04 | 1.45 | 4.24 | 13.7 | 13.3 | 131.8 | 10.55 | 1 | 1 | 1 |
| 3.5 | 0.74 | 2.38 | 16.6 | 19.7 | 60.6 | 4.4 | 1 | 0 | 0 |
| 5.23 | 1.45 | 3.32 | 44.8 | 83.1 | 57 | 4.36 | 1 | 1 | 1 |
| 4.39 | 1.07 | 3.03 | 15.6 | 18.6 | 64.9 | 8.31 | 1 | 1 | 1 |
| 5.02 | 1.42 | 3.41 | 18.3 | 16 | 54.9 | 4.14 | 1 | 1 | 0 |
| 4.02 | 1.27 | 2.53 | 13.4 | 16.3 | 56 | 6.99 | 0 | 0 | 0 |
| 5.74 | 1.62 | 3.72 | 20.7 | 20.5 | 56.5 | 4.63 | 0 | 0 | 0 |
| 4.4 | 1.1 | 3.1 | 18.6 | 28 | 157.6 | 7.52 | 0 | 0 | 0 |
| 5.41 | 1.19 | 3.85 | 9.9 | 19.7 | 60 | 4.6 | 0 | 0 | 0 |
| 3.58 | 0.94 | 2.43 | 15.2 | 23.4 | 52.5 | 3.66 | 0 | 0 | 0 |
| 4.63 | 1.28 | 2.91 | 15.6 | 19.8 | 80.8 | 8.08 | 0 | 1 | 0 |
| 4.92 | 1.41 | 3.28 | 20.3 | 20.4 | 65.3 | 5.8 | 0 | 0 | 0 |
| 4.8 | 1.1 | 3.3 | 30.8 | 26.6 | 60.9 | 4.4 | 0 | 0 | 0 |
| 3.4 | 0.84 | 2.21 | 17.2 | 13.6 | 50 | 6.98 | 0 | 0 | 1 |
| 4.97 | 0.89 | 3.59 | 30 | 21.7 | 71.6 | 4 | 0 | 0 | 0 |
| 5.1 | 1.4 | 3 | 11.2 | 16.9 | 55.9 | 4.96 | 0 | 0 | 0 |
| 7.36 | 1.71 | 5.11 | 12.8 | 20.3 | 64.7 | 8.67 | 1 | 0 | 0 |

| | | | | | | | | | |
|---|---|---|---|---|---|---|---|---|---|
| 4.81 | 1.95 | 2.75 | 18.2 | 20.8 | 64.3 | 5.5 | 0 | 0 | 0 |
| 4.37 | 0.96 | 2.84 | 10.3 | 19.1 | 116.7 | 8.13 | 0 | 0 | 0 |
| 3.96 | 1.48 | 2.09 | 15 | 23 | 42.2 | 5.1 | 0 | 0 | 0 |
| 6.36 | 1.57 | 4.22 | 23.9 | 25.7 | 73 | 7.86 | 1 | 1 | 1 |
| 9.07 | 1.85 | 6.33 | 26.7 | 28.3 | 48.9 | 4.2 | 0 | 0 | 0 |
| 4.9 | 1.8 | 2.9 | 35.8 | 22.5 | 44.9 | 4.6 | 0 | 0 | 0 |
| 4.79 | 1.04 | 3.42 | 19.1 | 17.6 | 38.2 | 3.62 | 0 | 0 | 0 |
| 3.65 | 0.69 | 2.05 | 9 | 11.8 | 69.6 | 6.6 | 1 | 0 | 0 |
| 6.31 | 1.62 | 4.36 | 33.9 | 23.9 | 53 | 4.12 | 0 | 0 | 0 |
| 7.74 | 1.57 | 5.29 | 15.4 | 12.2 | 83.3 | 5.94 | 0 | 0 | 0 |
| 4.07 | 1.06 | 2.63 | 24.4 | 21.3 | 53.7 | 4.91 | 0 | 0 | 0 |
| 4.55 | 1.49 | 2.9 | 17.6 | 15.9 | 62.7 | 4.35 | 0 | 0 | 0 |
| 5.8 | 1.7 | 3.9 | 12.5 | 18.2 | 56.4 | 4.3 | 0 | 0 | 0 |
| 4.46 | 0.9 | 3.27 | 11.5 | 14.5 | 69.9 | 7.32 | 1 | 1 | 1 |
| 6.71 | 1.28 | 4.91 | 19.7 | 17.1 | 61.9 | 3.34 | 0 | 0 | 0 |
| 3.9 | 1.24 | 2.52 | 17 | 23.1 | 49.8 | 4.57 | 0 | 0 | 0 |
| 4.92 | 1.4 | 3.41 | 14.1 | 16.6 | 49.8 | 3.28 | 0 | 0 | 1 |
| 5.5 | 1.5 | 3.6 | 19.8 | 26.5 | 61.9 | 5.4 | 0 | 0 | 0 |
| 4.9 | 1.4 | 3.4 | 44.1 | 31.8 | 58.6 | 4.63 | 1 | 1 | 1 |
| 4.3 | 1.29 | 2.64 | 27.4 | 22 | 60.5 | 5.08 | 0 | 0 | 0 |
| 3.65 | 0.99 | 2.27 | 118.7 | 61.5 | 72.4 | 7.15 | 0 | 0 | 0 |
| 4.94 | 0.83 | 3.26 | 61.9 | 32.7 | 48.9 | 3.82 | 1 | 1 | 0 |
| 6.13 | 1.16 | 4.08 | 9.6 | 15.1 | 42.2 | 6.15 | 0 | 0 | 0 |
| 5.67 | 1.2 | 3.95 | 10.8 | 12.3 | 68.4 | 6.1 | 0 | 0 | 0 |
| 5.3 | 1.1 | 3.9 | 32.9 | 29.5 | 59 | 4.28 | 1 | 1 | 1 |
| 4 | 1.4 | 2.7 | 12.7 | 14.3 | 72.2 | 6.02 | 1 | 0 | 0 |
| 5.04 | 1.1 | 3.48 | 29.8 | 24.3 | 55.9 | 5.7 | 1 | 0 | 0 |
| 5.17 | 1.55 | 3.26 | 20.1 | 28.9 | 69.8 | 4.5 | 0 | 0 | 0 |
| 6.3 | 1.4 | 4.3 | 26.6 | 28.1 | 45 | 6.4 | 1 | 0 | 1 |
| 4.29 | 0.72 | 3.3 | 6.6 | 13.2 | 207.7 | 18.59 | 0 | 1 | 1 |
| 4.7 | 1.59 | 2.81 | 13.8 | 19.7 | 58.8 | 3.53 | 0 | 0 | 0 |
| 4.8 | 1.3 | 3.2 | 20.2 | 21.6 | 41.4 | 3.1 | 0 | 0 | 0 |
| 5.7 | 1.4 | 3.9 | 21 | 23.5 | 77 | 6.7 | 1 | 1 | 1 |
| 3.03 | 0.96 | 1.96 | 13.1 | 20.4 | 56 | 4.72 | 0 | 0 | 0 |
| 5.51 | 1.39 | 3.62 | 11 | 23.3 | 66.9 | 4.01 | 1 | 0 | 0 |
| 2.83 | 0.9 | 1.76 | 8.4 | 13 | 51.6 | 6.24 | 1 | 1 | 1 |
| 5.23 | 1.68 | 3.19 | 28.6 | 26.5 | 45.8 | 4.77 | 0 | 0 | 0 |
| 4.91 | 1.51 | 3.17 | 24.4 | 24.4 | 60.2 | 3.68 | 0 | 0 | 0 |
| 4.91 | 1.15 | 3.38 | 36.4 | 25.4 | 59.1 | 4.51 | 0 | 0 | 0 |
| 5.7 | 1.5 | 3.6 | 13.8 | 16.1 | 42.7 | 4.2 | 0 | 0 | 0 |
| 5.03 | 1.38 | 3.38 | 33.8 | 25 | 46.2 | 6.04 | 0 | 0 | 0 |
| 4.75 | 1.17 | 3.23 | 9.5 | 16.1 | 51.4 | 6 | 0 | 0 | 0 |
| 4.44 | 1.29 | 3.01 | 10.2 | 16.9 | 47.3 | 4.66 | 0 | 0 | 0 |
| 6 | 1.2 | 4.2 | 78.8 | 36.6 | 50.3 | 2.63 | 0 | 0 | 0 |
| 4.31 | 0.99 | 2.89 | 10.2 | 15 | 49.9 | 5.25 | 0 | 0 | 0 |
| 5.37 | 1.25 | 3.9 | 95.5 | 54.5 | 64.3 | 5.6 | 0 | 0 | 0 |
| 4.91 | 1.25 | 3.55 | 30.8 | 22 | 43.6 | 6.13 | 1 | 1 | 1 |
| 5.46 | 1.31 | 3.52 | 26.8 | 30.1 | 83.6 | 3.53 | 0 | 0 | 0 |
| 3.8 | 1.4 | 2.2 | 18.8 | 16.7 | 60.5 | 4.5 | 0 | 0 | 0 |
| 4.5 | 1.2 | 3 | 7.8 | 15.4 | 41.5 | 3.28 | 1 | 0 | 0 |
| 4.98 | 1.15 | 3.5 | 10.2 | 17.6 | 41.5 | 3.78 | 0 | 0 | 0 |
| 5.3 | 1.7 | 3.3 | 19.3 | 18.8 | 58.7 | 5.37 | 1 | 0 | 0 |
| 4.55 | 1.63 | 2.82 | 22.3 | 24.5 | 42.7 | 4.7 | 0 | 0 | 0 |
| 5.63 | 1.32 | 3.89 | 15.5 | 19.3 | 55.1 | 4.69 | 0 | 0 | 0 |
| 5.76 | 1.23 | 4.03 | 76.8 | 65 | 52.7 | 4.19 | 0 | 0 | 0 |
| 5.03 | 1.19 | 3.34 | 23.3 | 20.4 | 84.1 | 9.1 | 0 | 0 | 0 |
| 4.41 | 1.1 | 3.12 | 15.7 | 17.6 | 56.4 | 4.9 | 0 | 0 | 0 |
| 6 | 1.5 | 4.1 | 38.1 | 25.3 | 62.6 | 4.3 | 0 | 0 | 0 |
| 5.16 | 1.3 | 3.67 | 38 | 37.9 | 53.2 | 4.97 | 0 | 0 | 0 |
| 5.8 | 1.3 | 4.3 | 20.3 | 23.1 | 63.9 | 4.9 | 1 | 1 | 1 |
| 5 | 1.1 | 3.8 | 9.9 | 14.7 | 61.9 | 3.6 | 1 | 1 | 1 |
| 5.5 | 1.4 | 3.7 | 13.8 | 19.7 | 51.9 | 4 | 0 | 0 | 0 |
| 3.92 | 1.18 | 2.57 | 24.8 | 20.9 | 65.6 | 3.3 | 0 | 0 | 0 |

| | | | | | | | | | |
|---|---|---|---|---|---|---|---|---|---|
| 6.17 | 1.26 | 4.31 | 91.8 | 49.9 | 63.2 | 3.57 | 0 | 0 | 0 |
| 4.69 | 1.27 | 3.03 | 38.3 | 35.6 | 54.2 | 5.39 | 0 | 0 | 0 |
| 5.35 | 1.25 | 3.38 | 25.3 | 24.3 | 90 | 4.63 | 1 | 0 | 0 |
| 7.44 | 1.32 | 5.5 | 25.2 | 24 | 41.6 | 5.61 | 0 | 0 | 0 |
| 5.3 | 1.4 | 3.2 | 23 | 22.2 | 49.1 | 5.62 | 0 | 0 | 0 |
| 3.77 | 1.38 | 2.21 | 11.7 | 17.3 | 79.3 | 5.24 | 1 | 1 | 1 |
| 5.8 | 2.2 | 3.3 | 16.5 | 27.1 | 57.9 | 6.1 | 1 | 1 | 1 |
| 5.66 | 1.66 | 3.39 | 14.9 | 20.8 | 56.3 | 5.3 | 0 | 0 | 0 |
| 4.55 | 0.94 | 3.17 | 16.4 | 15.9 | 54.5 | 4.49 | 0 | 0 | 0 |
| 5.7 | 1 | 4 | 14.2 | 17 | 43.1 | 2.9 | 0 | 0 | 0 |
| 7.24 | 1.52 | 5.32 | 12.6 | 13.2 | 50.8 | 5.3 | 0 | 0 | 0 |
| 5.71 | 1.65 | 3.9 | 16.9 | 18.1 | 53 | 4.38 | 1 | 1 | 1 |
| 4.9 | 1.8 | 2.8 | 12.9 | 22.4 | 59 | 4.7 | 1 | 1 | 1 |
| 4.6 | 1.1 | 3.3 | 23.3 | 26.7 | 85.2 | 5.72 | 0 | 1 | 0 |
| 4.9 | 1 | 3.2 | 44.6 | 36.4 | 56 | 4.9 | 0 | 0 | 0 |
| 4.61 | 1.07 | 2.85 | 25 | 26.8 | 50.4 | 5.17 | 0 | 1 | 0 |
| 4.02 | 1.1 | 2.8 | 22.8 | 24.3 | 55.1 | 4.18 | 1 | 0 | 0 |
| 5 | 2.1 | 2.5 | 27.6 | 19.8 | 44.3 | 4.18 | 0 | 0 | 0 |
| 3.74 | 1.45 | 2.01 | 25.3 | 25.7 | 76.9 | 7.91 | 1 | 1 | 1 |
| 4.8 | 0.9 | 3.5 | 15.2 | 14.5 | 49.4 | 4.1 | 0 | 0 | 0 |
| 5.8 | 1.2 | 4.1 | 43.9 | 38.7 | 52.9 | 3.9 | 0 | 0 | 0 |
| 3.4 | 1 | 2 | 40.9 | 22.3 | 77.3 | 6 | 1 | 1 | 1 |
| 3.34 | 1.46 | 1.71 | 19.7 | 20.5 | 73.2 | 6.23 | 1 | 1 | 1 |
| 4.8 | 1.6 | 3 | 25.6 | 37 | 92.8 | 4.8 | 0 | 0 | 0 |
| 5.59 | 1.1 | 3.99 | 14.3 | 18.3 | 71.7 | 6.27 | 0 | 0 | 0 |
| 4.24 | 0.8 | 2.74 | 9.8 | 12.6 | 70.4 | 5.36 | 0 | 0 | 0 |
| 5.4 | 1 | 3.9 | 15.9 | 19.6 | 75 | 6.2 | 0 | 0 | 1 |
| 5.25 | 1.17 | 3.39 | 22.4 | 20.5 | 61.1 | 5.97 | 0 | 0 | 0 |
| 3.9 | 1.01 | 2.69 | 16.7 | 18.6 | 50.3 | 4.7 | 0 | 0 | 0 |
| 3.99 | 0.95 | 2.66 | 13.9 | 22.2 | 101.1 | 5.05 | 1 | 0 | 0 |
| 5.4 | 0.92 | 3.87 | 18 | 20 | 67.2 | 5.76 | 0 | 0 | 0 |
| 4.89 | 1 | 3.05 | 10.8 | 13.6 | 81.4 | 6.77 | 0 | 1 | 1 |
| 5.43 | 3.39 | 0.86 | 28 | 19 | 67 | 3.8 | 0 | 0 | 0 |
| 6.15 | 1.2 | 4.37 | 34 | 26 | 65.8 | 4.95 | 0 | 1 | 0 |
| 4.61 | 0.97 | 3.17 | 46.6 | 37.9 | 71.9 | 7.58 | 0 | 0 | 0 |
| 6.01 | 0.83 | 3.82 | 51.7 | 38 | 61.4 | 5.66 | 0 | 0 | 0 |
| 3.8 | 1 | 2.6 | 25.2 | 22 | 87 | 6.37 | 0 | 0 | 0 |
| 4.3 | 0.8 | 3 | 36.1 | 32.9 | 66.6 | 4.97 | 0 | 0 | 0 |
| 4.64 | 1.26 | 3 | 10.1 | 20.2 | 69.9 | 5.49 | 1 | 1 | 0 |
| 4.84 | 1.19 | 3.2 | 91.5 | 55.8 | 70.8 | 4.56 | 1 | 1 | 1 |
| 5.53 | 0.9 | 3.39 | 14.9 | 18.1 | 76.4 | 4.27 | 0 | 0 | 0 |
| 3.4 | 1.1 | 2.2 | 23 | 18 | 75.5 | 6.9 | 1 | 0 | 1 |
| 5.17 | 1.03 | 3.44 | 25.2 | 21.7 | 53.2 | 6.1 | 0 | 0 | 0 |
| 3.28 | 0.62 | 2.69 | 74.7 | 47.4 | 57.4 | 6.9 | 0 | 0 | 0 |
| 5.27 | 1.5 | 3.39 | 25 | 26.6 | 103.7 | 8.37 | 0 | 0 | 0 |
| 5.16 | 1.35 | 3.47 | 13.1 | 17.6 | 80.4 | 7.92 | 0 | 0 | 0 |
| 2.5 | 0.8 | 1.5 | 23.5 | 24.7 | 95.4 | 5.83 | 1 | 1 | 1 |
| 5.49 | 1.05 | 3.89 | 14.4 | 16.7 | 62.7 | 5.3 | 1 | 1 | 0 |
| 4.02 | 1.1 | 2.73 | 26.2 | 24.7 | 134.8 | 9.42 | 0 | 0 | 0 |
| 5.03 | 0.87 | 3.32 | 14 | 16.1 | 64.9 | 5.53 | 0 | 0 | 0 |
| 5.69 | 2.05 | 3.13 | 35.9 | 33.5 | 59.8 | 3.01 | 0 | 1 | 0 |
| 5.53 | 1.16 | 4.03 | 29.4 | 25.8 | 75.3 | 5.5 | 0 | 0 | 0 |
| 4.28 | 0.96 | 3.08 | 53.2 | 47.3 | 80.9 | 8.12 | 1 | 1 | 1 |
| 4.95 | 0.94 | 3.64 | 29.4 | 24.8 | 72.8 | 4.2 | 0 | 0 | 0 |
| 6.01 | 1.26 | 4.45 | 54.6 | 39.3 | 92.7 | 4 | 0 | 0 | 0 |
| 5.6 | 1 | 3.9 | 32.8 | 21.1 | 57.9 | 4.64 | 0 | 0 | 0 |
| 3.25 | 0.8 | 1.26 | 26 | 22.4 | 67.9 | 4.9 | 1 | 1 | 1 |
| 4.49 | 0.95 | 3.19 | 17.3 | 18.3 | 78.8 | 4.71 | 1 | 0 | 0 |
| 5.51 | 1.16 | 3.7 | 38.3 | 23.2 | 78.8 | 4.71 | 0 | 0 | 0 |
| 3.2 | 1.3 | 0.7 | 20.7 | 20.4 | 75.8 | 3.68 | 0 | 0 | 0 |
| 4.95 | 1.56 | 2.85 | 50.4 | 56.5 | 60.3 | 5.61 | 0 | 0 | 0 |
| 5.35 | 1.04 | 4.03 | 19 | 15.6 | 57.6 | 5.2 | 0 | 0 | 0 |
| 4.72 | 0.87 | 3.4 | 23.1 | 18.4 | 79.3 | 7.21 | 0 | 0 | 0 |

| | | | | | | | | | |
|---|---|---|---|---|---|---|---|---|---|
| 4.46 | 0.97 | 3.13 | 21.4 | 10.9 | 79 | 5.7 | 0 | 0 | 0 |
| 4.17 | 1.5 | 2.52 | 23.1 | 24 | 75.5 | 5.31 | 1 | 0 | 0 |
| 4.8 | 1.2 | 3.2 | 29.8 | 25.1 | 83.7 | 4.57 | 0 | 0 | 0 |
| 5 | 1.1 | 3.2 | 15.4 | 15.9 | 82.3 | 5.01 | 0 | 0 | 0 |
| 4.31 | 1.06 | 2.89 | 27.1 | 24 | 72 | 3.97 | 0 | 0 | 0 |
| 6.32 | 1.39 | 4.63 | 14 | 17.3 | 113.3 | 5.5 | 1 | 0 | 0 |
| 3.44 | 1.38 | 1.93 | 18.6 | 20.1 | 76.9 | 7.42 | 0 | 0 | 0 |
| 5.35 | 1.49 | 3.51 | 22.2 | 22.1 | 55.9 | 4.41 | 1 | 1 | 1 |
| 4.53 | 0.92 | 3.3 | 33.7 | 27.4 | 72.9 | 5.6 | 0 | 0 | 0 |
| 6.7 | 1.63 | 4.77 | 13.1 | 13.6 | 46 | 4.65 | 1 | 1 | 1 |
| 3.08 | 0.83 | 1.97 | 49.2 | 34.5 | 86.2 | 5.69 | 0 | 1 | 1 |
| 3.94 | 1.04 | 2.46 | 19.2 | 16.9 | 73.3 | 7.77 | 1 | 1 | 1 |
| 5.09 | 1.07 | 3.5 | 26.05 | 22.98 | 80.16 | 5.49 | 0 | 1 | 1 |
| 5.02 | 1.7 | 2.89 | 25.3 | 34.7 | 90.3 | 4.76 | 1 | 0 | 1 |
| 4.09 | 1.28 | 2.06 | 15.86 | 16.39 | 64.9 | 8.2 | 0 | 0 | 1 |
| 4.58 | 1.48 | 2.7 | 26.5 | 23.4 | 66.6 | 6.54 | 1 | 0 | 1 |
| 4.49 | 1.22 | 2.88 | 24.9 | 20 | 78.1 | 7.33 | 1 | 1 | 1 |
| 4.28 | 1.55 | 2.8 | 20.2 | 20 | 71.2 | 6.92 | 0 | 1 | 0 |
| 7.75 | 1.6 | 5.04 | 75.1 | 49.6 | 78.2 | 6.57 | 1 | 0 | 1 |
| 4.79 | 1.17 | 3.25 | 17 | 18.6 | 68.7 | 5.65 | 1 | 0 | 1 |
| 6.08 | 1.39 | 4.01 | 11.9 | 21.3 | 54.4 | 5.2 | 1 | 1 | 1 |
| 4.4 | 0.87 | 3.07 | 97.1 | 56.4 | 73.4 | 3.91 | 0 | 0 | 0 |
| 5.05 | 1.44 | 3.22 | 49.7 | 39.1 | 50.4 | 3.82 | 1 | 1 | 1 |
| 5.36 | 1.31 | 3.6 | 16.9 | 20.4 | 90.3 | 7.08 | 1 | 1 | 1 |
| 3.89 | 1.14 | 2.32 | 18.3 | 17.9 | 68.3 | 6.19 | 1 | 1 | 1 |
| 3.25 | 1.2 | 1.88 | 38.5 | 17.6 | 105.6 | 10.51 | 0 | 0 | 1 |
| 3.77 | 1.19 | 2.5 | 11.1 | 14.3 | 53.9 | 4.03 | 0 | 0 | 0 |
| 4.86 | 1.26 | 3.29 | 31.7 | 22.5 | 70.6 | 5.4 | 1 | 1 | 0 |
| 5.28 | 1.35 | 3.41 | 23.1 | 20.9 | 48.9 | 5.8 | 1 | 0 | 1 |
| 6.03 | 1.09 | 4.17 | 19 | 17.7 | 56.9 | 6.66 | 1 | 1 | 0 |
| 4.58 | 1.17 | 3.08 | 24.5 | 20.4 | 113.8 | 9.22 | 0 | 0 | 0 |
| 5.1 | 1.39 | 3.35 | 29.8 | 27.2 | 50.2 | 6.72 | 0 | 0 | 1 |
| 4.89 | 1.4 | 3.05 | 28.6 | 31 | 73.9 | 6.02 | 1 | 0 | 1 |
| 4.97 | 1.39 | 3.2 | 24.7 | 21.2 | 43.9 | 5.3 | 0 | 0 | 1 |
| 4.85 | 1.41 | 3.09 | 13.6 | 19 | 110.87 | 827 | 0 | 0 | 1 |
| 3.24 | 0.95 | 2.05 | 23.4 | 24 | 58.8 | 4.48 | 1 | 1 | 0 |
| 4.07 | 1.35 | 2.47 | 20.5 | 20.2 | 54.13 | 4.24 | 1 | 0 | 1 |
| 2.39 | 0.79 | 1.48 | 19.2 | 15.3 | 37.9 | 3.88 | 0 | 0 | 0 |
| 4.58 | 1.48 | 2.88 | 15.5 | 17.3 | 114.1 | 9.79 | 1 | 1 | 0 |

| RBCs | sex | Age | AG | HbA1c | Hb | course | HGI | ehba1c | TG |
|---|---|---|---|---|---|---|---|---|---|
| 21 | 1 | 51 | 8.2 | 3.7 | 163 | 12 | -3.07 | 6.8 | 0.73 |
| 47 | 1 | 51 | 8.4 | 4.9 | 143 | 8 | -2 | 6.9 | 1.22 |
| 61 | 1 | 65 | 7.4 | 6.3 | 147 | 3 | 0.03 | 6.3 | 1.98 |
| 64 | 1 | 59 | 11.6 | 6.3 | 172 | 10 | -2.6 | 8.9 | 1.17 |
| 65 | 1 | 46 | 7.2 | 5.8 | 162 | 10 | -0.34 | 6.1 | 2.3 |
| 67 | 1 | 67 | 6.9 | 5.4 | 165 | 7 | -0.55 | 6 | 2.25 |
| 67 | 1 | 53 | 11.8 | 7.4 | 149 | 12 | -1.63 | 9 | 1.5 |
| 69 | 1 | 36 | 8.2 | 5.9 | 149 | 4 | -0.87 | 6.8 | 6.34 |
| 71 | 1 | 35 | 8.4 | 7.4 | 148 | 5 | 0.5 | 6.9 | 1.32 |
| 71 | 1 | 66 | 7.4 | 5.2 | 113 | 21 | -1.07 | 6.3 | 0.83 |
| 72 | 1 | 42 | 7.3 | 6 | 142 | 13 | -0.21 | 6.2 | 1.87 |
| 74 | 1 | 60 | 7 | 5.4 | 170 | 8 | -0.62 | 6 | 2.73 |
| 74 | 1 | 52 | 7.2 | 6.7 | 166 | 6 | 0.56 | 6.1 | 4.06 |
| 74 | 1 | 62 | 9.6 | 6 | 151 | 6 | -1.65 | 7.6 | 1.46 |
| 76 | 1 | 57 | 10.2 | 7.2 | 132 | 23 | -0.82 | 8 | 0.63 |
| 77 | 1 | 52 | 8.5 | 6.4 | 148 | 8 | -0.56 | 7 | 11.95 |
| 77 | 1 | 62 | 8.2 | 6.1 | 147 | 8 | -0.67 | 6.8 | 5.8 |
| 77 | 1 | 84 | 15.3 | 10.6 | 144 | 10 | -0.62 | 11.2 | 3.3 |
| 81 | 1 | 63 | 7.9 | 5.5 | 146 | 2 | -1.08 | 6.6 | 3.54 |
| 83 | 1 | 30 | 7.2 | 5.4 | 167 | 2 | -0.74 | 6.1 | 1.41 |
| 84 | 1 | 69 | 10.1 | 7.2 | 164 | 2 | -0.76 | 8 | 1.53 |
| 87 | 1 | 73 | 7.9 | 5.8 | 175 | 7 | -0.78 | 6.6 | 1.52 |
| 88 | 1 | 61 | 7.5 | 5.7 | 149 | 5 | -0.63 | 6.3 | 1.5 |
| 90 | 1 | 31 | 6.3 | 6 | 133 | 18 | 0.42 | 5.6 | 0.9 |
| 90 | 1 | 51 | 9.8 | 8.2 | 159 | 17 | 0.43 | 7.8 | 0.69 |
| 90 | 1 | 38 | 7.3 | 6 | 164 | 5 | -0.21 | 6.2 | 1.87 |
| 90 | 1 | 78 | 8.6 | 6.9 | 179 | 1 | -0.12 | 7 | 0.79 |
| 93 | 1 | 40 | 8.5 | 6.2 | 167 | 0 | -0.76 | 7 | 2.29 |
| 94 | 1 | 52 | 8.2 | 5.9 | 143 | 2 | -0.87 | 6.8 | 1 |
| 95 | 1 | 70 | 7.5 | 7.6 | 160 | 19 | 1.27 | 6.3 | 0.76 |
| 96 | 1 | 78 | 8.8 | 7.1 | 148 | 6 | -0.05 | 7.1 | 0.6 |
| 97 | 1 | 71 | 9 | 6 | 174 | 1 | -1.27 | 7.3 | 1.02 |
| 97 | 1 | 57 | 9.8 | 7.2 | 147 | 6 | -0.57 | 7.8 | 1.2 |
| 105 | 1 | 66 | 7.3 | 6.4 | 81 | 0 | 0.19 | 6.2 | 1.39 |
| 108 | 1 | 63 | 7.4 | 5.2 | 141 | 7 | -1.07 | 6.3 | 2.33 |
| 108 | 1 | 63 | 8.3 | 5.6 | 134 | 1 | -1.23 | 6.8 | 1.65 |
| 108 | 1 | 59 | 7.4 | 7.2 | 147 | 20 | 0.93 | 6.3 | 1.62 |
| 127 | 1 | 59 | 6 | 6 | 123 | 19 | 0.61 | 5.4 | 1.25 |
| 131 | 1 | 67 | 7.9 | 6.1 | 150 | 4 | -0.48 | 6.6 | 0.65 |
| 149 | 1 | 66 | 9.1 | 7.7 | 143 | 1 | 0.37 | 7.3 | 2.02 |
| 153 | 1 | 58 | 6.4 | 5.2 | 134 | 3 | -0.44 | 5.6 | 3.6 |
| 167 | 1 | 28 | 5.6 | 5.5 | 141 | 8 | 0.36 | 5.1 | 3.06 |
| 168 | 1 | 75 | 7 | 5.5 | 130 | 16 | -0.52 | 6 | 2.14 |
| 55 | 2 | 49 | 10.2 | 7 | 145 | 13 | -1.02 | 8 | 2 |
| 58 | 2 | 41 | 8.6 | 6.2 | 138 | 3 | -0.82 | 7 | 2.5 |
| 60 | 2 | 64 | 6.8 | 5.4 | 119 | 20 | -0.49 | 5.9 | 1.1 |
| 63 | 2 | 57 | 10.5 | 7.3 | 128 | 10 | -0.91 | 8.2 | 1.3 |
| 64 | 2 | 61 | 8.8 | 6.7 | 146 | 15 | -0.45 | 7.1 | 0.8 |
| 74 | 2 | 38 | 7.3 | 5.3 | 133 | 5 | -0.91 | 6.2 | 1.29 |
| 76 | 2 | 65 | 8 | 7.1 | 134 | 1 | 0.46 | 6.6 | 0.7 |
| 77 | 2 | 58 | 9.1 | 6 | 142 | 8 | -1.33 | 7.3 | 2.23 |
| 77 | 2 | 41 | 9.5 | 6.2 | 143 | 10 | -1.39 | 7.6 | 1.88 |
| 78 | 2 | 52 | 8.8 | 7.3 | 133 | 6 | 0.15 | 7.1 | 1.04 |
| 81 | 2 | 43 | 7.1 | 6.2 | 138 | 2 | 0.12 | 6.1 | 2.24 |
| 83 | 2 | 66 | 7.8 | 6.9 | 154 | 3 | 0.38 | 6.5 | 1.92 |
| 83 | 2 | 66 | 12.4 | 7 | 149 | 18 | -2.4 | 9.4 | 2.7 |
| 84 | 2 | 63 | 8.7 | 5.8 | 133 | 5 | -1.28 | 7.1 | 0.88 |
| 84 | 2 | 64 | 9.2 | 6.3 | 129 | 30 | -1.1 | 7.4 | 1.8 |
| 85 | 2 | 63 | 8.5 | 5.7 | 131 | 2 | -1.26 | 7 | 5.72 |
| 85 | 2 | 55 | 12.2 | 8.6 | 94 | 3 | -0.68 | 9.3 | 1.68 |
| 89 | 2 | 70 | 7.7 | 6.2 | 133 | 15 | -0.26 | 6.5 | 0.89 |
| 92 | 2 | 52 | 11.9 | 7.2 | 146 | 6 | -1.89 | 9.1 | 1.14 |

| | | | | | | | | | |
|---|---|---|---|---|---|---|---|---|---|
| 93 | 2 | 54 | 6.3 | 5.6 | 163 | 20 | 0.02 | 5.6 | 1.21 |
| 93 | 2 | 60 | 9.1 | 6.4 | 139 | 12 | -0.93 | 7.3 | 1.78 |
| 93 | 2 | 65 | 6.5 | 5.3 | 88 | 2 | -0.4 | 5.7 | 1.26 |
| 94 | 2 | 78 | 8.8 | 7.2 | 135 | 11 | 0.05 | 7.1 | 4.96 |
| 95 | 2 | 42 | 7.1 | 6 | 85 | 47 | -0.08 | 6.1 | 1.28 |
| 95 | 2 | 56 | 7.8 | 6.6 | 144 | 20 | 0.08 | 6.5 | 1.2 |
| 97 | 2 | 32 | 11.8 | 7.9 | 146 | 0 | -1.13 | 9 | 0.9 |
| 97 | 2 | 53 | 10.6 | 8.6 | 136 | 7 | 0.32 | 8.3 | 1.89 |
| 101 | 2 | 52 | 9.6 | 7.6 | 139 | 8 | -0.05 | 7.6 | 1.4 |
| 105 | 2 | 61 | 8.2 | 7.3 | 129 | 15 | 0.53 | 6.8 | 1 |
| 105 | 2 | 68 | 8.1 | 7.1 | 144 | 5 | 0.39 | 6.7 | 1.9 |
| 106 | 2 | 61 | 8.5 | 6.6 | 141 | 10 | -0.36 | 7 | 1.6 |
| 107 | 2 | 52 | 8.5 | 6.3 | 134 | 8 | -0.66 | 7 | 2.78 |
| 107 | 2 | 52 | 8.7 | 6.8 | 118 | 7 | -0.28 | 7.1 | 1.56 |
| 108 | 2 | 66 | 7.2 | 5.6 | 148 | 3 | -0.54 | 6.1 | 2.9 |
| 109 | 2 | 57 | 7.1 | 6.1 | 157 | 6 | 0.02 | 6.1 | 1.36 |
| 109 | 2 | 76 | 8.6 | 7.2 | 143 | 4 | 0.18 | 7 | 2.68 |
| 112 | 2 | 67 | 8.1 | 6.2 | 139 | 7 | -0.51 | 6.7 | 0.6 |
| 113 | 2 | 66 | 7.4 | 6.7 | 163 | 3 | 0.43 | 6.3 | 0.8 |
| 115 | 2 | 49 | 8.4 | 5.6 | 144 | 1 | -1.3 | 6.9 | 1.3 |
| 119 | 2 | 64 | 8.5 | 6.5 | 167 | 15 | -0.46 | 7 | 1.87 |
| 121 | 2 | 75 | 7.2 | 5.8 | 139 | 5 | -0.34 | 6.1 | 1.12 |
| 125 | 2 | 46 | 6.5 | 9 | 170 | 4 | 3.3 | 5.7 | 1.4 |
| 126 | 2 | 39 | 7.2 | 5.7 | 160 | 17 | -0.44 | 6.1 | 2.28 |
| 127 | 2 | 66 | 7.6 | 6.6 | 155 | 6 | 0.21 | 6.4 | 0.65 |
| 52 | 2 | 60 | 11.1 | 6.6 | 126 | 47 | -2.04 | 8.6 | 4.56 |
| 132 | 2 | 71 | 6.1 | 6.2 | 159 | 5 | 0.75 | 5.5 | 1.55 |
| 140 | 2 | 62 | 8 | 6.5 | 130 | 30 | -0.14 | 6.6 | 1.3 |
| 149 | 2 | 57 | 7.8 | 6.6 | 151 | 3 | 0.08 | 6.5 | 1.86 |
| 153 | 2 | 67 | 8.5 | 6.3 | 156 | 12 | -0.66 | 7 | 0.93 |
| 153 | 2 | 26 | 5.9 | 5.6 | 168 | 5 | 0.27 | 5.3 | 1.98 |
| 157 | 2 | 67 | 8.4 | 6.2 | 89 | 27 | -0.7 | 6.9 | 2.6 |
| 158 | 2 | 68 | 7.5 | 6.1 | 156 | 12 | -0.23 | 6.3 | 1.51 |
| 98 | 2 | 23 | 6.7 | 6.1 | 152 | 5 | 0.3 | 5.8 | 2.1 |
| 89 | 2 | 41 | 7.1 | 6.2 | 154 | 10 | 0.14 | 6.1 | 0.87 |
| 80 | 1 | 66 | 7.7 | 6.6 | 139 | 5 | 0.14 | 6.5 | 1.05 |
| 66 | 2 | 66 | 7.7 | 8.1 | 151 | 9 | 1.63 | 6.5 | 1.61 |
| 109 | 2 | 20 | 5.9 | 5.8 | 133 | 4 | 0.45 | 5.3 | 1.67 |

| TC | HDL | LDL | ALT | AST | SCr | BUN | Cardiovascular diseas | Fundus lesions | peripheral neuropathy |
|---|---|---|---|---|---|---|---|---|---|
| 6.34 | 1.41 | 4.47 | 18.1 | 17.9 | 69.6 | 6.69 | 0 | 0 | 0 |
| 4.15 | 1.31 | 2.62 | 19.7 | 19.3 | 71.5 | 5.2 | 0 | 0 | 0 |
| 3.71 | 1.1 | 2.14 | 12.2 | 17.2 | 67.2 | 4.09 | 0 | 0 | 0 |
| 5.15 | 1.11 | 3.66 | 14.7 | 24.6 | 75.5 | 5.08 | 0 | 0 | 0 |
| 7.25 | 1.12 | 5.22 | 20.1 | 14.1 | 342.9 | 22.78 | 0 | 0 | 0 |
| 5.92 | 1.15 | 3.54 | 32.5 | 19.9 | 73.2 | 5.25 | 0 | 0 | 0 |
| 4.1 | 1.2 | 2.7 | 12.9 | 15.6 | 72.1 | 4.13 | 0 | 0 | 0 |
| 6.27 | 1.06 | 3.66 | 9.8 | 15.7 | 122.7 | 8.38 | 0 | 0 | 0 |
| 4.14 | 1.07 | 2.83 | 19.4 | 31 | 69.6 | 3.72 | 0 | 0 | 0 |
| 5.66 | 1.56 | 3.8 | 16.4 | 16.2 | 69.8 | 5.8 | 0 | 0 | 0 |
| 4.85 | 0.8 | 3.51 | 17.4 | 16.7 | 79.1 | 4.96 | 0 | 0 | 0 |
| 5.39 | 1.17 | 3.68 | 25.6 | 15.9 | 62.2 | 6.05 | 0 | 0 | 0 |
| 3.54 | 0.53 | 1.77 | 20.2 | 24.9 | 95.1 | 6.48 | 0 | 0 | 0 |
| 4.38 | 1.08 | 3.1 | 36.6 | 35.4 | 84.7 | 8.2 | 0 | 0 | 0 |
| 4 | 1.06 | 2.38 | 35 | 44.5 | 66 | 5.88 | 1 | 0 | 1 |
| 7.33 | 0.73 | 4.89 | 52.6 | 28 | 69.3 | 4.81 | 1 | 1 | 1 |
| 4.87 | 1.15 | 2.72 | 38.1 | 16.7 | 95.5 | 7.9 | 1 | 0 | 0 |
| 5 | 0.9 | 3.6 | 32.9 | 23.2 | 70.7 | 5.25 | 0 | 0 | 0 |
| 3.67 | 0.82 | 2.45 | 35.2 | 23 | 60.6 | 4.48 | 0 | 0 | 0 |
| 4.64 | 1 | 3.29 | 37.4 | 29.7 | 94.3 | 8.22 | 1 | 1 | 1 |
| 6.23 | 1.47 | 4.3 | 23.3 | 19.8 | 68.6 | 6.09 | 0 | 0 | 0 |
| 5.73 | 1.33 | 4.01 | 14.1 | 21.6 | 71.8 | 4.3 | 1 | 0 | 0 |
| 3.84 | 0.92 | 2.57 | 18.3 | 20 | 35 | 4.1 | 0 | 0 | 0 |
| 4.84 | 1.43 | 3.1 | 15.9 | 19.7 | 105.5 | 7.27 | 1 | 0 | 0 |
| 6.7 | 1.24 | 4.66 | 34.9 | 28.5 | 62.8 | 3.7 | 1 | 1 | 1 |
| 4.06 | 1.18 | 2.51 | 25.3 | 20 | 57 | 3.84 | 0 | 0 | 0 |
| 3.81 | 0.95 | 2.69 | 20.9 | 19.1 | 52.5 | 4.47 | 0 | 0 | 0 |
| 3.12 | 0.68 | 2.18 | 12 | 16.2 | 76.8 | 5.6 | 0 | 0 | 0 |
| 3.4 | 0.9 | 2.4 | 26.4 | 20.5 | 57 | 4.8 | 0 | 0 | 0 |
| 3.91 | 1.17 | 2.58 | 11.3 | 17.8 | 61.8 | 3.9 | 0 | 0 | 0 |
| 5.3 | 1.8 | 3.1 | 13.8 | 19.5 | 54.2 | 4.93 | 0 | 0 | 0 |
| 4.96 | 1.19 | 3.33 | 27.8 | 19.4 | 69.3 | 7.1 | 0 | 0 | 0 |
| 4.6 | 1.2 | 3.1 | 41.7 | 28.3 | 63.5 | 5.37 | 1 | 1 | 1 |
| 6.85 | 1.6 | 4.49 | 117.2 | 75.2 | 73.6 | 4.2 | 0 | 1 | 0 |
| 6.27 | 1.01 | 3.67 | 19.2 | 21.9 | 52.7 | 3.8 | 1 | 0 | 0 |
| 6.34 | 1.76 | 3.8 | 46.1 | 31.4 | 58 | 7.91 | 0 | 0 | 0 |
| 5.82 | 1.4 | 3.89 | 94.1 | 45.6 | 46.9 | 4.5 | 1 | 1 | 0 |
| 4.02 | 1.14 | 2.57 | 12 | 18.1 | 60.5 | 6.3 | 0 | 0 | 0 |
| 5.98 | 1.75 | 3.71 | 10 | 20 | 45.5 | 4.93 | 0 | 0 | 0 |
| 5.01 | 1.8 | 2.51 | 24.1 | 22.4 | 50.1 | 5.32 | 0 | 0 | 0 |
| 6.2 | 1.4 | 4.1 | 16.6 | 21.1 | 185.9 | 12.74 | 0 | 0 | 0 |
| 3.85 | 1.18 | 2.45 | 65.1 | 37.6 | 53.3 | 5.03 | 0 | 0 | 1 |
| 6.39 | 1.18 | 4.45 | 19.7 | 21.8 | 58.7 | 4.12 | 0 | 0 | 0 |
| 8.3 | 1.2 | 6.2 | 41.8 | 23.8 | 78.1 | 6.6 | 1 | 1 | 0 |
| 4 | 1.1 | 2.6 | 18.2 | 21.4 | 56.6 | 5.4 | 0 | 0 | 0 |
| 3.1 | 0.9 | 0.1 | 22.2 | 17.2 | 43.4 | 3.6 | 1 | 0 | 0 |
| 5.02 | 1.3 | 3.41 | 42.9 | 20.1 | 53.5 | 4.28 | 0 | 0 | 0 |
| 4.2 | 1 | 3 | 69.8 | 43.6 | 82.5 | 7.12 | 1 | 0 | 0 |
| 5.37 | 1.51 | 3.7 | 16.7 | 17.7 | 58.2 | 6 | 0 | 0 | 0 |
| 4.56 | 2.09 | 2.39 | 18 | 26 | 44.7 | 2.83 | 1 | 1 | 0 |
| 4.81 | 1.07 | 3.34 | 16.3 | 18.2 | 58.9 | 7.2 | 0 | 0 | 0 |
| 5.03 | 1.23 | 3.44 | 22 | 22 | 62.2 | 5.2 | 0 | 0 | 0 |
| 5.63 | 2.07 | 3.26 | 22.5 | 21.6 | 163.6 | 10.05 | 0 | 1 | 0 |
| 6.15 | 1.19 | 4.28 | 33.2 | 26.9 | 48 | 6.93 | 0 | 0 | 0 |
| 4.99 | 1.18 | 3.47 | 17.8 | 19.7 | 47.8 | 6.42 | 0 | 0 | 0 |
| 5.42 | 1.09 | 3.64 | 24.2 | 23.1 | 53.8 | 3.99 | 0 | 0 | 0 |
| 5.12 | 1.54 | 3.36 | 10.4 | 11.3 | 49.2 | 6.41 | 0 | 0 | 0 |
| 4.1 | 1.3 | 2.5 | 14.7 | 21.2 | 72.2 | 5.7 | 0 | 0 | 0 |
| 5.71 | 0.93 | 2.6 | 38 | 23.8 | 4.3 | 61.1 | 0 | 0 | 0 |
| 5.22 | 1.61 | 3.18 | 10.8 | 14.4 | 63.4 | 6.26 | 0 | 0 | 0 |
| 4.79 | 1.66 | 2.91 | 23.2 | 19.1 | 50.5 | 7.38 | 1 | 1 | 1 |
| 4.28 | 1.3 | 2.72 | 23.3 | 18.7 | 48.8 | 5.41 | 0 | 0 | 0 |

| | | | | | | | | | |
|---|---|---|---|---|---|---|---|---|---|
| 3.97 | 0.98 | 2.75 | 23.6 | 18.3 | 47 | 3.9 | 0 | 0 | 0 |
| 5.45 | 1.07 | 3.9 | 18.7 | 17.9 | 65.9 | 4.95 | 0 | 0 | 0 |
| 4.87 | 1.22 | 3.36 | 15.7 | 42.8 | 52.4 | 5.94 | 0 | 0 | 0 |
| 4.9 | 1.12 | 3.2 | 12.8 | 14.2 | 59.1 | 4.75 | 1 | 0 | 1 |
| 4.07 | 1 | 2.64 | 20 | 18 | 56.3 | 5 | 0 | 0 | 0 |
| 3.9 | 1.3 | 2.4 | 26.1 | 23.1 | 69 | 5.88 | 0 | 1 | 0 |
| 4.1 | 1.2 | 2.5 | 15.2 | 16.7 | 43.9 | 4.52 | 0 | 0 | 1 |
| 4.15 | 1.31 | 2.66 | 28.9 | 24.9 | 41 | 4.54 | 0 | 0 | 0 |
| 6.31 | 1.26 | 4.64 | 10.8 | 15.9 | 43.3 | 5.13 | 0 | 0 | 0 |
| 3.7 | 1 | 2.5 | 12.3 | 10.2 | 54.5 | 4.5 | 0 | 0 | 0 |
| 3.9 | 0.9 | 2.7 | 51.2 | 34.9 | 49.6 | 3.9 | 0 | 0 | 0 |
| 4.9 | 1.3 | 3.2 | 34.5 | 29.7 | 57.3 | 4.16 | 0 | 0 | 0 |
| 7.04 | 1.14 | 5.27 | 29.3 | 23.1 | 57 | 3.5 | 0 | 0 | 0 |
| 3.24 | 1.15 | 1.98 | 26 | 17.7 | 39.1 | 4.48 | 0 | 0 | 0 |
| 5.84 | 1.02 | 4.29 | 26.2 | 22.7 | 47.2 | 5.71 | 0 | 0 | 0 |
| 6.75 | 1.3 | 4.56 | 10.8 | 12.9 | 78.5 | 7.53 | 0 | 1 | 0 |
| 5 | 1.17 | 3.28 | 25.6 | 22.2 | 89.7 | 7.91 | 1 | 1 | 1 |
| 4.6 | 1.7 | 2.8 | 18 | 17.4 | 50.3 | 4.1 | 1 | 1 | 1 |
| 3.5 | 0.9 | 2.3 | 10 | 15.2 | 52.5 | 5 | 1 | 1 | 1 |
| 3.9 | 0.9 | 1.8 | 15.4 | 16.3 | 53.7 | 3.42 | 0 | 0 | 0 |
| 5.93 | 1.66 | 3.71 | 47.8 | 33.9 | 73.8 | 3.87 | 0 | 0 | 0 |
| 4.09 | 1.01 | 2.61 | 14.1 | 17.6 | 94.6 | 5.61 | 0 | 0 | 0 |
| 6 | 1.4 | 4 | 11.3 | 18.9 | 69.2 | 5.69 | 0 | 0 | 0 |
| 2.46 | 0.72 | 1.6 | 27.7 | 25 | 69 | 2.8 | 0 | 0 | 0 |
| 4.77 | 1.31 | 3.26 | 17.3 | 19.3 | 77.2 | 6.17 | 0 | 0 | 0 |
| 5.34 | 1.33 | 2.33 | 22.1 | 26.5 | 93.1 | 5.17 | 1 | 1 | 1 |
| 5.14 | 1.04 | 3.84 | 17.9 | 16.8 | 70.6 | 5.78 | 1 | 1 | 1 |
| 4.52 | 1.03 | 3.1 | 36.4 | 33.7 | 78.8 | 4.23 | 0 | 0 | 0 |
| 4.35 | 0.96 | 3.18 | 45.4 | 25.6 | 61.2 | 6.08 | 0 | 0 | 0 |
| 4.57 | 1.47 | 2.83 | 7.9 | 10.1 | 745.9 | 20.69 | 0 | 0 | 0 |
| 4.8 | 1.26 | 3.13 | 28.1 | 29.4 | 69.8 | 5.78 | 0 | 0 | 0 |
| 6.9 | 1.3 | 4.8 | 23.8 | 17 | 67.4 | 5.18 | 0 | 0 | 1 |
| 5.76 | 1.23 | 4.14 | 24.3 | 28.4 | 93.4 | 8.58 | 0 | 0 | 0 |
| 5.01 | 1.47 | 3.13 | 23.6 | 40.1 | 49.7 | 5.36 | 1 | 1 | 1 |
| 6.37 | 1.88 | 4.19 | 8.6 | 18.3 | 52.8 | 5.74 | 1 | 1 | 1 |
| 4.31 | 1.26 | 2.77 | 15.1 | 18.9 | 58.6 | 4.93 | 1 | 1 | 0 |
| 4.89 | 1.4 | 3.05 | 28.6 | 31 | 73.9 | 6.02 | 1 | 0 | 1 |
| 5.59 | 1.31 | 3.78 | 13.5 | 15.7 | 57.2 | 3.9 | 1 | 0 | 1 |

| sex | age | course | ALT | AST | SCr | BUN | TC | TG | DL(mmol | DL(mmol | （mmol/ |
|---|---|---|---|---|---|---|---|---|---|---|---|
| 2 | 66 | 10 | 17.62 | 16.33 | 61.69 | 6.72 | 5.6 | 2.65 | 3.7 | 0.81 | 7.78 |
| 1 | 63 | 5 | 20.7 | 20.9 | 70 | 4.30 | 5.16 | 0.95 | 3.17 | 1.38 | 6.97 |
| 1 | 75 | 20 | 16.97 | 23.82 | 103.8 | 10.2 | 4.17 | 1.48 | 2.54 | 1.00 | 4.51 |
| 1 | 47 | 5 | 20.9 | 16.1 | 49 | 2.94 | 4.33 | 6.26 | 2.10 | 0.88 | 7.78 |
| 2 | 61 | 14 | 29.72 | 20.96 | 55.9 | 4.7 | 6.69 | 2.56 | 4.33 | 1.62 | 11.16 |
| 1 | 68 | 1 | 6.78 | 14.63 | 87.01 | 5.26 | 3.86 | 0.58 | 2.39 | 1.23 | 5.45 |
| 2 | 65 | 15 | 68.7 | 43.1 | 60 | 5.08 | 6.05 | 1.86 | 3.77 | 0.98 | 8.01 |
| 1 | 67 | 10 | 17 | 18 | 65.9 | 7.9 | 3.10 | 0.69 | 1.53 | 0.89 | 6.13 |
| 1 | 66 | 20 | 30 | 20 | 87.1 | 6.04 | 4.8 | 2.96 | 2.49 | 1.3 | 12.47 |
| 1 | 63 | 5 | 25.522 | 34.912 | 64.256 | 5.284 | 3.644 | 1.484 | 1.704 | 1.136 | 6.184 |
| 2 | 64 | 15 | 39.65 | 27.31 | 49.9 | 4.5 | 5.02 | 1.37 | 3.27 | 1.09 | 6.62 |
| 2 | 65 | 15 | 21.1 | 18.7 | 45 | 3.10 | 4.33 | 1.67 | 2.18 | 1.54 | 6.99 |
| 1 | 64 | 10 | 11.78 | 14.69 | 90.7 | 5.13 | 3.41 | 1.43 | 1.98 | 1.13 | 7.24 |
| 1 | 67 | 3 | 24.72 | 14.32 | 50.61 | 3.63 | 6.6 | 3.52 | 4.81 | 0.93 | 13.62 |
| 1 | 72 | 12 | 13.88 | 16.49 | 71.05 | 6 | 5.9 | 1.66 | 4.11 | 1.13 | 7.42 |
| 2 | 64 | 7 | 18 | 18 | 59.2 | 4.3 | 7.86 | 1.71 | 5.16 | 2.00 | 8.44 |
| 1 | 64 | 12 | 13.45 | 17.03 | 59.2 | 5.6 | 4.82 | 0.83 | 2.85 | 1.58 | 6.61 |
| 1 | 66 | 20 | 34.33 | 27.36 | 70.6 | 6.5 | 5.43 | 0.84 | 3.22 | 1.63 | 12.53 |
| 2 | 64 | 6 | 13.4 | 14.0 | 51.23 | 4.26 | 5.17 | 7.07 | 2.44 | 1.18 | 6.78 |
| 2 | 67 | 15 | 13.34 | 12.56 | 64.21 | 4.27 | 5.99 | 2.48 | 3.78 | 1.18 | 12.42 |
| 1 | 69 | 2 | 1.7 | 14.8 | 87.22 | 6.16 | 6.40 | 1.47 | 3.61 | 2.31 | 8.65 |
| 2 | 70 | 4 | 19.61 | 19.73 | 46.99 | 5.36 | 3.18 | 1.81 | 1.44 | 1.46 | 6.97 |
| 1 | 65 | 5 | 11.19 | 12.24 | 111.03 | 6.89 | 6.87 | 2.23 | 4.97 | 1.04 | 12.07 |
| 2 | 72 | 10 | 43.1 | 26.8 | 53 | 5.00 | 5.34 | 2.42 | 3.10 | 1.29 | 7.28 |
| 1 | 60 | 8 | 16.2 | 13.9 | 67.53 | 6.88 | 4.04 | 1.00 | 2.42 | 1.18 | 6.5 |
| 1 | 70 | 1 | 19.7 | 22.0 | 57 | 4.99 | 6.65 | 1.05 | 4.22 | 1.53 | 6.54 |
| 1 | 71 | 2 | 20.55 | 16.06 | 97.45 | 5.3 | 3.88 | 0.94 | 2.17 | 1.34 | 7.5 |
| 2 | 62 | 3 | 12.1 | 12.4 | 54 | 3.32 | 5.21 | 1.13 | 3.25 | 1.08 | 4.92 |
| 2 | 63 | 30 | 13.6 | 13.99 | 53.21 | 7.42 | 4.97 | 2.74 | 3.28 | 1 | 8.97 |
| 1 | 70 | 11 | 15.0 | 20.0 | 71.47 | 6.82 | 4.21 | 0.89 | 1.88 | 1.63 | 6.23 |
| 2 | 70 | 12 | 26.81 | 17.1 | 42.00 | 5.4 | 6.82 | 1.24 | 3.87 | 2.38 | 8.33 |
| 1 | 63 | 19 | 20 | 17 | 49.8 | 5.24 | 6.57 | 3.30 | 4.25 | 1.42 | 8.41 |
| 2 | 77 | 1 | 7.70 | 11 | 58.4 | 4.85 | 6.7 | 4.15 | 0.71 | 2.39 | 1.22 |
| 1 | 69 | 7 | 15.90 | 11.11 | 72.7 | 5.5 | 4.32 | 1.09 | 2.36 | 1.46 | 7.07 |
| 2 | 55 | 2 | 32.19 | 20.23 | 49.35 | 5.88 | 3.81 | 1.50 | 1.82 | 1.26 | 8.23 |
| 1 | 80 | 3 | 27.87 | 19.03 | 56.98 | 4.92 | 7.23 | 2.17 | 5.12 | 1.27 | 13.59 |
| 2 | 63 | 10 | 22.2 | 23 | 41.06 | 3.37 | 3.28 | 0.83 | 1.43 | 1.58 | 6.52 |
| 1 | 69 | 23 | 21.21 | 20.52 | 54.36 | 4.75 | 6.36 | 1.94 | 4.18 | 1.72 | 6.34 |
| 2 | 72 | 10 | 13 | 16 | 70.7 | 5.79 | 4.97 | 1.23 | 3.05 | 1.42 | 10.69 |
| 1 | 69 | 3 | 15.7 | 17.8 | 64 | 4.72 | 3.76 | 0.94 | 2.27 | 1.08 | 6.93 |
| 2 | 69 | 8 | 20.57 | 24.12 | 38.04 | 5.6 | 3.75 | 1.96 | 2.2 | 1.1 | 32.1 |
| 1 | 68 | 3 | 27.4 | 17.3 | 97 | 5.20 | 3.88 | 3.49 | 2.17 | 0.76 | 5.27 |
| 2 | 66 | 7 | 6.66 | 18.32 | 50.6 | 4.3 | 5.96 | 1.35 | 4.56 | 1.51 | 8.66 |
| 2 | 58 | 3 | 24.14 | 17.63 | 53.0 | 6.1 | 5.25 | 1.28 | 3.36 | 1.29 | 7.87 |
| 1 | 72 | 5 | 20.2 | 29.13 | 70.82 | 5.77 | 4.86 | 1.12 | 3.35 | 1.09 | 32.9 |
| 2 | 68 | 10 | 13.62 | 10.95 | 53.7 | 7.4 | 3.31 | 2.05 | 1.54 | 1.30 | 8.61 |
| 2 | 73 | 7 | 11.21 | 14.71 | 73.7 | 5.2 | 4.75 | 1.10 | 3.27 | 0.84 | 6.42 |

| | | | | | | | | | | | |
|---|---|---|---|---|---|---|---|---|---|---|---|
| 2 | 61 | 10 | 13.0 | 14.4 | 109 | 3.90 | 5.17 | 2.58 | 2.78 | 1.03 | 8.13 |
| 2 | 65 | 2 | 26.0 | 22.8 | 56 | 3.28 | 6.67 | 0.92 | 3.79 | 2.17 | 6.75 |
| 1 | 70 | 26 | 17.75 | 16.68 | 54.46 | 3.82 | 4.02 | 1.05 | 2.56 | 1.16 | 14.45 |
| 1 | 61 | 3 | 37.3 | 25.3 | 68 | 4.49 | 3.80 | 0.96 | 2.18 | 1.00 | 4.98 |
| 2 | 62 | 8 | 21.58 | 31.19 | 47.84 | 6.11 | 3.92 | 1.7 | 2.44 | 1.15 | 8.17 |
| 1 | 68 | 20 | 19.6 | 16.7 | 68 | 6.52 | 2.88 | 0.84 | 1.48 | 1.15 | 8.43 |
| 2 | 73 | 1 | 19.69 | 20.88 | 57.0 | 6.0 | 5.45 | 1.46 | 3.14 | 1.57 | 5.84 |
| 1 | 70 | 20 | 14.97 | 14.80 | 75.3 | 5.4 | 4.70 | 1.28 | 2.79 | 1.24 | 6.61 |
| 2 | 70 | 20 | 16 | 14 | 47.3 | 7.5 | 5.45 | 2.31 | 3.39 | 1.20 | 10.36 |
| 2 | 69 | 3 | 16 | 16 | 55.4 | 3.4 | 6.35 | 1.71 | 4.22 | 1.59 | 6.2 |
| 2 | 70 | 2 | 14.1 | 15.5 | 62 | 6.87 | 4.61 | 2.98 | 2.32 | 1.45 | 5.85 |
| 2 | 66 | 8 | 15.7 | 16.6 | 54 | 5.88 | 5.47 | 1.39 | 3.00 | 1.81 | 7.52 |
| 1 | 71 | 10 | 25.19 | 17.36 | 91.76 | 8.30 | 3.91 | 0.78 | 2.43 | 1.17 | 5.63 |
| 1 | 69 | 1 | 25.75 | 18.09 | 60.14 | 7.69 | 5.22 | 1.92 | 3.69 | 1.10 | 11.41 |
| 2 | 72 | 20 | 17 | 17 | 55.3 | 5.3 | 7.10 | 1.21 | 4.82 | 1.36 | 14.78 |
| 2 | 69 | 15 | 17.0 | 15.9 | 46 | 5.88 | 6.16 | 1.10 | 3.86 | 1.48 | 16.13 |
| 1 | 65 | 1 | 19.1 | 18.1 | 77 | 4.38 | 3.07 | 0.97 | 1.57 | 1.12 | 6.09 |
| 1 | 73 | 23 | 25 | 22 | 84.2 | 7.6 | 5.00 | 0.89 | 3.29 | 1.07 | 7.95 |
| 2 | 66 | 1 | 23.48 | 24.29 | 53.6 | 5.8 | 4.89 | 4.95 | 2.35 | 1.04 | 7.08 |
| 2 | 66 | 9 | 15.2 | 18.9 | 41.6 | 4.52 | 4.19 | 0.95 | 2.08 | 1.53 | 7.03 |
| 2 | 58 | 15 | 14.97 | 15.30 | 55.86 | 6.63 | 5.33 | 0.77 | 3.00 | 1.48 | 4.73 |
| 2 | 65 | 2 | 17.9 | 13.8 | 55 | 4.49 | 4.48 | 1.06 | 2.20 | 1.52 | 5.81 |
| 1 | 61 | 25 | 9.83 | 14.38 | 74.93 | 6.09 | 3.75 | 1.66 | 2.44 | 0.9 | 8.17 |
| 2 | 70 | 10 | 22.7 | 15.3 | 73.74 | 5.46 | 6.49 | 1.76 | 5.07 | 1.39 | 7.31 |
| 1 | 78 | 10 | 22 | 18 | 139.6 | 15.9 | 5.54 | 3.22 | 3.38 | 0.93 | 17.37 |
| 2 | 59 | 4 | 32.86 | 25.44 | 63.5 | 8.5 | 5.87 | 2.38 | 3.51 | 1.28 | 6.52 |
| 1 | 65 | 9 | 57.8 | 53.3 | 86.54 | 4.68 | 4.10 | 1.90 | 2.05 | 1.03 | 7.73 |
| 1 | 74 | 10 | 10.86 | 16.18 | 81.0 | 6.2 | 6.05 | 0.84 | 4.20 | 1.31 | 5.11 |
| 2 | 68 | 16 | 16.99 | 14.35 | 42.00 | 8.37 | 5.51 | 2.35 | 3.76 | 1.04 | 11.04 |
| 2 | 64 | 15 | 12.28 | 11.81 | 96.6 | 6.6 | 5.07 | 3.18 | 3.01 | 0.88 | 9 |
| 1 | 53 | 2 | 58.62 | 104.91 | 72.3 | 4.0 | 6.42 | 3.83 | 3.46 | 1.75 | 8.01 |
| 1 | 73 | 20 | 92.97 | 85.51 | 50.14 | 5.87 | 4.27 | 1.3 | 2.4 | 1.48 | 8.9 |
| 2 | 68 | 3 | 22.9 | 27 | 81.3 | 6.98 | 6.4 | 3.04 | 3.69 | 1.53 | 7.26 |
| 2 | 64 | 14 | 36.12 | 43.78 | 57.5 | 7.26 | 3.94 | 1.19 | 1.14 | 2.63 | 15.95 |
| 1 | 61 | 2 | 52.82 | 35.84 | 42.33 | 3.97 | 5.6 | 1.97 | 3.41 | 1.43 | 6.92 |
| 2 | 64 | 4 | 24.64 | 19.71 | 53.41 | 4.93 | 3.94 | 3.99 | 2.51 | 0.71 | 12.23 |
| 2 | 68 | 2 | 19 | 18 | 47.2 | 2.9 | 3.96 | 3.12 | 2.24 | 0.96 | 7.33 |
| 2 | 72 | 1 | 30 | 25 | 56.1 | 4.2 | 3.86 | 1.76 | 1.95 | 1.19 | 6.71 |
| 2 | 60 | 20 | 10.77 | 13.12 | 56.42 | 5.59 | 4.44 | 1 | 2.82 | 1.25 | 9.92 |
| 1 | 70 | 8 | 121.5 | 91.44 | 82.89 | 7.97 | 6.46 | 4.24 | 3.86 | 0.95 | 12.59 |
| 1 | 66 | 8 | 39.51 | 26.63 | 154.7 | 15.3 | 5.02 | 0.74 | 2.8 | 1.74 | 7.86 |
| 2 | 76 | 4 | 18 | 18 | 60.2 | 4.2 | 6.19 | 1.65 | 3.41 | 2.32 | 11.46 |
| 2 | 61 | 20 | 17.9 | 25.3 | 99 | 8.83 | 4.35 | 0.88 | 2.03 | 2.06 | 14.56 |
| 1 | 68 | 6 | 47 | 37 | 57.4 | 5.7 | 5.84 | 1.06 | 3.89 | 1.36 | 6.96 |
| 1 | 63 | 20 | 16 | 13 | 87.3 | 9.2 | 9.14 | 1.56 | 6.48 | 1.24 | 10.93 |
| 2 | 73 | 20 | 26.78 | 25.62 | 56.38 | 4.82 | 5.41 | 2.46 | 3.53 | 1.38 | 9.34 |
| 2 | 63 | 28 | 38.31 | 23.69 | 59.41 | 4.61 | 5.31 | 3.17 | 3.27 | 0.98 | 6.03 |
| 2 | 77 | 20 | 48.2 | 35.9 | 90 | 10.26 | 5.51 | 1.71 | 3.39 | 0.9 | 9.57 |

| | | | | | | | | | | | |
|---|---|---|---|---|---|---|---|---|---|---|---|
| 1 | 67 | 3 | 16.1 | 15.3 | 89.3 | 4.88 | 5.47 | 2.14 | 3.37 | 1.37 | 7.64 |
| 2 | 70 | 14 | 19.5 | 14 | 55.55 | 7.83 | 5.01 | 1.97 | 3.16 | 1.38 | 12.11 |
| 2 | 66 | 3 | 34.9 | 25.9 | 47.56 | 4.88 | 5.94 | 2.33 | 4.33 | 1.16 | 6.7 |
| 2 | 66 | 5 | 19.3 | 20.4 | 53.18 | 4.02 | 6.06 | 1.63 | 4.06 | 1.66 | 7.62 |
| 1 | 68 | 3 | 58.13 | 55.62 | 82.85 | 6.32 | 4.26 | 2.31 | 2.15 | 1.22 | 7.42 |
| 1 | 75 | 10 | 19.7 | 21.47 | 66.16 | 6.06 | 4.61 | 5.1 | 2.48 | 0.82 | 7.88 |
| 1 | 65 | 20 | 26.5 | 20 | 91 | 5.93 | 3.82 | 1.85 | 2.03 | 0.84 | 10.97 |
| 1 | 79 | 1 | 21.6 | 17.9 | 76 | 5.97 | 4.13 | 1.14 | 1.8 | 1.21 | 6.99 |
| 2 | 69 | 20 | 12.78 | 12.62 | 66.9 | 5.5 | 6.36 | 1.32 | 4.21 | 1.40 | 7.25 |
| 2 | 63 | 11 | 15 | 16 | 57.4 | 5.1 | 5.72 | 2.74 | 3.66 | 1.25 | 8.37 |
| 1 | 68 | 7 | 13 | 17 | 76.5 | 5.5 | 3.73 | 1.58 | 2.46 | 0.94 | 8.06 |
| 2 | 67 | 18 | 27.07 | 20.01 | 50.55 | 4.61 | 4.27 | 0.98 | 2.22 | 1.83 | 6.82 |
| 2 | 70 | 10 | 70.64 | 84.66 | 58.81 | 3.96 | 6.02 | 1.63 | 4.05 | 1.45 | 12.77 |
| 2 | 64 | 15 | 19 | 15 | 54.6 | 5.6 | 3.92 | 0.89 | 2.29 | 1.48 | 6.46 |
| 1 | 71 | 20 | 17.84 | 19.25 | 81.71 | 11.43 | 5.8 | 1.3 | 3.36 | 1.37 | 8.1 |
| 1 | 72 | 20 | 12.39 | 13.59 | 59.3 | 6.9 | 3.47 | 0.91 | 1.84 | 1.22 | 7.38 |
| 2 | 65 | 7 | 27 | 21 | 55.6 | 5.7 | 6.51 | 2.34 | 4.26 | 1.13 | 8.18 |
| 1 | 68 | 3 | 17.54 | 17.62 | 67.33 | 7.07 | 4.95 | 0.62 | 2.81 | 1.36 | 6.42 |
| 2 | 67 | 3 | 16.69 | 15.8 | 50.5 | 4.8 | 5.2 | 1.88 | 2.68 | 1.96 | 8.25 |
| 1 | 64 | 10 | 19.73 | 15.45 | 72.8 | 6.7 | 3.2 | 1.08 | 1.87 | 0.92 | 6.04 |
| 1 | 70 | 3 | 15.2 | 14.3 | 51 | 4.84 | 5.17 | 0.96 | 2.66 | 1.75 | 7.63 |
| 1 | 67 | 15 | 21.79 | 20.36 | 126.19 | 7.52 | 4.42 | 1.84 | 2.59 | 1.23 | 7.67 |
| 2 | 74 | 10 | 19.32 | 24.42 | 65.5 | 6.2 | 5.2 | 1.29 | 3.22 | 1.4 | 5.86 |
| 1 | 67 | 20 | 16.6 | 13.1 | 89 | 4.46 | 4.5 | 2.61 | 2.2 | 1.08 | 7.4 |
| 1 | 69 | 10 | 30.4 | 18.8 | 62.39 | 5.97 | 6.44 | 1.17 | 3.58 | 1.7 | 7.07 |
| 2 | 61 | 16 | 30.1 | 24.6 | 44 | 7.55 | 4.06 | 0.98 | 1.77 | 1.77 | 7.57 |
| 2 | 65 | 5 | 22 | 21 | 39.8 | 5.6 | 5.15 | 2.22 | 3.72 | 0.97 | 7.6 |
| 2 | 71 | 6 | 14 | 22 | 56.8 | 4.1 | 5.76 | 1.78 | 3.85 | 1.3 | 6.1 |
| 1 | 64 | 3 | 44.9 | 35.4 | 81.68 | 6.74 | 4.65 | 1.27 | 2.67 | 1.25 | 6.85 |
| 1 | 65 | 20 | 16 | 14 | 66.5 | 6 | 5.23 | 0.78 | 3.71 | 1.27 | 6 |
| 1 | 65 | 3 | 19.32 | 14.98 | 79.7 | 4.1 | 3.95 | 2.17 | 2.4 | 0.85 | 7 |
| 2 | 68 | 14 | 11.09 | 16.66 | 64.02 | 6.74 | 5.28 | 1.66 | 3.32 | 1.45 | 6.51 |
| 2 | 78 | 16 | 13 | 20 | 57.5 | 6.9 | 6.08 | 1.01 | 4.46 | 1.1 | 7.08 |
| 2 | 60 | 7 | 16.9 | 14.6 | 51 | 4.43 | 5.01 | 2.21 | 3.27 | 0.76 | 8.41 |
| 1 | 65 | 8 | 23.02 | 22.47 | 63.2 | 7.3 | 3.66 | 2.84 | 2.18 | 0.94 | 8.54 |
| 1 | 61 | 6 | 29.98 | 25.14 | 58.48 | 7.22 | 2.97 | 1.1 | 1.51 | 1.26 | 10.7 |
| 2 | 66 | 8 | 21 | 23 | 42.9 | 2.9 | 6.35 | 3.34 | 4.43 | 0.72 | 7.34 |
| 2 | 61 | 6 | 20.7 | 17.3 | 41.07 | 2.86 | 7.07 | 4.94 | 4.22 | 1.64 | 8.98 |
| 2 | 58 | 15 | 24.3 | 18.1 | 47 | 6.45 | 6.03 | 1.43 | 3.69 | 1.21 | 7.25 |
| 2 | 75 | 7 | 11 | 17 | 86.1 | 8.3 | 4.03 | 1.32 | 1.91 | 1.65 | 6.07 |
| 1 | 70 | 25 | 7.91 | 10.39 | 125.52 | 10.43 | 4.97 | 2.1 | 3.13 | 1.3 | 14.76 |
| 2 | 63 | 7 | 48.67 | 32.86 | 55.26 | 5.82 | 5.67 | 2.3 | 3.58 | 1.42 | 7.58 |
| 1 | 66 | 10 | 17 | 21 | 159.8 | 11.5 | 4.41 | 3.74 | 2.2 | 0.79 | 7.19 |
| 1 | 75 | 10 | 24.2 | 16 | 48.9 | 4.05 | 3.8 | 1.06 | 1.72 | 1.72 | 11.21 |
| 2 | 71 | 6 | 17 | 20 | 48.6 | 7.4 | 4.86 | 0.62 | 2.12 | 2.27 | 7.99 |
| 1 | 70 | 4 | 20.27 | 18.07 | 85.24 | 7.34 | 4.58 | 2.56 | 3.11 | 1.08 | 8.53 |

| | | | | | | | | | | | |
|---|---|---|---|---|---|---|---|---|---|---|---|
| 1 | 75 | 10 | 21.9 | 22.2 | 58.5 | 7.1 | 4.51 | 0.67 | 2.83 | 1.39 | 9.9 |
| 2 | 66 | 14 | 52 | 44 | 51 | 4.8 | 5.64 | 2.7 | 3.66 | 1.11 | 8.13 |
| 2 | 74 | 20 | 15.7 | 14.1 | 66 | 4.57 | 5.91 | 1.61 | 3.36 | 1.41 | 6.73 |
| 1 | 60 | 23 | 18.89 | 16.93 | 58.21 | 5.66 | 5.56 | 1.36 | 3.75 | 1.42 | 7.28 |
| 2 | 66 | 23 | 9.9 | 11.1 | 64.77 | 6.58 | 5.63 | 7.94 | 2.59 | 1.16 | 9.09 |
| 2 | 62 | 2 | 13.6 | 13.3 | 54 | 4.96 | 4.26 | 1.03 | 2.64 | 1.17 | 4.95 |
| 1 | 66 | 10 | 27 | 24 | 79.7 | 5.32 | 5.74 | 2.07 | 3.2 | 1.77 | 6.02 |
| 1 | 61 | 30 | 17.47 | 14.94 | 54.8 | 5.8 | 5.74 | 0.53 | 3.45 | 1.87 | 11.16 |
| 2 | 66 | 10 | 50 | 26.7 | 57 | 4.59 | 5.2 | 1.88 | 2.7 | 1.44 | 8.59 |
| 2 | 71 | 20 | 22.71 | 18.36 | 49.99 | 6.3 | 5.88 | 1.38 | 3.58 | 1.25 | 10.41 |
| 2 | 65 | 12 | 20.9 | 15.9 | 52 | 5.11 | 2.87 | 1.2 | 1.35 | 1.11 | 8.17 |
| 2 | 79 | 11 | 9 | 17 | 92.9 | 6.9 | 5.67 | 1.28 | 3.35 | 1.54 | 5.67 |
| 1 | 61 | 16 | 24 | 13 | 79.4 | 4.66 | 4.21 | 1.11 | 2.47 | 1.29 | 15.16 |
| 2 | 60 | 15 | 17.5 | 17.8 | 53 | 7.01 | 5.22 | 0.96 | 2.78 | 1.85 | 7.37 |
| 2 | 70 | 11 | 23.9 | 17 | 57 | 6.4 | 5.4 | 0.99 | 3.2 | 1.58 | 5.72 |
| 2 | 74 | 6 | 28 | 24 | 61.4 | 5.6 | 4.15 | 1.85 | 2.48 | 1 | 9.01 |
| 2 | 73 | 20 | 29 | 29 | 51.3 | 7.5 | 4.76 | 0.64 | 2.15 | 2.1 | 5.93 |
| 1 | 73 | 20 | 13 | 14.6 | 63 | 5.88 | 4.18 | 1.04 | 2.36 | 1.29 | 6.55 |
| 2 | 57 | 10 | 44.9 | 24.9 | 44.1 | 5.36 | 5.59 | 1.41 | 3.41 | 1.5 | 7.91 |
| 2 | 63 | 10 | 24 | 21 | 42.2 | 3.9 | 5.92 | 2.24 | 3.76 | 1.49 | 9.05 |
| 1 | 60 | 7 | 16.37 | 21.36 | 54.96 | 4.79 | 4.69 | 1.1 | 2.62 | 1.75 | 8.1 |
| 2 | 59 | 10 | 29.372 | 25.266 | 54.904 | 23.364 | 4.658 | 2.59 | 2.696 | 1.082 | 9.452 |
| 2 | 68 | 11 | 23.5 | 18.2 | 55.25 | 6.97 | 4.68 | 1.8 | 2.87 | 1.04 | 8.05 |
| 2 | 60 | 6 | 19.99 | 16.88 | 39.3 | 3.93 | 8.37 | 1.59 | 4.99 | 1.59 | 12.91 |

| Hb (gL) | CO | RBCs | hba1c | Monitoring days | HGI=lab-eHbA1c |
|---|---|---|---|---|---|
| 116 | 2.1151 | 76 | 6.9 | 7 | 0.92 |
| 138 | 2.2916 | 83 | 6.4 | 15 | 0.30 |
| 149 | 3.0264 | 68 | 6.8 | 15 | -0.12 |
| 156 | 3.0126 | 71 | 7.6 | 12 | 0.78 |
| 142 | 2.9515 | 66 | 6.3 | 3 | 0.06 |
| 154 | 3.1934 | 67 | 8.9 | 8 | 2.38 |
| 129 | 2.8176 | 63 | 6.8 | 15 | 0.84 |
| 156 | 2.8477 | 76 | 7.7 | 15 | 0.31 |
| 164 | 2.0645 | 110 | 9.4 | 8 | 1.34 |
| 137 | 1.956 | 97 | 6.8 | 15 | 0.73 |
| 151 | 2.1318 | 9 | 6.8 | 3 | 1.08 |
| 127 | 3.9439 | 44 | 6.4 | 15 | 0.37 |
| 152 | 8.4809 | 25 | 7.4 | 15 | 1.01 |
| 151 | 2.5322 | 82 | 9.2 | 15 | 2.74 |
| 139 | 2.6961 | 71 | 6.5 | 4 | 0.28 |
| 129 | 2.2272 | 80 | 6.8 | 13 | 0.33 |
| 145 | 3.0925 | 65 | 6.6 | 15 | 0.58 |
| 163 | 3.2321 | 70 | 6.7 | 15 | 0.16 |
| 131 | 5.6176 | 32 | 6.9 | 5 | 0.60 |
| 145 | 3.0438 | 66 | 8.2 | 9 | 0.89 |
| 171 | 3.0502 | 77 | 6 | 15 | 0.18 |
| 140 | 3.9713 | 49 | 7.7 | 5 | 1.79 |
| 144 | 2.1346 | 93 | 7.2 | 8 | 0.25 |
| 111 | 2.3537 | 65 | 6 | 14 | 0.78 |
| 165 | 3.6492 | 62 | 8.5 | 14 | 1.88 |
| 151 | 3.5895 | 58 | 5.5 | 15 | -0.16 |
| 129 | 3.7567 | 47 | 6.9 | 5 | 0.17 |
| 120 | 2.1584 | 77 | 6.2 | 11 | 0.38 |
| 131 | 4.0171 | 45 | 8.1 | 15 | 0.86 |
| 149 | 3.0467 | 67 | 7.8 | 5 | 0.95 |
| 129 | 1.5845 | 112 | 7.4 | 15 | 0.49 |
| 135 | 2.086 | 89 | 7.8 | 9 | 0.19 |
| 123 | 1.2868 | 52 | 8.3 | 15 | 1.81 |
| 162 | 4.0008 | 56 | 5.3 | 8 | -0.50 |
| 145 | 1.7177 | 42 | 9.5 | 10 | 3.07 |
| 155 | 3.1783 | 67 | 7.7 | 15 | 0.53 |
| 142 | 2.7758 | 71 | 8.4 | 15 | 2.33 |
| 129 | 2.4079 | 74 | 6.6 | 9 | 0.58 |
| 143 | 3.147 | 63 | 9.4 | 15 | 0.58 |
| 141 | 3.2288 | 60 | 6.6 | 15 | 0.36 |
| 150 | 2.8498 | 73 | 6.4 | 9 | 0.35 |
| 154 | 3.0413 | 70 | 5 | 12 | -1.16 |
| 153 | 3.4152 | 62 | 7.2 | 13 | 1.22 |
| 147 | 3.737 | 54 | 7.4 | 15 | 0.34 |
| 127 | 1.521 | 115 | 8.8 | 15 | 2.89 |
| 133 | 4.6042 | 40 | 7.5 | 8 | 1.19 |
| 130 | 2.8378 | 63 | 6.3 | 11 | 0.31 |

| | | | | | |
|---|---|---|---|---|---|
| 131 | 1.9389 | 62 | 6.7 | 15 | 0.30 |
| 149 | 2.639 | 78 | 6.2 | 15 | 0.07 |
| 134 | 3.2617 | 57 | 7.9 | 13 | -0.71 |
| 129 | 3.238 | 55 | 5.3 | 7 | -0.62 |
| 112 | 1.2428 | 124 | 8.1 | 10 | 2.14 |
| 133 | 2.479 | 74 | 6.7 | 15 | 0.58 |
| 137 | 3.4609 | 55 | 6.9 | 4 | 1.45 |
| 191 | 3.0215 | 87 | 5.5 | 13 | -0.58 |
| 114 | 2.8999 | 54 | 7.6 | 15 | 0.59 |
| 146 | 9.3718 | 21 | 6.1 | 13 | 0.05 |
| 127 | 2.2493 | 78 | 6.6 | 15 | 0.46 |
| 146 | 2.6583 | 76 | 7.1 | 15 | 0.70 |
| 169 | 3.9619 | 59 | 6.3 | 8 | -0.01 |
| 162 | 2.8081 | 80 | 7.3 | 13 | -1.56 |
| 135 | 2.9252 | 64 | 10.2 | 15 | 1.19 |
| 129 | 2.2472 | 79 | 11.7 | 10 | 2.97 |
| 125 | 7.0687 | 24 | 6 | 15 | -0.03 |
| 158 | 3.0875 | 71 | 7.3 | 15 | 1.39 |
| 137 | 3.0966 | 61 | 6.4 | 9 | 0.39 |
| 132 | 2.4128 | 75 | 6.4 | 8 | -0.09 |
| 133 | 2.1441 | 58 | 8.3 | 12 | 1.86 |
| 132 | 3.0469 | 60 | 5.6 | 15 | -0.01 |
| 129 | 14.6739 | 12 | 7.2 | 10 | 1.13 |
| 132 | 2.6687 | 68 | 9 | 15 | 2.83 |
| 119 | 2.4022 | 68 | 7.2 | 6 | 1.07 |
| 128 | 2.1019 | 57 | 7.8 | 2 | 1.28 |
| 153 | 2.8108 | 27 | 7.4 | 12 | 0.41 |
| 163 | 1.4677 | 65 | 6.8 | 15 | 0.28 |
| 130 | 1.8153 | 99 | 9.2 | 12 | 0.98 |
| 124 | 11.3693 | 15 | 5.6 | 15 | -0.46 |
| 153 | 1.4018 | 16 | 6.8 | 15 | 0.37 |
| 124 | 3.3722 | 51 | 7.5 | 14 | 0.56 |
| 113 | 2.1699 | 72 | 7.3 | 8 | 1.53 |
| 150 | 2.0237 | 102 | 11.5 | 15 | 3.48 |
| 120 | 2.4294 | 68 | 6.5 | 15 | 0.48 |
| 141 | 3.0263 | 64 | 7.8 | 9 | 1.68 |
| 138 | 2.6099 | 73 | 7.1 | 5 | -0.51 |
| 128 | 2.4833 | 71 | 7.5 | 5 | 1.20 |
| 130 | 2.006 | 89 | 8.1 | 7 | 1.01 |
| 164 | 2.7502 | 82 | 7.5 | 15 | 0.38 |
| 125 | 2.39 | 72 | 6.5 | 15 | -0.36 |
| 116 | 2.2848 | 37 | 4 | 15 | -2.44 |
| 106 | 2.8646 | 51 | 6.6 | 15 | 0.50 |
| 145 | 2.8764 | 70 | 6.5 | 8 | 0.79 |
| 145 | 2.9734 | 67 | 8.5 | 15 | 0.42 |
| 115 | 2.2525 | 70 | 5.8 | 14 | -0.18 |
| 128 | 1.6364 | 108 | 9.8 | 7 | 1.41 |
| 141 | 3.1733 | 61 | 7.1 | 15 | 0.17 |

| | | | | | |
|---|---|---|---|---|---|
| 177 | 4.4719 | 55 | 7.1 | 15 | 1.06 |
| 146 | 2.8396 | 71 | 7.8 | 15 | 1.46 |
| 133 | 2.9405 | 62 | 6.6 | 10 | 0.63 |
| 154 | 3.0668 | 69 | 7.4 | 15 | 1.04 |
| 141 | 1.6264 | 120 | 7.4 | 15 | 0.00 |
| 160 | 2.7878 | 79 | 7.2 | 10 | 1.65 |
| 141 | 2.8326 | 69 | 6 | 6 | 0.31 |
| 146 | 2.1958 | 33 | 8.1 | 15 | 1.32 |
| 143 | 2.9837 | 66 | 7.8 | 7 | 2.03 |
| 118 | 2.0837 | 78 | 6.5 | 13 | 0.04 |
| 158 | 2.9726 | 73 | 6.1 | 10 | -0.08 |
| 130 | 3.1561 | 57 | 6.3 | 14 | 0.25 |
| 134 | 1.2914 | 143 | 8.8 | 15 | 0.46 |
| 129 | 2.5789 | 69 | 7.1 | 6 | -0.27 |
| 142 | 3.0109 | 65 | 9.2 | 14 | 3.48 |
| 144 | 4.0119 | 50 | 6.7 | 6 | 0.26 |
| 128 | 2.7085 | 65 | 6.3 | 15 | -0.16 |
| 146 | 2.6386 | 76 | 7.2 | 15 | 1.13 |
| 149 | 3.5951 | 57 | 8.7 | 15 | 1.11 |
| 133 | 3.2449 | 57 | 6.3 | 5 | 0.51 |
| 128 | 2.0715 | 85 | 6 | 15 | -0.42 |
| 148 | 2.3945 | 85 | 9.2 | 15 | 1.19 |
| 159 | 3.1872 | 69 | 5.9 | 7 | 0.20 |
| 135 | 3.2189 | 58 | 5.4 | 13 | -0.87 |
| 121 | 3.5232 | 47 | 7 | 4 | 0.42 |
| 124 | 3.0533 | 56 | 7.7 | 15 | 1.03 |
| 165 | 4.1953 | 54 | 6.9 | 12 | 0.48 |
| 133 | 2.7565 | 67 | 6.7 | 7 | 0.64 |
| 162 | 3.7028 | 60 | 6.3 | 2 | 0.09 |
| 140 | 2.5165 | 77 | 6.9 | 15 | 0.77 |
| 150 | 2.9949 | 69 | 5.8 | 15 | -0.46 |
| 147 | 1.9117 | 106 | 6.1 | 15 | 0.06 |
| 97 | 3.3206 | 40 | 7.2 | 11 | 0.42 |
| 136 | 2.7493 | 68 | 6.9 | 8 | 0.22 |
| 163 | 8.5434 | 26 | 6.6 | 13 | 0.52 |
| 147 | 1.556 | 130 | 7.6 | 13 | -0.79 |
| 135 | 2.7102 | 28 | 7.9 | 15 | 0.91 |
| 115 | 2.2044 | 72 | 6.9 | 9 | 0.51 |
| 147 | 2.8512 | 71 | 6.6 | 15 | 0.48 |
| 102 | 2.0548 | 69 | 5.8 | 5 | 0.14 |
| 112 | 1.5632 | 99 | 9.8 | 15 | 1.71 |
| 152 | 3.8058 | 55 | 6.8 | 6 | -0.01 |
| 142 | 3.4257 | 57 | 6.5 | 14 | -0.11 |
| 156 | 2.2139 | 97 | 8.7 | 13 | 1.84 |
| 136 | 2.3465 | 80 | 8 | 13 | 0.55 |
| 146 | 3.4232 | 59 | 6.4 | 6 | -0.23 |

| | | | | | |
|---|---|---|---|---|---|
| 152 | 3.8213 | 55 | 10.4 | 15 | 2.18 |
| 146 | 2.4985 | 81 | 6.6 | 2 | 0.40 |
| 112 | 2.096 | 74 | 6.3 | 15 | 0.51 |
| 148 | 2.7668 | 74 | 6.8 | 15 | 0.35 |
| 149 | 2.9914 | 69 | 8.4 | 14 | -0.11 |
| 125 | 3.1257 | 55 | 6.4 | 13 | 0.56 |
| 177 | 3.75 | 65 | 6.9 | 10 | 0.43 |
| 157 | 1.7353 | 125 | 9.7 | 15 | 2.78 |
| 140 | 2.0137 | 96 | 8.4 | 11 | 0.52 |
| 140 | 2.3481 | 82 | 7.6 | 4 | 0.82 |
| 110 | 2.9516 | 51 | 6.7 | 15 | 0.65 |
| 112 | 2.0018 | 77 | 6.3 | 6 | 0.61 |
| 164 | 2.1522 | 105 | 10.3 | 8 | 2.07 |
| 137 | 1.3226 | 143 | 8.3 | 15 | 1.48 |
| 133 | 3.1842 | 58 | 5.9 | 15 | 0.14 |
| 131 | 2.3856 | 76 | 9.1 | 15 | 1.90 |
| 130 | 4.2342 | 42 | 6.9 | 13 | 1.09 |
| 125 | 2.3015 | 75 | 8.8 | 15 | 1.28 |
| 134 | 2.9487 | 63 | 8 | 13 | 1.72 |
| 139 | 2.9867 | 64 | 11.3 | 7 | 2.19 |
| 135 | 1.4681 | 14 | 7.5 | 15 | 1.44 |
| 127 | 2.13 | 82 | 7.7 | 2 | 1.60 |
| 147 | 2.6141 | 78 | 7.7 | 2 | 1.95 |
| 148 | 3.3183 | 62 | 8 | 2 | 1.92 |

| GMI % | J_index | （MG+SD）^2 | MG mg/dl | MGmmol/l | Fundus les | DPN | Cardiovascular disease |
|---|---|---|---|---|---|---|---|
| 5.98 | 18.27 | 56.40 | 111.78 | 6.21 | 1 | 1 | 1 |
| 6.10 | 23.74 | 73.27 | 116.82 | 6.49 | 1 | 1 | 1 |
| 6.92 | 52.18 | 161.04 | 151.02 | 8.39 | 0 | 0 | 0 |
| 6.82 | 31.18 | 96.24 | 146.88 | 8.16 | 1 | 1 | 1 |
| 6.24 | 23.30 | 71.91 | 122.40 | 6.80 | 1 | 1 | 1 |
| 6.52 | 30.36 | 93.70 | 134.28 | 7.46 | 1 | 0 | 0 |
| 5.96 | 19.36 | 59.75 | 110.88 | 6.16 | 1 | 0 | 1 |
| 7.39 | 43.45 | 134.10 | 170.64 | 9.48 | 1 | 0 | 0 |
| 8.06 | 70.02 | 216.12 | 198.63 | 11.04 | 1 | 1 | 1 |
| 6.07 | 19.76 | 61.00 | 115.38 | 6.41 | 1 | 0 | 1 |
| 5.72 | 16.29 | 50.27 | 100.80 | 5.60 | 1 | 0 | 1 |
| 6.03 | 22.69 | 70.02 | 113.60 | 6.31 | 1 | 1 | 1 |
| 6.39 | 26.65 | 82.26 | 128.70 | 7.15 | 1 | 1 | 1 |
| 6.46 | 30.73 | 94.84 | 131.59 | 7.31 | 1 | 1 | 1 |
| 6.22 | 22.16 | 68.39 | 121.86 | 6.77 | 1 | 0 | 1 |
| 6.47 | 30.17 | 93.12 | 131.94 | 7.33 | 0 | 0 | 0 |
| 6.02 | 23.91 | 73.80 | 113.44 | 6.30 | 1 | 1 | 1 |
| 6.54 | 33.17 | 102.36 | 135.07 | 7.50 | 1 | 1 | 1 |
| 6.30 | 26.71 | 82.45 | 124.92 | 6.94 | 1 | 1 | 1 |
| 7.31 | 43.70 | 134.89 | 167.37 | 9.30 | 1 | 0 | 1 |
| 5.82 | 16.06 | 49.56 | 104.76 | 5.82 | 1 | 1 | 1 |
| 5.91 | 16.24 | 50.13 | 108.90 | 6.05 | 1 | 1 | 1 |
| 6.95 | 41.89 | 129.28 | 152.10 | 8.45 | 1 | 0 | 0 |
| 5.22 | 9.55 | 29.48 | 79.74 | 4.43 | 1 | 0 | 0 |
| 6.62 | 32.53 | 100.40 | 138.42 | 7.69 | 1 | 1 | 1 |
| 5.66 | 13.40 | 41.34 | 98.10 | 5.45 | 0 | 0 | 0 |
| 6.73 | 31.56 | 97.42 | 143.10 | 7.95 | 1 | 0 | 0 |
| 5.82 | 16.15 | 49.84 | 105.12 | 5.84 | 1 | 1 | 1 |
| 7.24 | 42.38 | 130.80 | 164.16 | 9.12 | 1 | 1 | 1 |
| 6.85 | 41.30 | 127.46 | 147.96 | 8.22 | 1 | 0 | 1 |
| 6.91 | 41.23 | 127.24 | 150.66 | 8.37 | 1 | 1 | 1 |
| 7.61 | 61.17 | 188.79 | 179.82 | 9.99 | 1 | 1 | 1 |
| 6.49 | 29.22 | 90.19 | 132.90 | 7.38 | 1 | 0 | 1 |
| 5.80 | 15.61 | 48.16 | 104.22 | 5.79 | 1 | 1 | 0 |
| 6.43 | 31.44 | 97.02 | 130.32 | 7.24 | 1 | 0 | 0 |
| 7.17 | 43.60 | 134.56 | 161.46 | 8.97 | 1 | 0 | 0 |
| 6.07 | 23.30 | 71.91 | 115.20 | 6.40 | 1 | 1 | 1 |
| 6.02 | 22.97 | 70.90 | 113.22 | 6.29 | 1 | 0 | 1 |
| 8.82 | 83.67 | 258.24 | 230.40 | 12.80 | 1 | 0 | 1 |
| 6.24 | 25.18 | 77.73 | 122.47 | 6.80 | 1 | 1 | 0 |
| 6.05 | 19.51 | 60.22 | 114.48 | 6.36 | 1 | 0 | 1 |
| 6.16 | 28.14 | 86.86 | 118.98 | 6.61 | 1 | 1 | 1 |
| 5.98 | 19.71 | 60.84 | 111.60 | 6.20 | 1 | 0 | 1 |
| 7.06 | 39.37 | 121.53 | 156.96 | 8.72 | 1 | 1 | 1 |
| 5.91 | 19.26 | 59.44 | 108.54 | 6.03 | 1 | 0 | 0 |
| 6.31 | 26.83 | 82.81 | 125.46 | 6.97 | 1 | 0 | 0 |
| 5.99 | 17.37 | 53.62 | 111.89 | 6.22 | 1 | 1 | 1 |

| | | | | | | | |
|---|---|---|---|---|---|---|---|
| 6.40 | 28.81 | 88.92 | 129.24 | 7.18 | 1 | 1 | 0 |
| 6.13 | 23.57 | 72.76 | 117.72 | 6.54 | 1 | 1 | 0 |
| 8.61 | 75.75 | 233.78 | 221.40 | 12.30 | 1 | 0 | 1 |
| 5.92 | 17.98 | 55.50 | 109.08 | 6.06 | 1 | 1 | 1 |
| 5.96 | 21.74 | 67.10 | 110.77 | 6.15 | 1 | 0 | 0 |
| 6.12 | 24.30 | 75.00 | 117.54 | 6.53 | 1 | 1 | 0 |
| 5.45 | 14.11 | 43.56 | 89.28 | 4.96 | 1 | | |
| 6.08 | 22.21 | 68.56 | 115.92 | 6.44 | 1 | 0 | 1 |
| 7.01 | 41.23 | 127.24 | 154.80 | 8.60 | 1 | 0 | 1 |
| 6.05 | 20.63 | 63.68 | 114.66 | 6.37 | 1 | 0 | 1 |
| 6.14 | 21.21 | 65.45 | 118.44 | 6.58 | 1 | 1 | 1 |
| 6.40 | 27.42 | 84.64 | 129.06 | 7.17 | 1 | 0 | 0 |
| 6.31 | 25.15 | 77.62 | 125.46 | 6.97 | 1 | 0 | 1 |
| 8.86 | 78.26 | 241.55 | 232.20 | 12.90 | 1 | 0 | 1 |
| 9.01 | 93.93 | 289.91 | 238.21 | 13.23 | 1 | 1 | 1 |
| 8.73 | 82.32 | 254.08 | 226.44 | 12.58 | 0 | 1 | 1 |
| 6.03 | 18.03 | 55.66 | 113.81 | 6.32 | 1 | 0 | 1 |
| 5.91 | 17.93 | 55.35 | 108.72 | 6.04 | 0 | 1 | 1 |
| 6.01 | 18.86 | 58.22 | 112.86 | 6.27 | 1 | 1 | 1 |
| 6.49 | 27.60 | 85.19 | 133.02 | 7.39 | 1 | 0 | 1 |
| 6.44 | 29.86 | 92.16 | 130.86 | 7.27 | 1 | 0 | 1 |
| 5.61 | 14.16 | 43.69 | 96.12 | 5.34 | 1 | 0 | 1 |
| 6.07 | 22.43 | 69.22 | 115.38 | 6.41 | 1 | 0 | 0 |
| 6.17 | 25.43 | 78.50 | 119.70 | 6.65 | 1 | 0 | 1 |
| 6.13 | 27.36 | 84.46 | 117.90 | 6.55 | 1 | 1 | 1 |
| 6.52 | 27.07 | 83.54 | 134.10 | 7.45 | 1 | 0 | 1 |
| 6.99 | 43.30 | 133.63 | 153.90 | 8.55 | 1 | 1 | 1 |
| 6.52 | 32.95 | 101.70 | 134.04 | 7.45 | 1 | 0 | 1 |
| 8.22 | 63.69 | 196.56 | 205.38 | 11.41 | 0 | 1 | 1 |
| 6.06 | 23.03 | 71.09 | 115.15 | 6.40 | 1 | 1 | 1 |
| 6.43 | 27.87 | 86.01 | 130.48 | 7.25 | 1 | 1 | 1 |
| 6.94 | 41.64 | 128.52 | 151.57 | 8.42 | 1 | 0 | 1 |
| 5.77 | 15.57 | 48.05 | 102.64 | 5.70 | 1 | 1 | 1 |
| 8.02 | 63.81 | 196.95 | 196.80 | 10.93 | 1 | 1 | 0 |
| 6.02 | 19.99 | 61.69 | 113.12 | 6.28 | 1 | 1 | 0 |
| 6.12 | 21.15 | 65.29 | 117.35 | 6.52 | 1 | 0 | 0 |
| 7.61 | 48.42 | 149.44 | 179.69 | 9.98 | 1 | 0 | 1 |
| 6.30 | 26.45 | 81.65 | 124.84 | 6.94 | 1 | 0 | 1 |
| 7.09 | 38.90 | 120.05 | 157.99 | 8.78 | 0 | 0 | 0 |
| 7.12 | 40.35 | 124.54 | 159.10 | 8.84 | 1 | 0 | 1 |
| 6.86 | 45.02 | 138.96 | 148.24 | 8.24 | 1 | 1 | 1 |
| 6.44 | 28.29 | 87.31 | 130.87 | 7.27 | 0 | 0 | 1 |
| 6.10 | 25.68 | 79.25 | 116.73 | 6.48 | 1 | 0 | 0 |
| 5.71 | 17.96 | 55.42 | 100.39 | 5.58 | 1 | 1 | 1 |
| 8.08 | 68.36 | 210.98 | 199.61 | 11.09 | 1 | 1 | 1 |
| 5.98 | 18.08 | 55.80 | 111.60 | 6.20 | 1 | 0 | 1 |
| 8.39 | 95.41 | 294.47 | 212.22 | 11.79 | 1 | 1 | 0 |
| 6.93 | 40.35 | 124.55 | 151.23 | 8.40 | 1 | 1 | 1 |

| | | | | | | | |
|---|---|---|---|---|---|---|---|
| 6.04 | 21.51 | 66.40 | 114.13 | 6.34 | 1 | 1 | 1 |
| 6.34 | 26.89 | 82.99 | 126.54 | 7.03 | 1 | 0 | 0 |
| 5.97 | 18.57 | 57.30 | 111.06 | 6.17 | 1 | 1 | 1 |
| 6.36 | 26.53 | 81.88 | 127.32 | 7.07 | 1 | 0 | 1 |
| 7.40 | 47.78 | 147.46 | 171.01 | 9.50 | 1 | 0 | 1 |
| 5.55 | 12.71 | 39.24 | 93.74 | 5.21 | 0 | 0 | 1 |
| 5.69 | 16.05 | 49.54 | 99.47 | 5.53 | 1 | 1 | 1 |
| 6.78 | 32.73 | 101.01 | 144.98 | 8.05 | 1 | 0 | 1 |
| 5.77 | 13.80 | 42.59 | 102.94 | 5.72 | 1 | 1 | 1 |
| 6.46 | 28.35 | 87.50 | 131.67 | 7.31 | 1 | 0 | 0 |
| 6.18 | 23.73 | 73.24 | 119.99 | 6.67 | 1 | 0 | 1 |
| 6.05 | 19.88 | 61.36 | 114.74 | 6.37 | 1 | 0 | 1 |
| 8.34 | 72.32 | 223.20 | 210.31 | 11.68 | 1 | 0 | 1 |
| 7.37 | 48.82 | 150.68 | 169.58 | 9.42 | 1 | 0 | 0 |
| 5.72 | 16.11 | 49.72 | 100.91 | 5.61 | 1 | 0 | 0 |
| 6.44 | 25.68 | 79.26 | 130.88 | 7.27 | 1 | 0 | 1 |
| 6.46 | 26.19 | 80.83 | 131.79 | 7.32 | 1 | 0 | 1 |
| 6.07 | 21.28 | 65.67 | 115.19 | 6.40 | 1 | 0 | 1 |
| 7.59 | 48.95 | 151.07 | 178.93 | 9.94 | 1 | 0 | 1 |
| 5.79 | 17.79 | 54.91 | 103.55 | 5.75 | 1 | 1 | 1 |
| 6.42 | 23.87 | 73.67 | 130.22 | 7.23 | 1 | 0 | 0 |
| 8.01 | 56.77 | 175.21 | 196.48 | 10.92 | 1 | 0 | 1 |
| 5.70 | 13.04 | 40.25 | 99.86 | 5.55 | 1 | 1 | 1 |
| 6.27 | 24.83 | 76.63 | 123.91 | 6.88 | 1 | 1 | 1 |
| 6.58 | 26.05 | 80.39 | 136.66 | 7.59 | 0 | 1 | 1 |
| 6.67 | 30.64 | 94.58 | 140.62 | 7.81 | 1 | 0 | 0 |
| 6.42 | 28.58 | 88.21 | 130.06 | 7.23 | 1 | 0 | 0 |
| 6.06 | 22.65 | 69.92 | 114.97 | 6.39 | 1 | 1 | 1 |
| 6.21 | 23.77 | 73.35 | 121.13 | 6.73 | 1 | | |
| 6.13 | 23.90 | 73.75 | 118.06 | 6.56 | 1 | 0 | 0 |
| 6.26 | 22.32 | 68.88 | 123.42 | 6.86 | 0 | 0 | 0 |
| 6.04 | 19.35 | 59.71 | 114.27 | 6.35 | 1 | 1 | 1 |
| 6.78 | 39.65 | 122.36 | 145.24 | 8.07 | 1 | 1 | 0 |
| 6.68 | 28.66 | 88.46 | 140.74 | 7.82 | 1 | 1 | 0 |
| 6.08 | 21.88 | 67.54 | 115.93 | 6.44 | 1 | 1 | 1 |
| 8.39 | 76.44 | 235.93 | 212.25 | 11.79 | 1 | 0 | 1 |
| 6.99 | 44.26 | 136.61 | 153.85 | 8.55 | 0 | 0 | 0 |
| 6.39 | 26.80 | 82.70 | 128.63 | 7.15 | 1 | 0 | 1 |
| 6.12 | 22.28 | 68.76 | 117.39 | 6.52 | 1 | 0 | 0 |
| 5.66 | 13.14 | 40.55 | 98.30 | 5.46 | 1 | 1 | 1 |
| 8.09 | 64.53 | 199.16 | 199.65 | 11.09 | 1 | 1 | 1 |
| 6.81 | 33.50 | 103.39 | 146.39 | 8.13 | 1 | 0 | 1 |
| 6.61 | 31.96 | 98.66 | 137.88 | 7.66 | 1 | 1 | 0 |
| 6.86 | 39.81 | 122.86 | 148.51 | 8.25 | 1 | 0 | 1 |
| 7.45 | 53.16 | 164.09 | 172.87 | 9.60 | 1 | 1 | 0 |
| 6.63 | 33.47 | 103.30 | 138.66 | 7.70 | 1 | 0 | 0 |

| | | | | | | | |
|---|---|---|---|---|---|---|---|
| 8.22 | 56.34 | 173.90 | 205.27 | 11.40 | 1 | 0 | 0 |
| 6.20 | 20.37 | 62.86 | 120.74 | 6.71 | 0 | 0 | 1 |
| 5.79 | 17.01 | 52.51 | 103.54 | 5.75 | 1 | 1 | 0 |
| 6.45 | 30.47 | 94.03 | 131.42 | 7.30 | 1 | 1 | 1 |
| 8.51 | 89.42 | 275.99 | 217.32 | 12.07 | 1 | 0 | 1 |
| 5.84 | 18.12 | 55.94 | 105.84 | 5.88 | 1 | 0 | 0 |
| 6.47 | 26.41 | 81.53 | 131.93 | 7.33 | 1 | 0 | 1 |
| 6.92 | 49.00 | 151.23 | 151.09 | 8.39 | 1 | 1 | 0 |
| 7.88 | 58.47 | 180.47 | 191.26 | 10.63 | 1 | 1 | 0 |
| 6.78 | 38.97 | 120.27 | 145.11 | 8.06 | 1 | 0 | 0 |
| 6.05 | 21.74 | 67.10 | 114.35 | 6.35 | 0 | 0 | 1 |
| 5.69 | 14.33 | 44.23 | 99.70 | 5.54 | 1 | 0 | 1 |
| 8.23 | 70.21 | 216.69 | 205.69 | 11.43 | 1 | 1 | 0 |
| 6.82 | 36.39 | 112.31 | 146.58 | 8.14 | 1 | 0 | 0 |
| 5.76 | 15.54 | 47.97 | 102.59 | 5.70 | 1 | 0 | 1 |
| 7.20 | 49.15 | 151.69 | 162.56 | 9.03 | 1 | 1 | 1 |
| 5.81 | 18.88 | 58.27 | 104.51 | 5.81 | 1 | 0 | 1 |
| 7.52 | 55.34 | 170.80 | 176.07 | 9.78 | 1 | 1 | 0 |
| 6.28 | 26.64 | 82.21 | 124.21 | 6.90 | 1 | 0 | 0 |
| 9.11 | 77.32 | 238.65 | 242.35 | 13.46 | 1 | 1 | 1 |
| 6.06 | 21.38 | 66.00 | 114.92 | 6.38 | 1 | 0 | 1 |
| 6.10 | 21.47 | 66.26 | 116.64 | 6.48 | 1 | 0 | 1 |
| 5.75 | 14.47 | 44.66 | 101.99 | 5.67 | 1 | 0 | 1 |
| 6.08 | 25.49 | 78.67 | 115.60 | 6.42 | 1 | 0 | 1 |
